# Digital Intervention in Loneliness in Older Adults: Qualitative Analysis of User Studies

**DOI:** 10.2196/42172

**Published:** 2023-01-27

**Authors:** Avelie Stuart, Ronnie Jieru Yan, Lydia Jo Harkin, Dmitri Katz, Clifford Stevenson, Vikram Mehta, Emilie Giles, Catherine Talbot, Daniel Gooch, Mohamed Bennasar, Tara Self, Bashar Nuseibeh, Blaine Price

**Affiliations:** 1 Department of Psychology University of Exeter Exeter United Kingdom; 2 Department of Psychology Nottingham Trent University Nottingham United Kingdom; 3 Computing and Communications The Open University Milton Keynes United Kingdom; 4 Graphic Design School of Arts and Communication Arts University Bournemouth Bournemouth United Kingdom; 5 Department of Psychology Bournemouth University Bournemouth United Kingdom; 6 Lero - The Irish Software Research Centre University of Limerick Limerick Ireland

**Keywords:** loneliness, older adults, digital connections, reflection, social identity, user-centered design, social network, well-being apps

## Abstract

**Background:**

Loneliness is a significant well-being issue that affects older adults. Existing, commonly used social connection platforms do not contain facilities to break the cognitive cycle of loneliness, and loneliness interventions implemented without due processes could have detrimental effects on well-being. There is also a lack of digital technology designed with older adults.

**Objective:**

We aimed to iteratively design a user-centered smartphone app that can address loneliness in older adults. The aim of this study was to investigate the loneliness-related psychological processes that our conceptual smartphone app promotes. We also identified the emergent needs and concerns that older adults raised regarding the potential benefits and detriments of the app.

**Methods:**

We used technology probes to elicit older adults’ reflections on the concept of using the app in 2 studies as follows: concept focus groups (n=33) and concept interviews (n=10). We then conducted a prototype trial with 1 week of use and follow-up interviews (n=12).

**Results:**

Thematic analysis explored the experiences and emergent challenges of our app through the design process. This led to the development of 4 themes as follows occurring in all 3 qualitative data sets: reflection on a digital social map is reassuring; app features encourage socializing; the risk of compounding loneliness; and individuals feel more control with mutual, socially beneficial activities.

**Conclusions:**

Smartphone apps have the potential to increase older adults’ awareness of the richness of their social connections, which may support loneliness reduction. Our qualitative approach to app design enabled the inclusion of older adults’ experiences in technology design. Thus, we conclude that the older adults in our study most desired functionalities that can support mutual activities and maintain or find new connections rather than enable them to share an emotional state. They were wary of the app replacing their preferred in-person social interaction. Participants also raised concerns about making the user aware of the lack of support in their social network and wanted specific means of addressing their needs. Further user-centered design work could identify how the app can support mutual activities and socializing.

## Introduction

### Background

Health care technology for older adults has largely focused on physical health and overlooked mental health issues [1]. Loneliness is one such mental health issue that can affect older adults and has a negative effect on their physical health [2]. Although numerous digital platforms (eg, games, exercise programs, and health monitoring systems) include a social networking element [3], there is a lack of research on designing digital technologies with the express intent to address loneliness [4], and interventions specifically targeting older adults have been inconclusive [5,6].

Our work aimed to contribute to addressing this research gap using a multidisciplinary approach focusing on the suitability of our digital intervention for older adults, as well as an understanding of the psychological and behavioral science theories of loneliness. We investigated whether a targeted digital intervention, in the form of a smartphone app, could be helpful in mitigating loneliness in older adults. The following section outlines how the prototype app integrates novel insights from theoretical and user-centered design (UCD) approaches.

### The Cognitive and Behavioral Aspects of Loneliness for Older Adults

Loneliness is the *felt* lack of social relationships [7]. People who experience frequent loneliness tend not to seek social contact, as they often experience low self-efficacy, social anxiety, low social support, and depression [4,8-10] and have a reduced ability to understand emotions and engage in positive emotion regulation [11,12]. Similarly, if older adults identify with a negative perception of being *old* [13,14], they tend to use less effective coping strategies for their mental health [15] and experience threatened self-efficacy and autonomy in social relationships [13]. This cycle involves a complex exchange of lived experience of age and cognitive barriers to social connection–seeking behavior, which requires intervention to change.

Existing, routinely used social connection platforms such as Facebook or Zoom do not contain facilities designed to break the cognitive cycle of loneliness such as recognition of loneliness-related low mood or explicit promotion of activities encouraging social interaction and positive mood. Although there is an association between internet use and reduced loneliness among older adults [16], simply providing people who are lonely with access to the internet or social technology is unlikely to reduce loneliness [17]. Loneliness interventions implemented without deriving hypotheses or testing with users can even be detrimental to well-being [1,18]. Loneliness is, therefore, a pervasive problem that requires concerted research to address.

### Interventions for Loneliness

The types of digital interventions for addressing loneliness developed to date include computer skills training [19], social networks [20], internet-based classes [21,22], messaging [6], digitized formal therapies, general mental health tools, and web-based social spaces [4]. One effective psychological theory-based intervention [23] trained older adults using Facebook and found improved cognitive capacities in trainees supported by self-maintenance, improved self-competence, and increased social engagement. However, many technology-supported loneliness interventions have failed to build on previous theories or research [6,24,25].

Our approach takes inspiration from social identity and network mapping exercises [26-29] using the Social Identity Approach to Health conceptual framework, which provides guidance for developing older people’s positive identities and enhanced well-being when they have undergone aging-related life changes [30]. An intervention developed from the Social Identity Approach to Health involves visually mapping out social group memberships and developing strategies for generating and sustaining these group memberships [31,32]. These structured approaches provide a psychological solution to counter the entrenched effects of loneliness [23,32]. For older adults especially, it may be helpful to shift from focusing on *deficits* experienced in older adulthood to *positive* identities and connections available in older adulthood [33].

Digital versions of this mapping exercise have been shown to be usable and effective in the short term [28,34] but have not been adapted for ongoing use and are not specifically tailored to older adults. Our research furthers the idea of social mapping, integrating it with daily personal reflection records while maintaining social and emotional well-being. We also incorporated a UCD with our target population of older adults, so the intervention is more likely to be usable, effective, and enjoyable to use.

### UCD Approach

Older adult users often do not use technology, because they are excluded from technological design [1]. Likewise, many loneliness interventions have been unsuccessful owing to not actively involving older adults in the design of the intervention [18,29,35]. Furthermore, users vulnerable to loneliness may have expectations of an intervention that will exacerbate feelings of isolation and loneliness if they are not met [36,37]. Therefore, interventions specifically designed *with,* rather than *for,* older adults are needed [38].

UCD is an approach that advocates that designing technology with key stakeholders is essential to make the tool fit for purpose, usable, effective, and engaging [1,39]. UCD entails iterative cycles of empathizing with and understanding users, designing probes or prototypes, assessing users’ experiences of using prototypes, and improving prototypes, during which users are continually consulted [39]. The UCD process tends to result in systems in which users will engage more willingly [40], in part because they are more enjoyable to use. However, UCD does not always involve behavioral science theory [41]; thus, the long-term efficacy of the technology can be difficult to establish. Our research involves consulting older adults and considering their needs; however, it also refers to a theory that could result in an efficacious product later in development [35].

### Goal of Study

Our work aimed to explore older adults’ needs and experiences resulting from discussing the idea of a smartphone app (in focus groups and interviews) and from using our prototype smartphone app (in a 1-week trial). The prototype app design applies theories of loneliness and aging by uniquely merging self-reflection technologies [42-44] with social support functionality and social circle mapping to promote awareness of social connectivity [31,32]. The aim of this study was to investigate the loneliness-related psychological processes that our conceptual smartphone app promotes. We also identify the emergent needs and concerns that older adults raise regarding the potential benefits and detriments of the app.

We present a qualitative approach that can aid in understanding how technology influences people’s experiences and how users interpret the role of technology in behavior change processes [41]. We report a thematic analysis of interview data from 3 user studies using technology probes [45] with increasing fidelity.

## Methods

### Design

**Figure 1:**
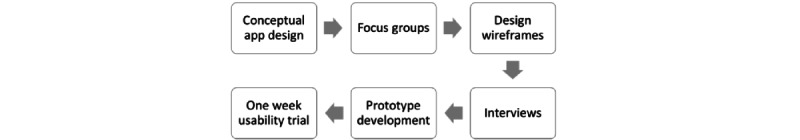
Iterative user study and app design process.

This study used a qualitative design with 3 user studies building on each other. The requirement for a loneliness-reducing platform originated from the participatory design workshops conducted by the research team in 2019. We then conducted a literature review and identified gaps in existing loneliness interventions [38]. Forming the analysis in this paper are study 1—focus groups, study 2—interviews, and study 3—a usability trial (see Figure 1 for depiction of this process). All studies were designed following the guidance of qualitative methods [46,47] and UCD [35].

### Procedure

All data were collected remotely using video call software (Teams or Zoom) owing to COVID-19 social distancing restrictions. Study 1 entailed semistructured focus groups (average duration 106 minutes) facilitated by a member of the research team from October to December 2020. Focus groups explored shared perceptions of a conceptual illustration of the app using a low-fidelity technology probe technique [45] (the focus groups also formed part of a separate study on the lived experience of the COVID-19 pandemic).

Feedback was implemented into wireframes that were used with a video explaining the concept as a more detailed probe within study 2, where we ran semistructured interviews in January 2021 (average duration 56.1 minutes). A new team member facilitated the interviews.

In the final stage, wireframes were implemented in a working prototype app that was used in study 3—a 1-week usability test with follow-up interviews conducted in July 2021. For this study, participants received a web-based onboarding meeting where they received basic training on how to download, log in to, and use the app features. Participants were asked to use the app as part of their daily routine for 1 week. A team member facilitated the semistructured interviews (average duration 68.5 minutes). See the data availability statement for all interview schedules and materials.

### Recruitment

Recruitment of participants for studies 1 and 3 was through the Nottingham Trent Aging Panel—an institutionally managed resource running since 2016 and regularly advertised through local community groups, newsletters, and community centers. Participants from study 2 were convenience samples who were personal contacts of the team, had participated in unrelated previous studies, or belonged to a University of the Third Age club for older adults.

Participants were eligible if they were older than the age of 50 years; many researchers (eg, [48]) and organizations (eg, [24,49]) view *older adulthood* as starting at the age of 50. Some people of this age may not consider themselves old; however, 50 years old is when some transitions into later life occur as follows: (1) people may take voluntary early retirement or be forced into retirement, perhaps because it is difficult to find employment; (2) people begin to plan for their retirement; and (3) people start to experience physical or cognitive decline.

In study 1, participants did not have to be smartphone users. In study 3, we attempted to recruit people across age ranges (50-74 or 75+ years), gender (male or female), reported loneliness levels (high or low), and comfort with technology (high or low).

### Materials

#### Study 1: Concept Video and Focus Groups

Participants watched a prerecorded video (Multimedia Appendix 1) describing a *person-centered social and healthcare support network*. We illustrated a user persona of Elizabeth, a fictional woman with a variety of interests and family and friends but also had medical conditions that restricted her activities (especially during COVID-19); she had also undergone other age-related changes in her life. Elizabeth used the app to keep track of her loneliness and her social contacts and activities that have helped her mood, to obtain suggestions of activities she could do, and to choose whether to add people to her digital social map and share well-being data and social activities with them. At the end of the video, a series of questions appeared, and the facilitator engaged the participants in a semistructured discussion.

#### Study 2: Wireframe Design

**Figure 2:**
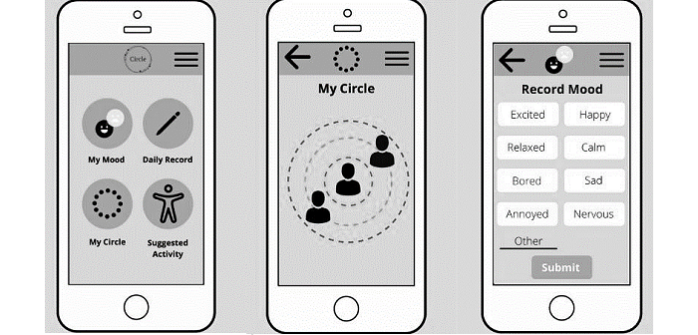
Wireframes of the proposed app concept as used in study 2.

We updated the video explaining the app concept based on the feedback from the first study. In addition, wireframes (Multimedia Appendix 2) were created, guided by the unpublished findings of 2 previous participatory design workshops, the teams’ ongoing research on mood logging [50,51], as well as reviewed literature on loneliness and social connections [28,31,34,38]. The resulting wireframe prototype had 4 main functions as follows: mood logger, daily record logger, digital social map, and suggested activity features. It also had a gamification and reward system for logging, which participants roundly rejected; therefore, this was not included in the next prototype. The facilitator talked participants through the screens and engaged them in a discussion (interview schedule in Multimedia Appendix 3). Participants expressed most interest in mood and social aspects; therefore, we focused on these features in the development of the working prototype. Figure 2 shows the key wireframes.

#### Study 3: Prototype Design

The wireframes were updated in Figma (Figma Inc) to allow for improved collaboration within the design team and easier integration with the development team working in Ionic and Angular. Apps were then exported to iOS and Android.

**Figure 3:**
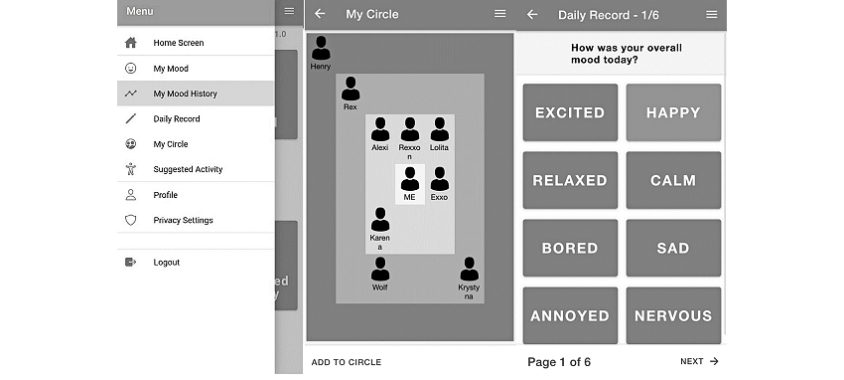
Screenshots of the prototype app used in study 3.

The main features of the prototype are shown in Figure 3 (see Multimedia Appendix 4 for all screens). Participants could enter their moods; complete a daily account of activities, social contact, and where they had spent their time that day; and enter people into their digital social map (with an inner, middle, and outer layer). The suggested activity, profile, and history pages are shown as options but are not active. The participants discussed (interview schedule in Multimedia Appendix 5) their thoughts on what these features could contain.

All videos, wireframes, interview schedules, and screenshots are linked in the data availability statement.

### Ethics Approval

The Open University (HREC/3776/Mehta) and Nottingham Trent University (STEVENSON 2020/220 and 2021/346) institutional ethical review boards approved all the stages of data collection.

### Informed Consent

For studies 1 and 2, information was emailed to participants, and consent was obtained verbally via a video conferencing platform (Teams or Zoom). For study 3, information and consent were obtained through a web-based survey. Participants in all 3 studies received GBP £20 (US $24.55) Amazon vouchers. All participants’ names were changed to numbers (eg, P1 and P2) to protect anonymity.

### Qualitative Analysis

Qualitative data from the 3 user studies were analyzed by 3 coders in NVivo (QSR International) using Braun and Clarke’s Thematic Analysis [52] and guided by previous research on loneliness (eg, [31,34,53]). The focus of the analysis was on the psychological experiences of using the app and its role as a digital intervention for feelings of loneliness and social connectedness (rather than an analysis of interface usability). The focus group study data were analyzed first, then combined with the interview study data, and finally the thematic structure was explored within the usability trial data.

**Figure 4:**
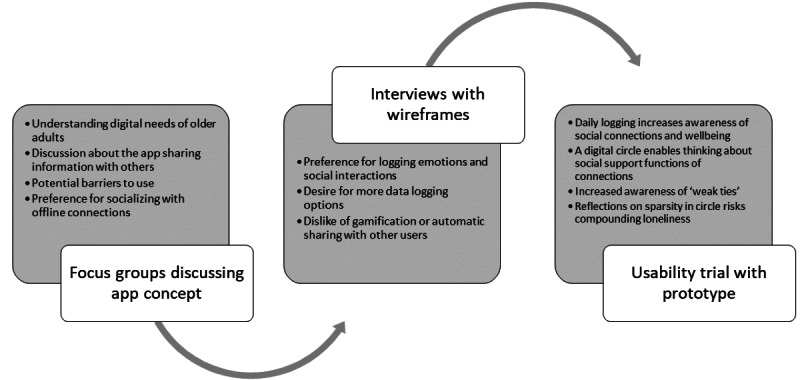
Depiction of key insights from the 3 studies.

Figure 4 presents the key insights derived from each study. We allowed the analysis to inform the iterative design and final themes to illustrate the experience of the process of using the app. Thus, we presented the themes commonly found across all data sets as a single results section. The interpretation of the results in relation to theory and previous research is presented in the *Discussion* section.

## Results

### Participants

Table 1 shows the participant demographics.

**Table 1:** Participant information.

Characteristic			Study 1: focus groups (11 groups; n=33)		Study 2: concept interviews (n=10)		Study 3: usability trial (n=12)
**Age (years)**							
	Mean (SD)	72.1 (5.12)^a^		71.5 (10.27)		70.4 (6.96)	
	Range	52-79^a^		53-81		62-87	
**Sex, n (%)**							
	Female	25 (76)		6 (60)		6 (50)	
	Male	8 (24)		4 (40)		6 (50)	
**Ethnicity/nationality, n (%)**							
	White British	33 (100)		4 (40)		12 (100)	
	Mixed race	0 (0)		1 (10)		0 (0)	
	White African	0 (0)		1 (10)		0 (0)	
	British/English	0 (0)		2 (20)		0 (0)	
	Canadian	0 (0)		1 (10)		0 (0)	
	White	0 (0)		1 (10)		0 (0)	
**Relationship/living status, n (%)**							
	Married, living with partner	15 (45)		2 (20)		0 (0)	
	Single, living with parents	1 (3)		0 (0)		0 (0)	
	Divorced, living alone	9 (27)		0 (0)		0 (0)	
	Widow, living alone	5 (15)		0 (0)		0 (0)	
	Separated, living with family	1 (3)		0 (0)		0 (0)	
	Single, living alone	2 (6)		0 (0)		0 (0)	
	Living alone	0 (0)		7 (70)		0 (0)	
	Married, living apart	0 (0)		1 (10)		0 (0)	
	Married/partnership	0 (0)		0 (0)		9 (75)	
	Separated	0 (0)		0 (0)		1 (8)	
	Never married/registered partnership	0 (0)		0 (0)		1 (8)	
	Other	0 (0)		0 (0)		1 (8)	

^a^Plus 1 younger adult aged 27 years.

### Themes

The analysis generated 4 themes across the 3 studies, which are presented below with supporting extracts to illustrate the source of data and participant demographic details.

#### Theme 1: Reflection on a Digital Social Map Is Reassuring

Participants across the focus groups, concept interviews, and usability trial were interested in functions within the digital intervention that could encourage logging interactions, mood, and well-being data. They wanted to log social information meaningful to them, such as important activities with friends and social groups. This theme evolved over the data collection points; initially, in the focus group study, participants were open to an app that showed their social connections but were averse to sharing this information with others. Participants explained that revisiting the history of their recordings and viewing their digital social map would facilitate self-reflection on their existing connections. Furthermore, participants offered that this would be beneficial to lonely individuals, as it would allow them to feel reassured about their social connections. Being able to view a map of the people with whom they interact could counter the tendency to take actual connections for granted, thereby alleviating loneliness:

...We tend, perhaps to take them for granted and one thing the app would be to concentrate your mind on the fact that A or B did actually talk to you which you might have just dismissed as of no consequence. So, the app does draw your attention to the perhaps of the value of your circle of acquaintances rather more than otherwise...

Usability Trial, P6, male, 87 years

The digital social map encourages reflection on the nature of their supportive relationships with others. Family members were commonly described as suitable for the inner or middle circle of the map and were identified as those with whom users had frequent contact and emotional support. In addition, several participants classified close friends in the inner circle, because those friends were able to provide social interactions and build mutually supportive relationships. For some participants such as P6, once they reflected on their connections as close in their digital social map, they started thinking of ways they could support and help their friends and family:

...automatically on the inner one would be probably my family, I speak to my dad. And it’s quite interesting because it would be he’s somebody that I support, but also in there, I would have my best friend who is somebody who supports me. So it’s quite interesting how you were saying about, you know, the app’s to support people, but it’s also for you to support others. So I'd have my best friend and have my father in that.

Concept Interviews, P6, female, 53 years

Participants also found reassurance from the idea of entering their social connections into the middle and outer circles. These findings predominantly came from the 1-week trial, in which participants had time to reflect on the importance of their weaker ties. Participants commonly added social, work, neighborhood, and voluntary groups to their middle or outer circles. They explained that their decision to place people in the middle or outer circle was based on the frequency of contact they had with them and the roles that participants took within these groups:

So, for me, U3A [University of the Third Age] is something that we are part of and there is something at least once a week that either we both join in with we actually run the (anonymized). We’re both on the committee and we did so we had a committee meeting... I would put that in the intermediate circle.

Usability Trial, P11, female, 69 years

For these older adults, voluntary and active groups brought a sense of usefulness and meaning into their digital social maps. In addition, many participants entered into their outer circle if they provided them with some exchange of practical support such as carers or neighbors. For people in the outer circle, participants were less open to the concept of using the app to increase the intimacy of these connections by sharing private lives or emotions. However, reflecting on these outer-circle connections could provide a feeling of security and support, as P9 described:

...let’s just say you live alone and you’ve got home care staff coming in, you’ve got that daily contact every day but for some people that would give them an awful lot of security and support...

Usability Trial, P9, female, 67 years

However, some participants were unsure if the intervention would help to increase their awareness of social relationships, because they felt that they already possessed this insight or were already satisfied with their social contacts and considered themselves self-reliant. These participants said that they preferred not to map their circles in the app:

I don’t think that I would find it particularly useful...maybe because I don’t think that I’ve got particularly mental health issues or issues with loneliness I’ve always been a person who has been quite not self-assured but self-reliant and I don’t actually dislike my own company.

Usability Trial, P11, female, 69 years

In summary, this theme illustrates how mapping one’s existing connections encourages social insights and reassurance of the ways in which one can both give and receive social support. Further functionality in the app that allows people to define the parameters of their relationships (eg, classifying emotional and practical support, the direction of support provision, and their role within a group) may help them further explore the value of having a mixture of close and weaker connections within their social network. However, for people who do not consider themselves lonely, mapping may need to provide further value to be useful.

#### Theme 2: Features to Encourage Socializing

Most participants wanted the app to focus on encouraging more socialization. The participants explained that in-person connections were particularly valuable to them. Thus, they wanted an app that emphasized socializing with people to whom they had strong connections and their existing group memberships. When considering the design of the app, participants in the focus groups suggested that an app could potentially help build a supportive network if members of the network use the digital platform together. As suggested by focus group 10, this support network could benefit isolated older members of the family and reassure members to support them:

And, Granny can do it [use the app]. She’s 91... Mum can keep an eye to make sure Granny’s still okay. So, you know, so she gets that. So, if it’s something of that nature for that age group then yeah. It keeps her in touch with the family...

Focus group 10, P31, female, 78 years

In the focus group study, participants were keen to highlight that as older adults, web-based contact was less valuable than in-person contact. In addition, they placed high value on location-based groups (eg, volunteering groups and neighborhoods) and would like the app to allow them to continue existing conversations and share similar experiences. In the concept interview study, P6 suggested that in a working app, she would hope to share rewarding but emotionally challenging experiences with people she volunteered with to enable them to support each other:

I’ve got friends that I’ve made, who are more like colleagues really, but we have more in interest because of the volunteer the food parcels that I’m doing, but it would be quite nice checking in with them. And for them, they’re the sort of people who I can talk with who I perhaps wouldn’t talk about things with my close friends, because it’s slightly different...

Concept Interviews, P6, Female, 53 years

Across the studies, participants were cognizant of existing technological platforms for communication such as WhatsApp and Facebook. They also retained traditional methods of communication such as face-to-face meetings or phone calls. They emphasized that any new technology would need to offer a unique function and that changes to *how* they communicate with others could threaten their connections. Thus, to avoid any detriment to the relationships between social groups, the app should supplement rather than replace existing methods of communication, and the supplement should be novel:

We have many What’s App groups which is a similar thing a chat thing and it’s many people use the What’s App group. I see that a step to this. So, if they are using a What’s App group this is a similar thing maybe with a different function.

Usability Trial, P12, female, 75 years

My friends know they can phone me if they are feeling low, they can phone me at any time of the day they know that and I would be there for them. They don’t need an app.

Focus Group 3, P14, female, 74 years

Overall, participants envisaged an app that encouraged social interaction, expanded in-person connections and contacts, and allowed ways for people to support each other. However, the app must add value beyond existing strategies and digital platforms.

#### Theme 3: Risk of Compounding Loneliness

The participants raised concerns about the features of a digital intervention for older adults that could risk compounding loneliness. These insights illustrate the key features to avoid in an app for older adults. One proposal by the researchers was that sharing mood with others via the app might be a way to ask for support and address loneliness. On the one hand, many participants were open to mood logging akin to diary keeping for personal reflection. However, the concept of sharing a mood with others is met with resistance. Participants explained that web-based contact could feel “artificial” (Focus Group 7, P26, Female, 71 years), and others said that as older adults, they were not socialized to share their moods in this way:

... but I don’t know how much take-up you would get from if it’s saying an ‘older generation’ getting the older, certainly the older males to put into an app how they were feeling I don’t know maybe that’s just me you might have some resistance there I think unless you can disguise it somehow.

Usability Trial, P9, female, 67 years

In addition, several participants were concerned that if older adults had very few people to enter in their digital social map, they might feel lonely. For instance, a lack of inner circle contacts might suggest few close connections and restricted access to emotional support. As P9 illustrated, an imbalance toward social contacts on the outer social circle could “make you feel worse” and exacerbate concerns that one’s social network is inadequate.

...if you sort of start to ask people to put their sort of circle of friends in and they can’t think of anybody then actually, even though they have got contacts, it could make people feel worse...it says how many people have you got in your circle and you’ve put the milkman you know or the shopkeeper then it would make you feel worse.

Usability Trial, P9, female, 67 years

In summary, many participants were wary of a lack of response from other users, a risk of feeling greater loneliness owing to sparse maps, or a feeling of artificiality created by sharing in an app. These feelings contributed to loneliness. To counter this, participants suggested that rather than sharing moods, the app could provide features to encourage socializing, for instance, via suggestions of groups to join or intervention guidance aimed at the individuals themselves on how to increase social connections to others.

#### Theme 4: Feeling More Control With Mutual, Socially Beneficial Activities

Participants were particularly enthusiastic about the possibility that the app might suggest activities and encourage reciprocal action when engaging in activities. As older adults, most participants placed importance on remaining active members of their communities in their interest groups, and they wanted to be participants in their social relationships rather than a burden. They also wanted the app to allow them to engage in activities such as joining a group that meets their interests (eg, contacting grandchildren, joining a social group, and participating in social events based on shared expertise). For instance, P4 from the usability trial expressed an interest in engaging with different organizations that would bring together similar-minded older adults via an app feature that could suggest activities:

I was sort of thinking things in [Suggested Activity] might be clubs and societies for people of my age and older, maybe help services or local government services that kind of thing.

Usability Trial, P4, male, 62 years

Participants also explained that where the app could aid interaction between individuals, it would be important to function as a mutually supportive network. For these participants, being active meant taking on a supportive role in the circle instead of only being a recipient of support. In other words, they did not want to use an app that made them feel like a burden on those around them:

...when [another participant] says burdening or over burdening, you’re, if you’re telling somebody how you feel you’re implicitly putting something on them an expectation that they should do something about it for you.

Focus Group 4, P15, female, 75 years

As P12 explained below, if an app was mutually supportive, it would help them see their local area or groups as mutually supportive. Mutual support was described as being able to reach out to others and to offer conversations or meetings that did not necessarily need to be purely web-based but could be facilitated by the app:

I think if it’s an interactive sort of mutually supportive area I think that could be very useful just I am assuming, assuming that the idea is that it’s there to reach out to people to say you know we’re here we can this, we can that do you want to chat, you want a coffee you know that kind of thing...

Usability Trial, P12, female, 75 years

Overall, our participants emphasized an app that would allow them to take an active role in the groups and relationships they found valuable. An active digital intervention for older adults should promote reciprocal relationships and offer meaningful suggestions to find new or related interest groups with some connection to offline activities and interactions.

## Discussion

### Overview

Our qualitative, user-centered approach to digital intervention design has developed an understanding of older adults’ experiences of a technology concept and prototype smartphone app that aims to help them reflect on their well-being, activities, and social connectivity. In this discussion, we consider ways to further research and address these concerns. We also discuss how these findings relate to loneliness and social connectivity research in older adults.

### Principal Findings

Participants highlighted the importance of reflecting on one’s existing social connections as a way of feeling less lonely or more socially connected than they might otherwise feel. This confirms research using similar techniques [28,34]. For older adults (particularly following retirement), family, community, and social groups are important for preventing or overcoming loneliness [54]. Participants in this research found it reassuring to think about staying active participants in their social groups [55]. Positive self-reflection can aid in strengthening social connections based on common interests or bonds, which can reduce loneliness [56]. In addition, mood and activity logging helped them think about what might cause feelings of loneliness, in line with work on the benefits of affect labeling [57], which we suggest may help people with chronic loneliness who have trouble identifying their emotions [11]. These may go some way to address the cognitive and emotional processes involved in chronic loneliness in older adults, including threatened self-efficacy and autonomy in social relations [13].

In addition to reflecting on the number and types of connections they put into their digital social maps, participants also reflected on how they supported and were supported by members of their networks. Mutual reciprocal social support underlies positive social relationships and identities [58,59]. Participants also viewed the increased awareness of the outer circle members positively. These weak ties have a lower frequency of contact, low emotional intensity, and limited intimacy, yet can offer practical support and connections to a wider community [60].

However, for some users, digital social mapping exercises were considered unnecessary. Thus, some older adults may not be interested in using new technologies for *intervention* per se. However, they were able to speculate on the usefulness of the app for others who might experience loneliness. It may also be that if the app had additional features unique to existing technologies, these older adults may have seen more value.

Furthermore, some users were concerned that the app could increase loneliness by increasing awareness of a sparse social map, creating the possibility of a user’s social support needs going unattended, or by provoking feelings of burden to others. This suggests that individuals differ in their feelings regarding the social information they are presented. Research on the self-perpetuating cycle of loneliness suggests that when people become chronically lonely, their negative cognitions and fears about social relationships can prevent them from seeking contact or other activities that interest them [11]. Therefore, we suggest that future work needs to ensure that though reflection on digital social maps is the first step, there is follow-on intervention available, such as suggesting activities people can do that can turn reflection into action [31,42]. Previous research has stated that loneliness interventions could be detrimental to well-being [1,18], but the mechanisms underlying this are not well understood. This study highlighted that it would be irresponsible for reflection tools to contain only self-reflection tools without follow-up action interventions.

On this note, the participants were very interested in the idea of suggested activities and information-sharing features in the future app, and there was interest in the idea of using the app together. Enabling sharing and reciprocity within social groups could lessen older adults’ feelings of overburdening others and tackle age-based stereotypes that older adults are only recipients of support [61]. Nonreciprocal supportive networks can be detrimental to older people’s agency and may distance them from their social circles [62,63]. This reflects older adults’ desire to remain socially active and control their lives, focusing on the positive aspects of aging [33]. The app could therefore be tailored following users’ identity-related interests and needs, implemented using evidence-based intervention techniques (eg, scaffolding [64]) or more adaptive software engineering interventions [65].

These results indicate that the use of UCD methods was successful in supporting the integration of psychology theory into technology even when the technology was being used to probe design concepts rather than to support end users. Although this is a positive indication, significant further work is needed to understand how this relationship between UCD methods and psychology theory can be used to develop technology intended to support end users and to build in the features suggested by our participants [35].

### Limitations

Our sampling strategy succeeded in finding a good distribution of age ranges for people 50+ years old but was unbalanced particularly in terms of ethnicity. This is owing to the makeup of the recruitment panel we used in studies 1 and 3, which is a common issue in older adult research and may reflect systemic issues in the framing and communication of academic research of this type in the United Kingdom. Future research should consider the applicability of such evidence to ethnic minority communities.

We also had very few people in the study who stated that they were lonely. We were keen not to label this as an app just for *lonely older people* because it would create a negative deficit-based focus. Moreover, loneliness is stigmatized; therefore, people often conceal their loneliness [66], making it difficult to explicitly recruit participants experiencing loneliness.

The qualitative analysis focused on participants’ conceptualization or hypothetical use of the app, even in the prototype trial when some functionality was not available. Thus, the participants did not always reflect on their actual experiences of use, which may have changed their views. Therefore, future usability research is still required to improve interface design elements that would make the interfaces more enjoyable to use and engage users longer-term [50,51].

### Conclusions

Smartphone apps have the potential to increase older people’s awareness of their groups and social contacts, which may reduce loneliness and encourage socializing and social support [67]. Our qualitative approach to early app design enabled the inclusion of older adults’ experiences in the technology design and, in doing so, uncovered their needs and concerns should they use an app such as this one.

Older adults most desired functionalities that can support mutual activities, maintain or find new (offline or local) connections, and remain in control of their social lives. Although they were interested in features that facilitated personal reflection on their emotional well-being, they were less interested in sharing their emotional well-being, as it can lead to concerns of overburdening others. We suggest that future iterations of the app could provide a novel private social networking function by facilitating social support relationships and activity groups—a function not currently tailored to on many current social media platforms. This may also appeal to participants for whom the app did not appear to provide much value above their existing digital interactions.

Moreover, designers of digital solutions need to be careful that they do not increase loneliness by making the user aware of a lack of connections or support in their social network, unless they can offer specific means of addressing those needs. In future iterations of this app or similar studies, there needs to be a focus on how to inoculate socially vulnerable individuals from any accentuation of their loneliness. For example, the app could add a means of positive reappraisal of limited social connectedness by referencing valued social concepts such as independence and self-sufficiency or by helping people set positive goals [28].

Our study illustrates the continued importance of UCD work to ensure that digital interventions are designed for newly digitally literate populations such as older adults.

## Appendix

Multimedia Appendix 1 Study 1 concept video and questions.

Multimedia Appendix 2 Study 2 wireframes.

Multimedia Appendix 3 Study 2 interview schedule.

Multimedia Appendix 4 Study 3 interview schedule.

Multimedia Appendix 5 Study 3 prototype screenshots.

## Abbreviations

UCD: user-centered design

## Data Availability

The qualitative study transcripts may contain confidential information and are stored privately in The Open University’s figshare repository; these can be shared on reasonable request to the corresponding author. The technology probes and screen captures of the application are available on internet [68].
