# Supplementary material for: Digital Intervention in Loneliness in Older Adults: Qualitative Analysis of User Studies
Source: JMIR Form Res. 2023 Jan 27;7:e42172. doi: 10.2196/42172 (PMC9919429; doi:10.2196/42172)
Supplement: Multimedia Appendix 2 [file formative_v7i1e42172_app2.pdf]

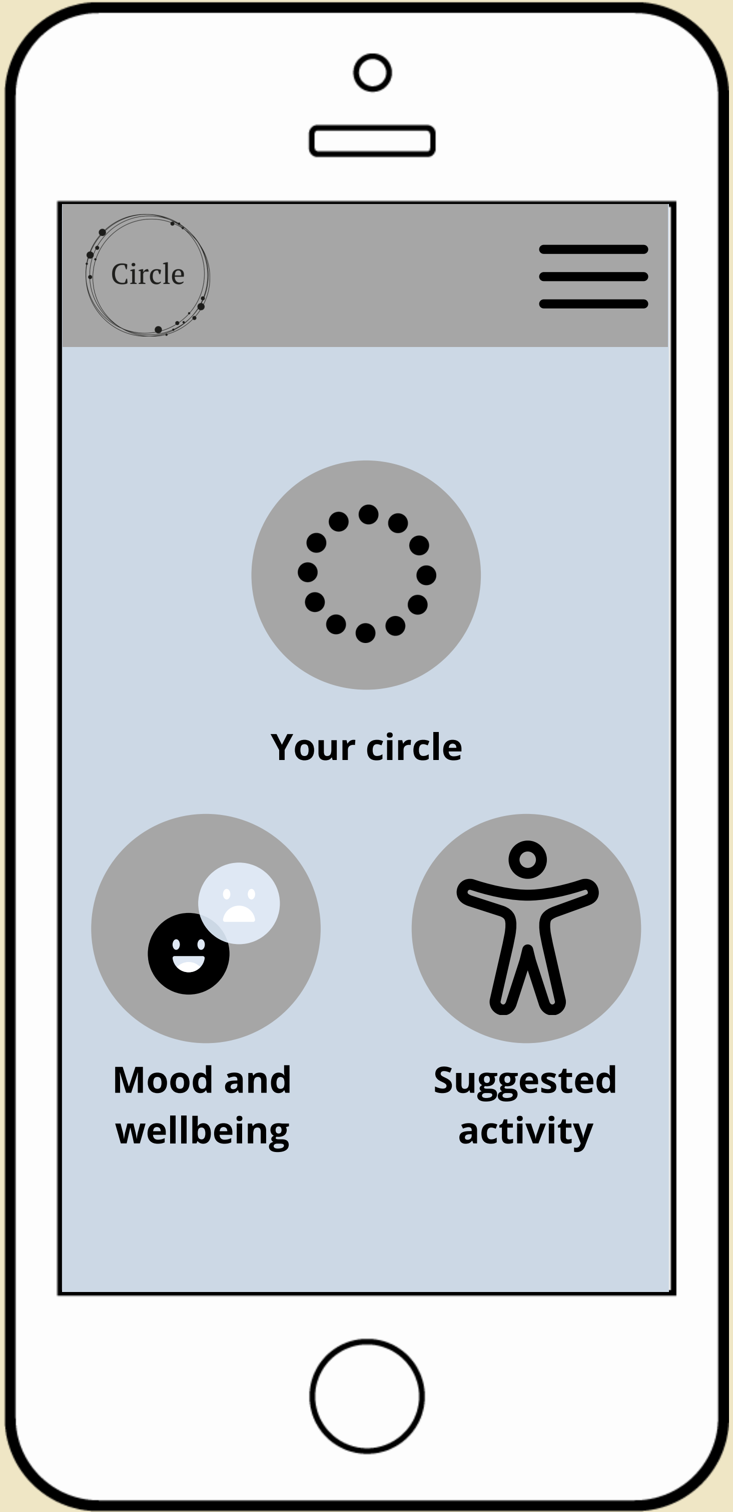
Circle
Your circle
Mood and wellbeing
Suggested activity

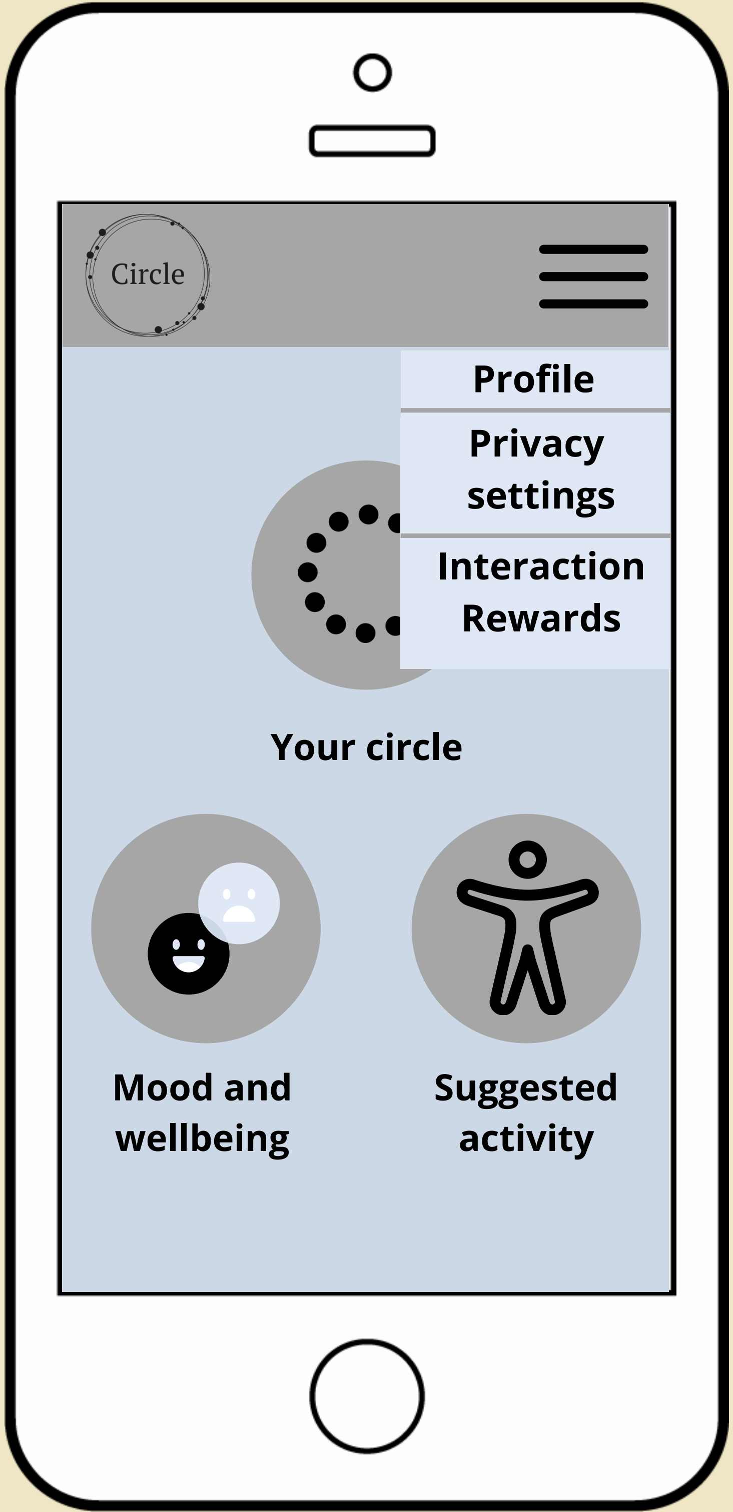
Circle
Profile
Privacy settings
Interaction Rewards
Your circle
Mood and wellbeing
Suggested activity

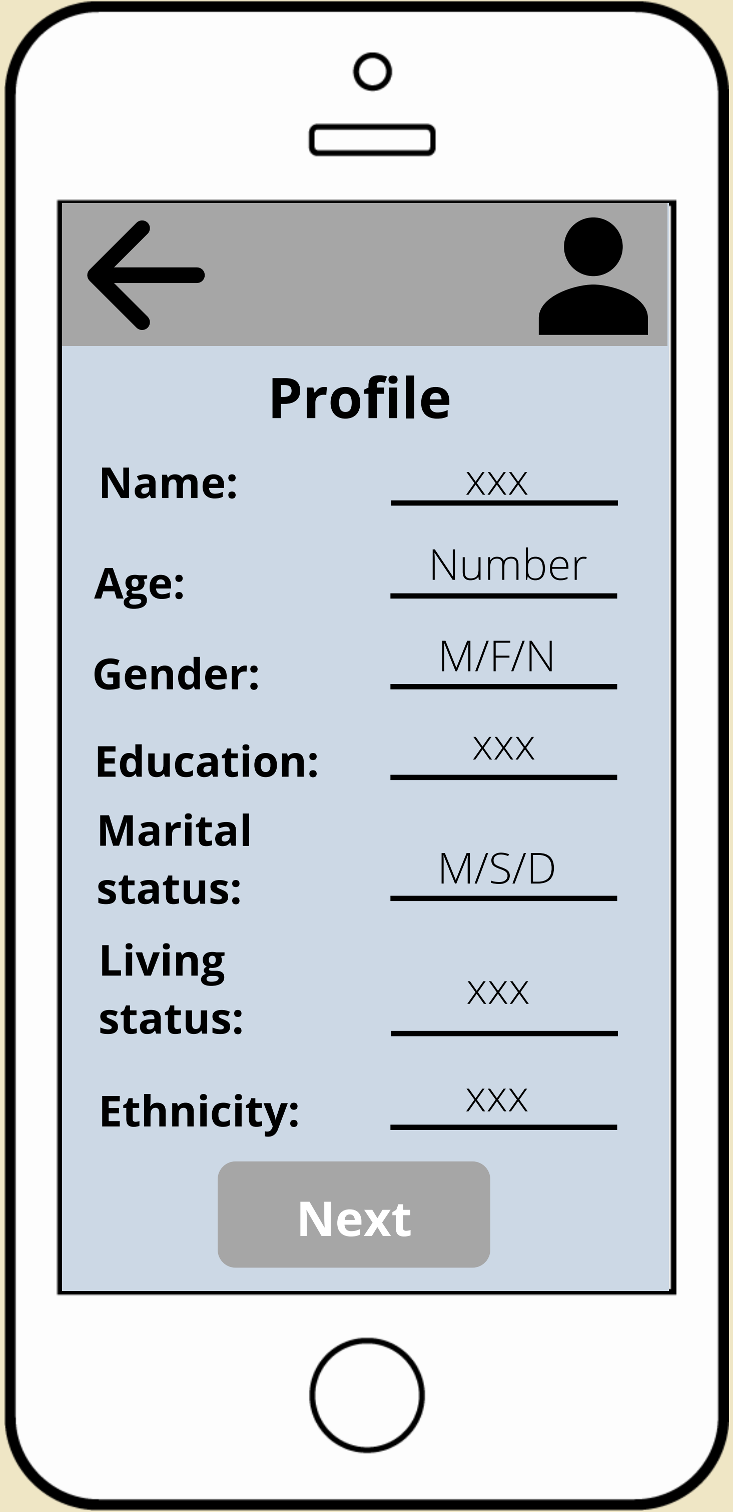
Profile
Name:
XXX
Age:
Number
Gender:
M/F/N
Education:
XXX
Marital status:
M/S/D
Living status:
XXX
Ethnicity:
XXX
Next

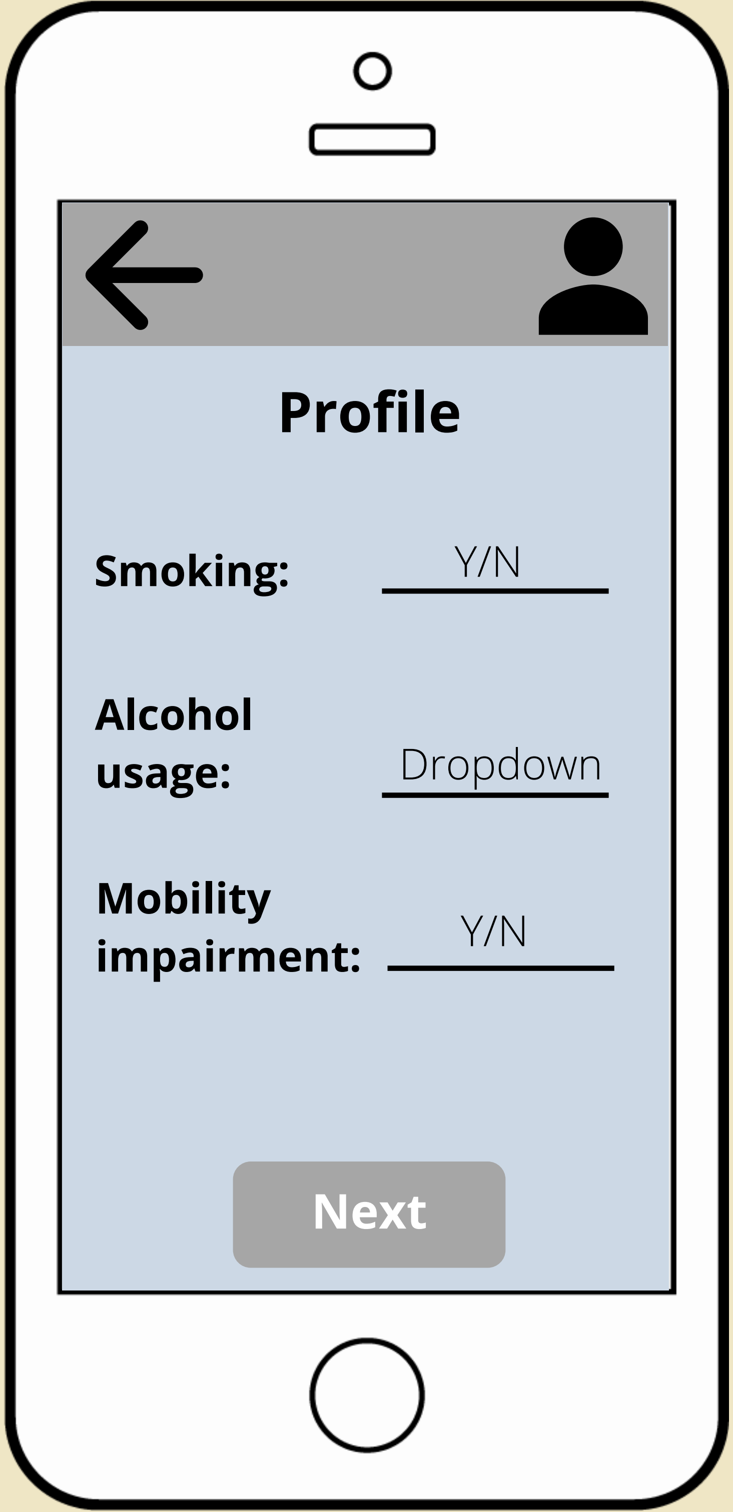
Profile
Smoking:
Y/N
Alcohol usage:
Dropdown
Mobility impairment:
Y/N
Next

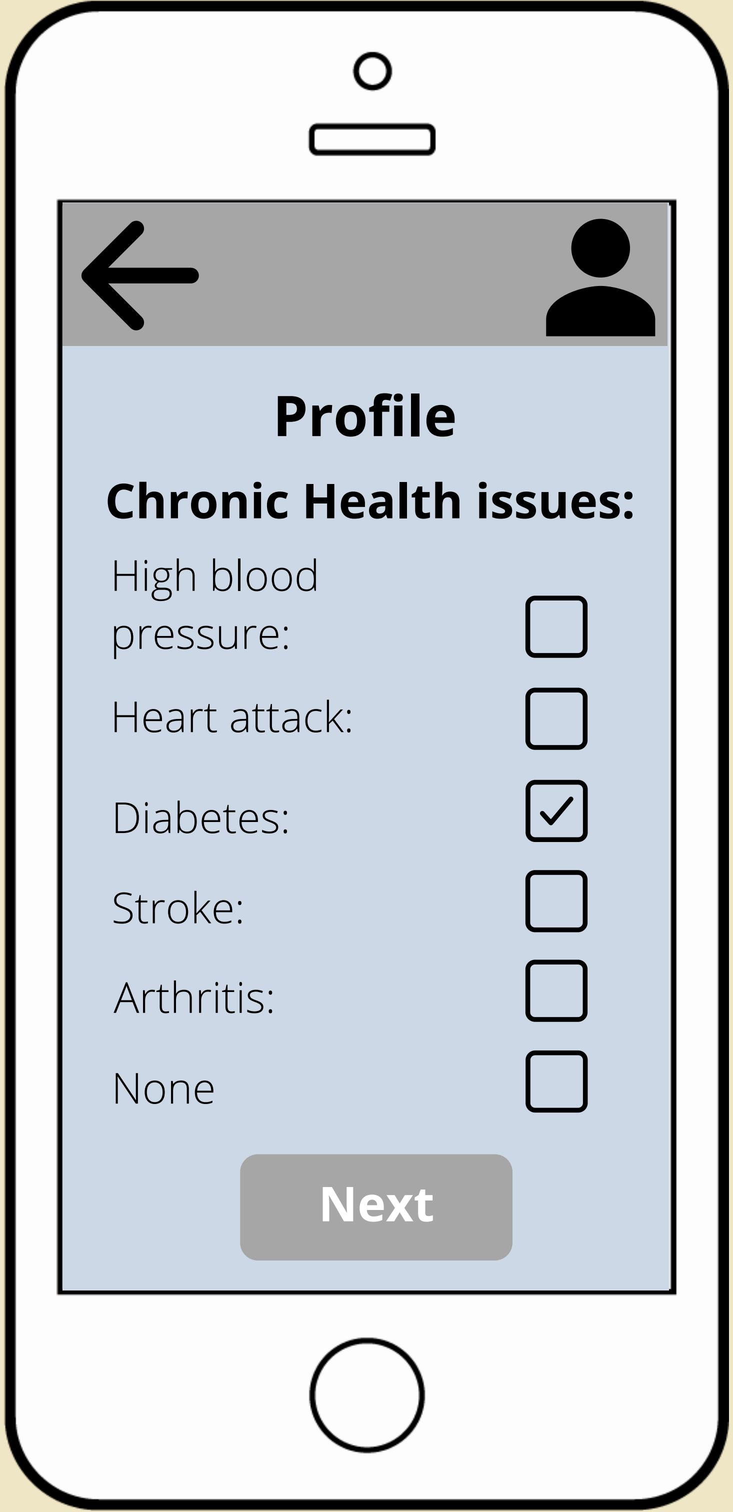
Profile
Chronic Health issues:
High blood pressure:
Heart attack:
Diabetes:
Stroke:
Arthritis:
None
Next

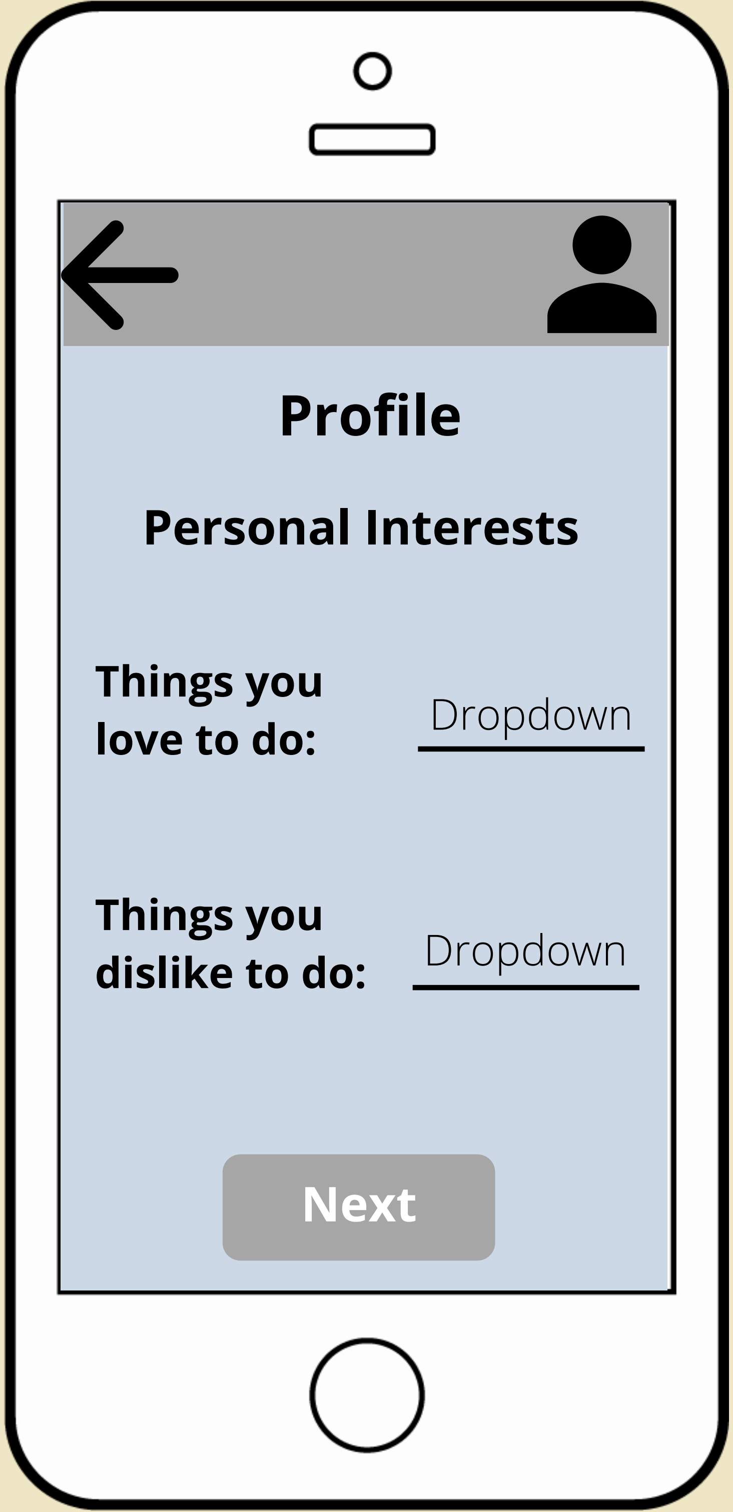
Profile
Personal Interests
Things you love to do:
Dropdown
Things you dislike to do:
Dropdown
Next

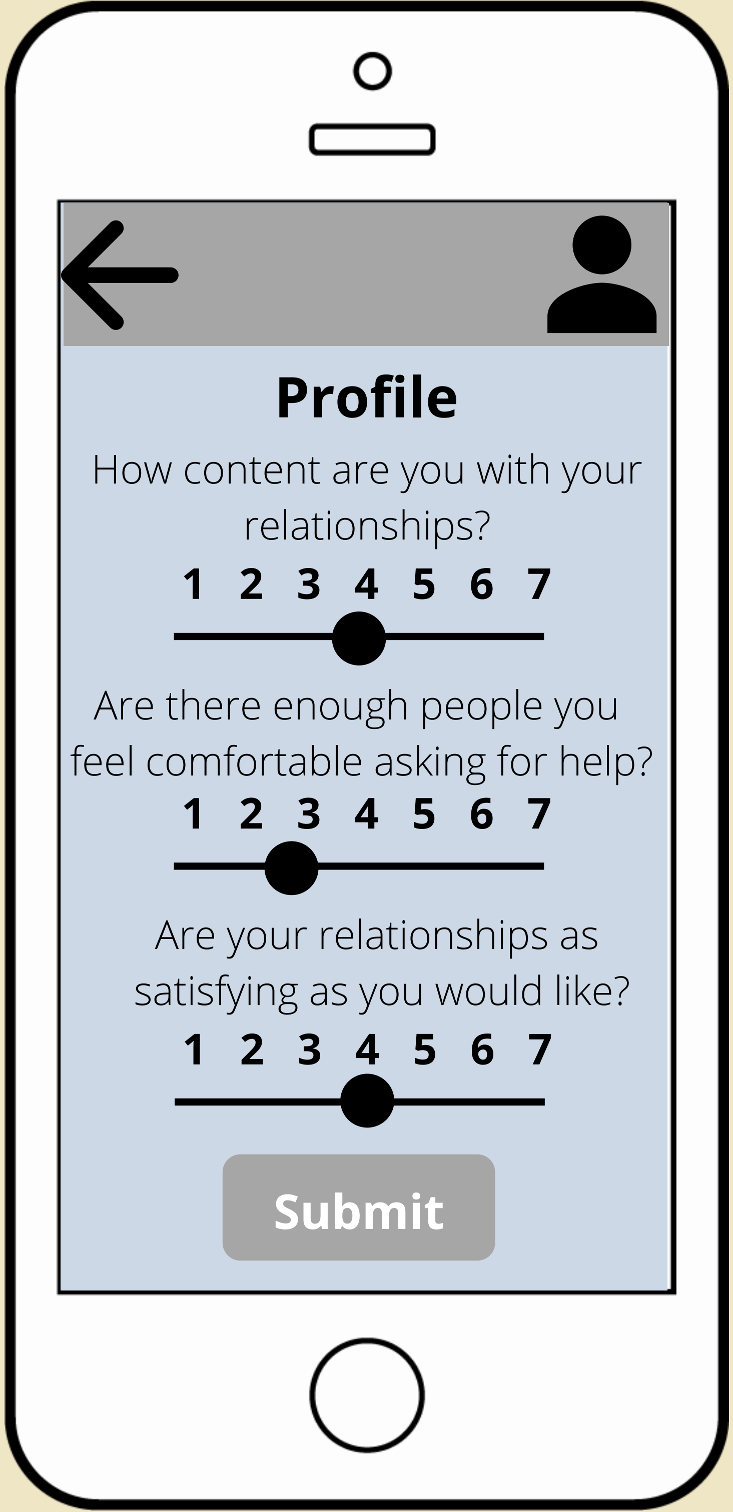
Profile
How content are you with your relationships?
1 2 3 4 5 6 7
Are there enough people you feel comfortable asking for help?
1 2 3 4 5 6 7
Are your relationships as satisfying as you would like?
1 2 3 4 5 6 7
Submit

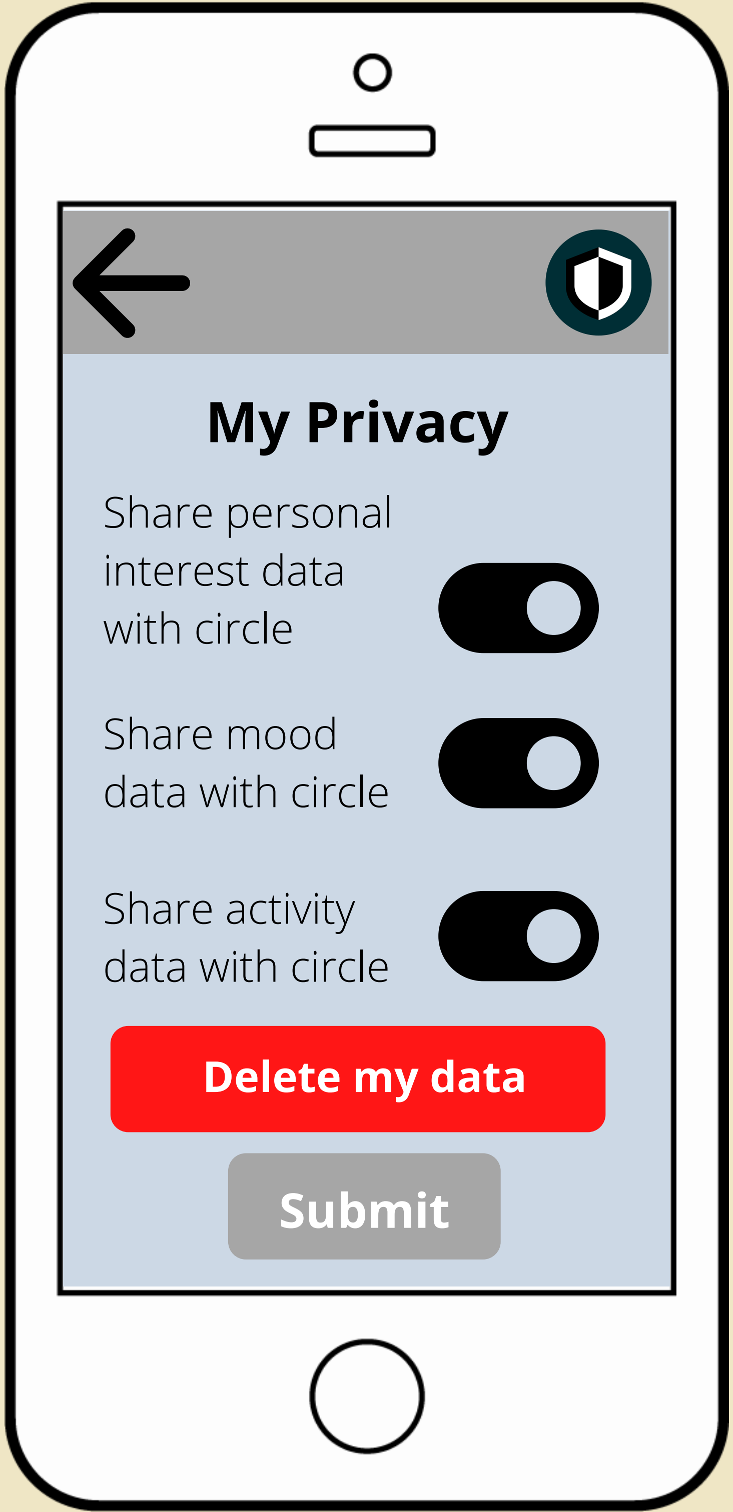
My Privacy
Share personal interest data with circle
Share mood data with circle
Share activity data with circle
Delete my data
Submit

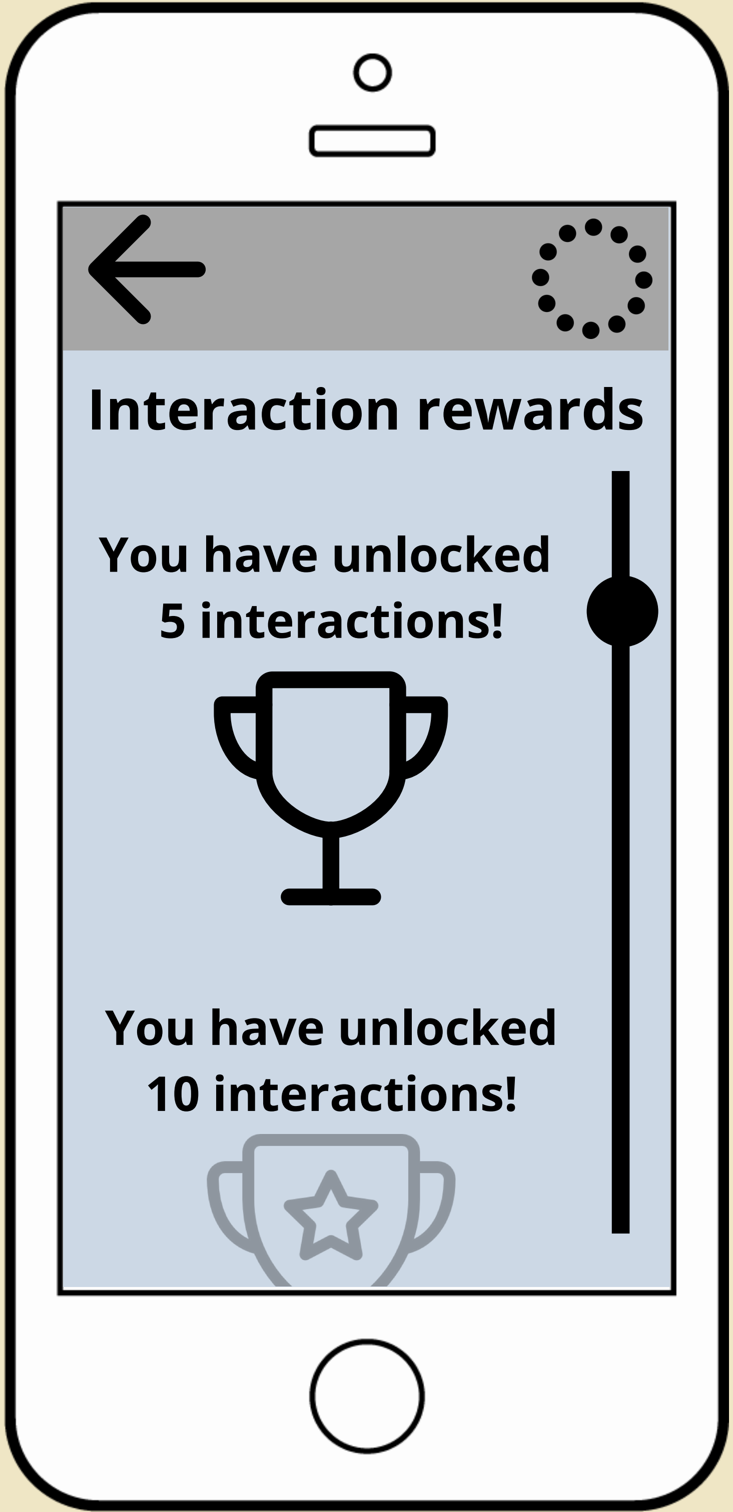
Interaction rewards
You have unlocked
5 interactions!
You have unlocked
10 interactions!

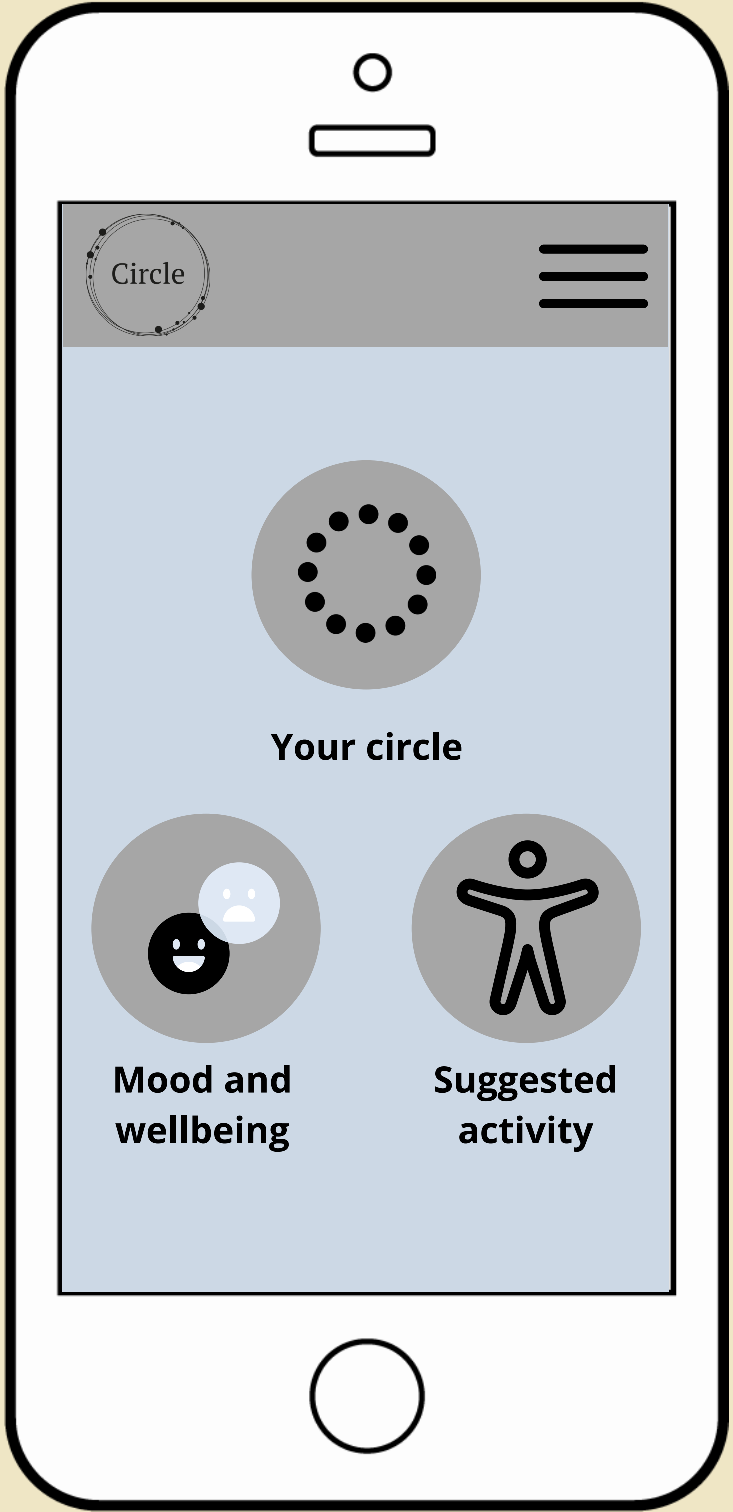
Circle
Your circle
Mood and wellbeing
Suggested activity

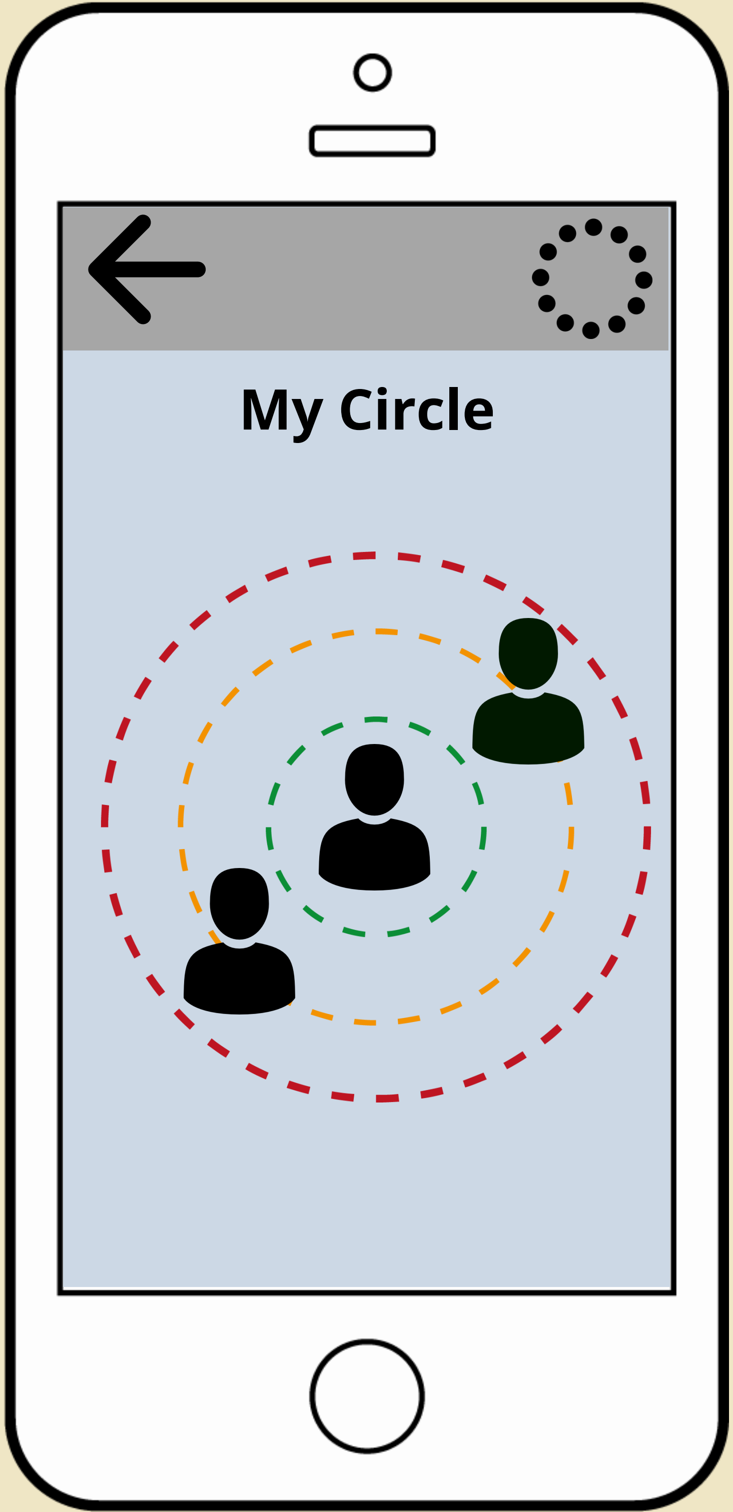
My Circle

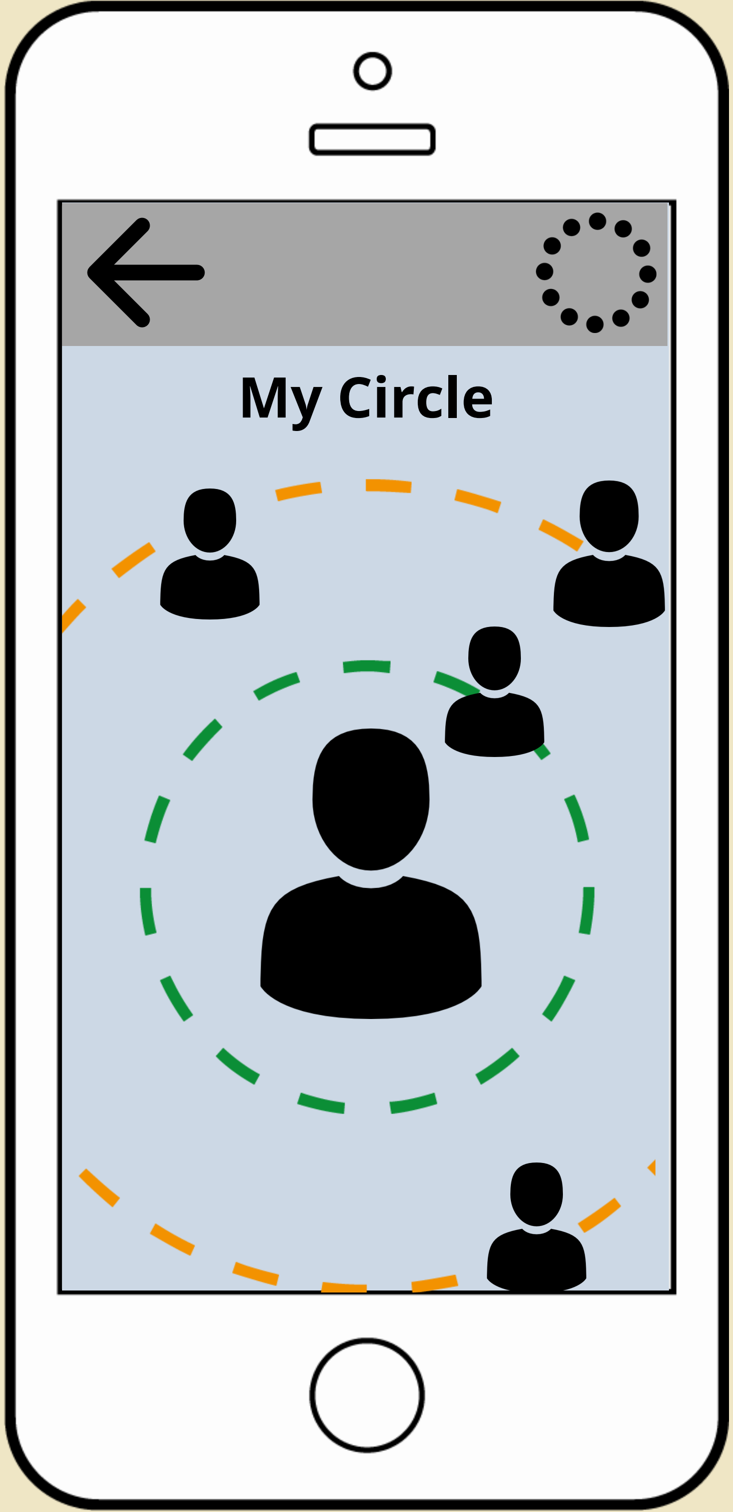
My Circle

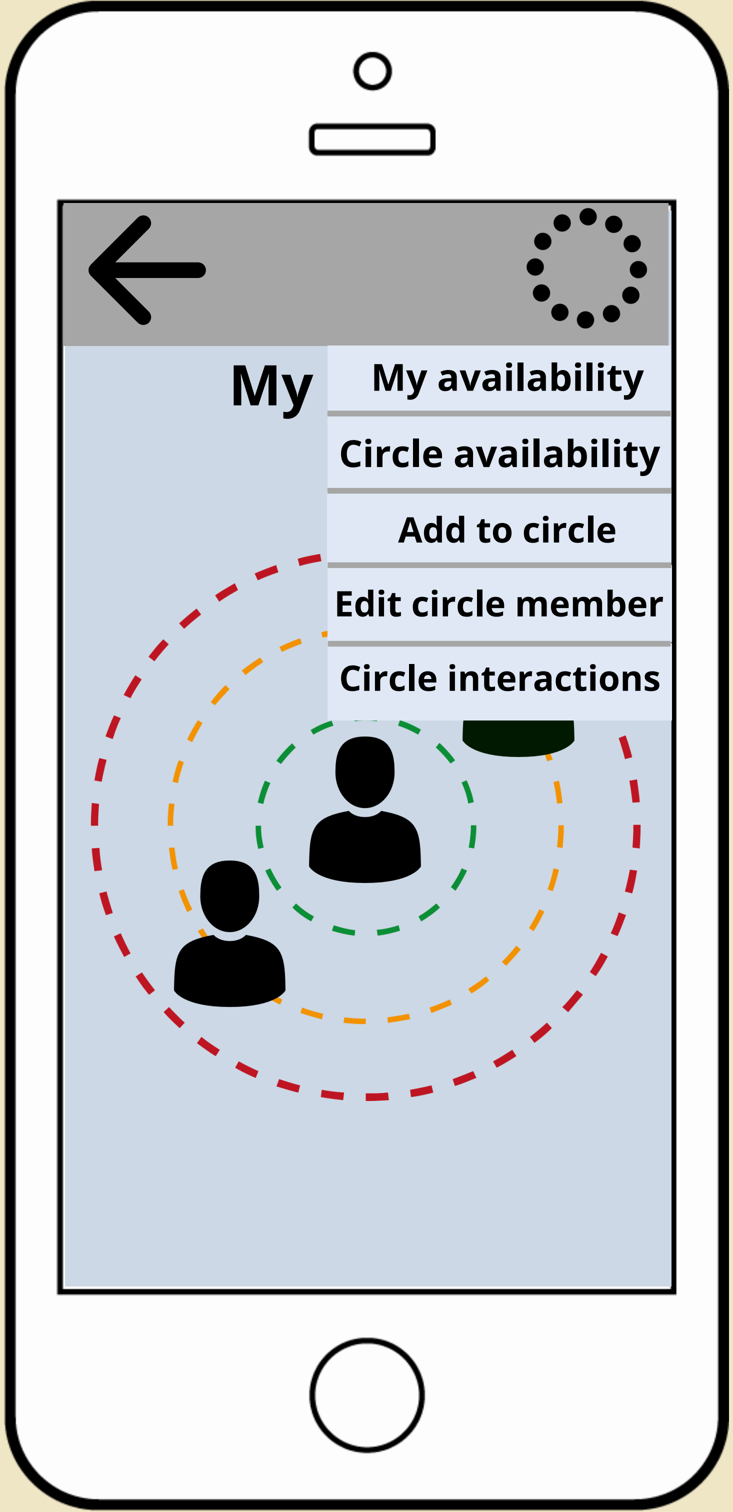
My
My availability
Circle availability
Add to circle
Edit circle member
Circle interactions

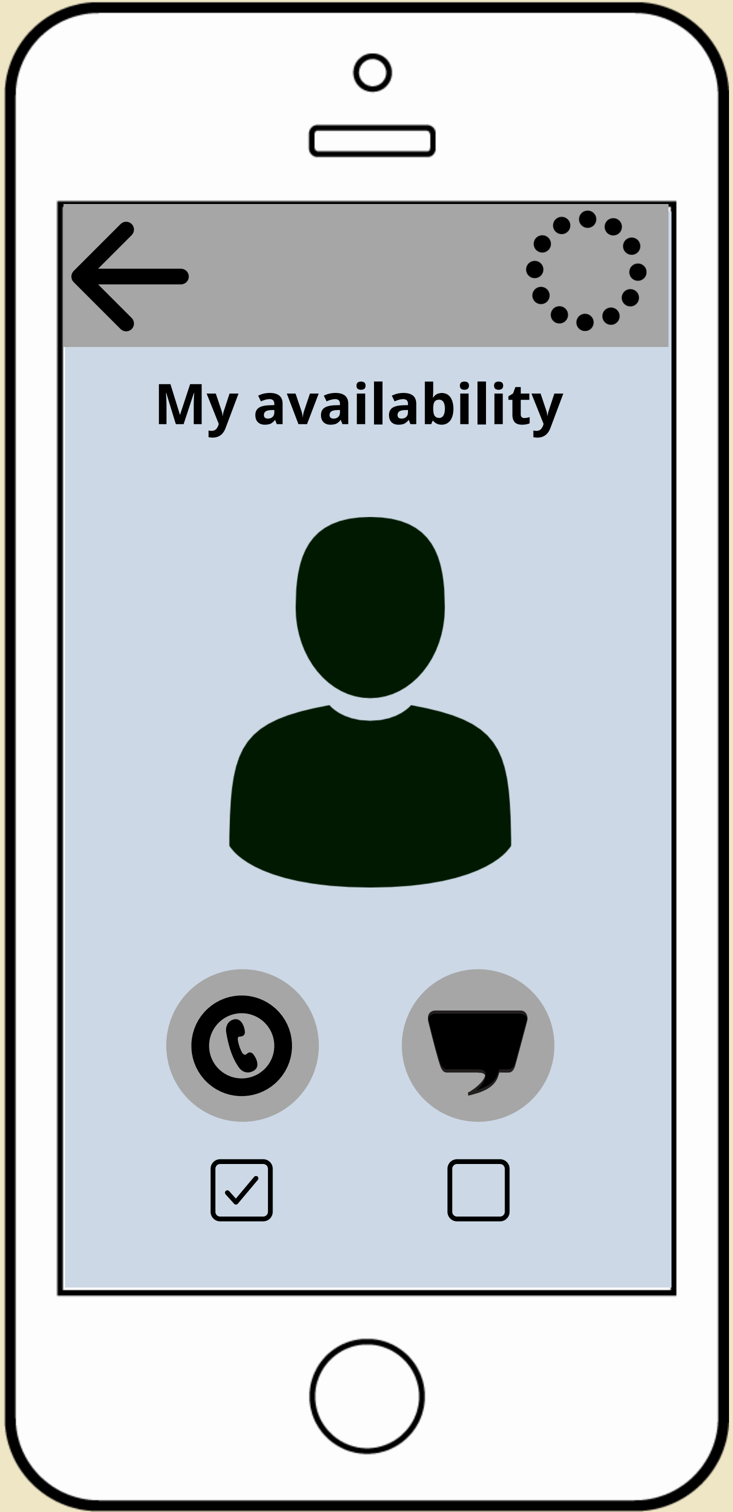
My availability

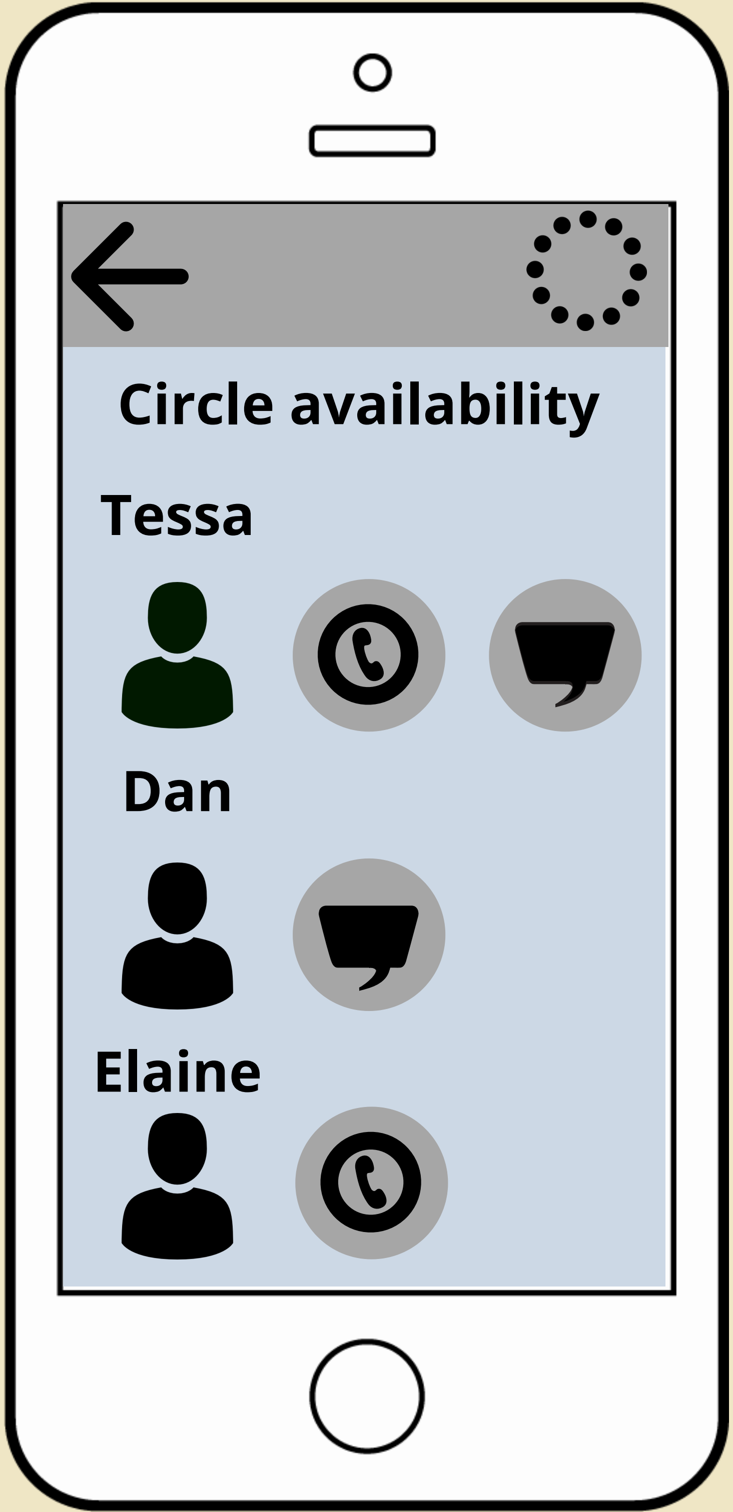
Circle availability
Tessa
Dan
Elaine

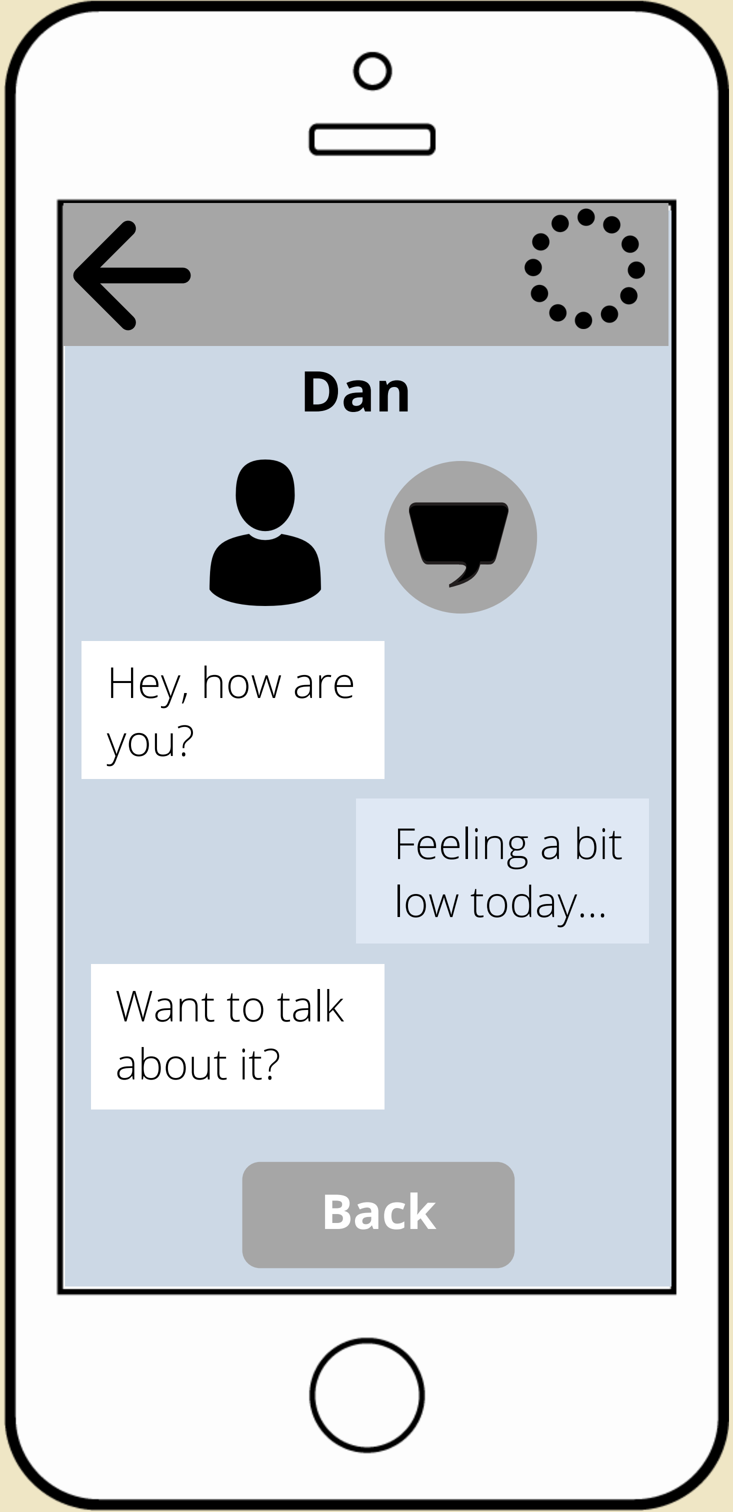
Dan
Hey, how are you?
Feeling a bit low today...
Want to talk about it?
Back

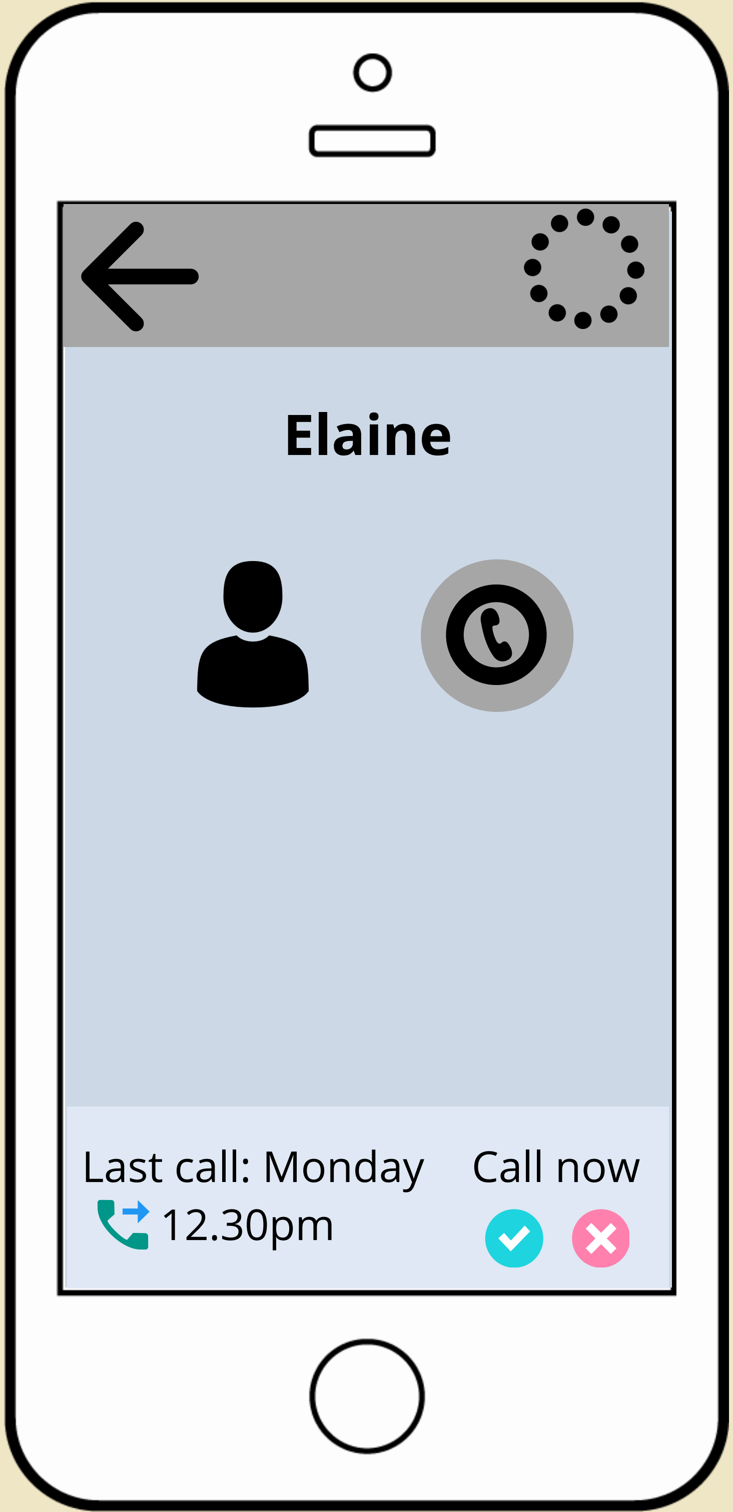
Elaine
Last call: Monday
12.30pm
Call now

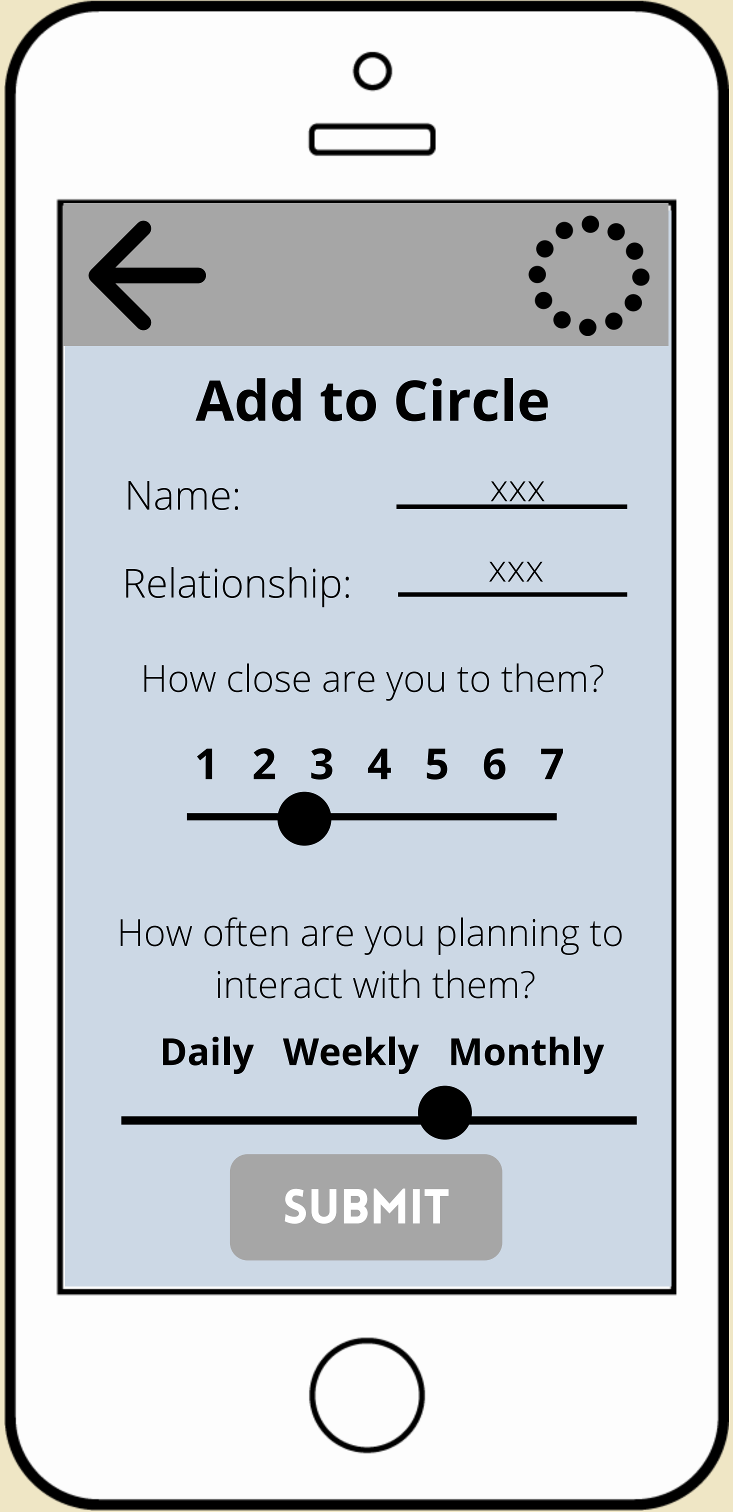
Add to Circle
Name:
XXX
Relationship:
XXX
How close are you to them?
1 2 3 4 5 6 7
How often are you planning to interact with them?
Daily Weekly Monthly
SUBMIT

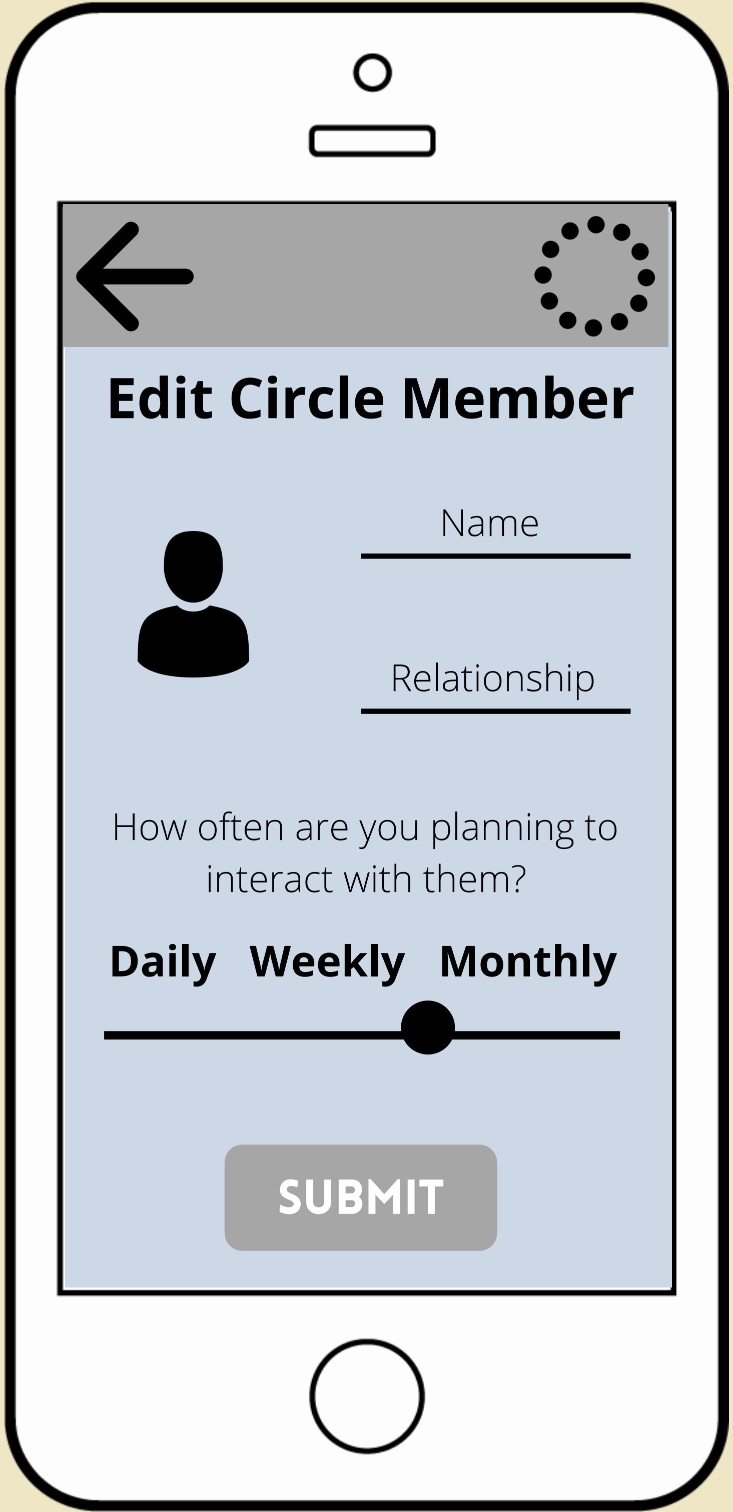
Edit Circle Member
Name
Relationship
How often are you planning to interact with them?
Daily
Weekly
Monthly
SUBMIT

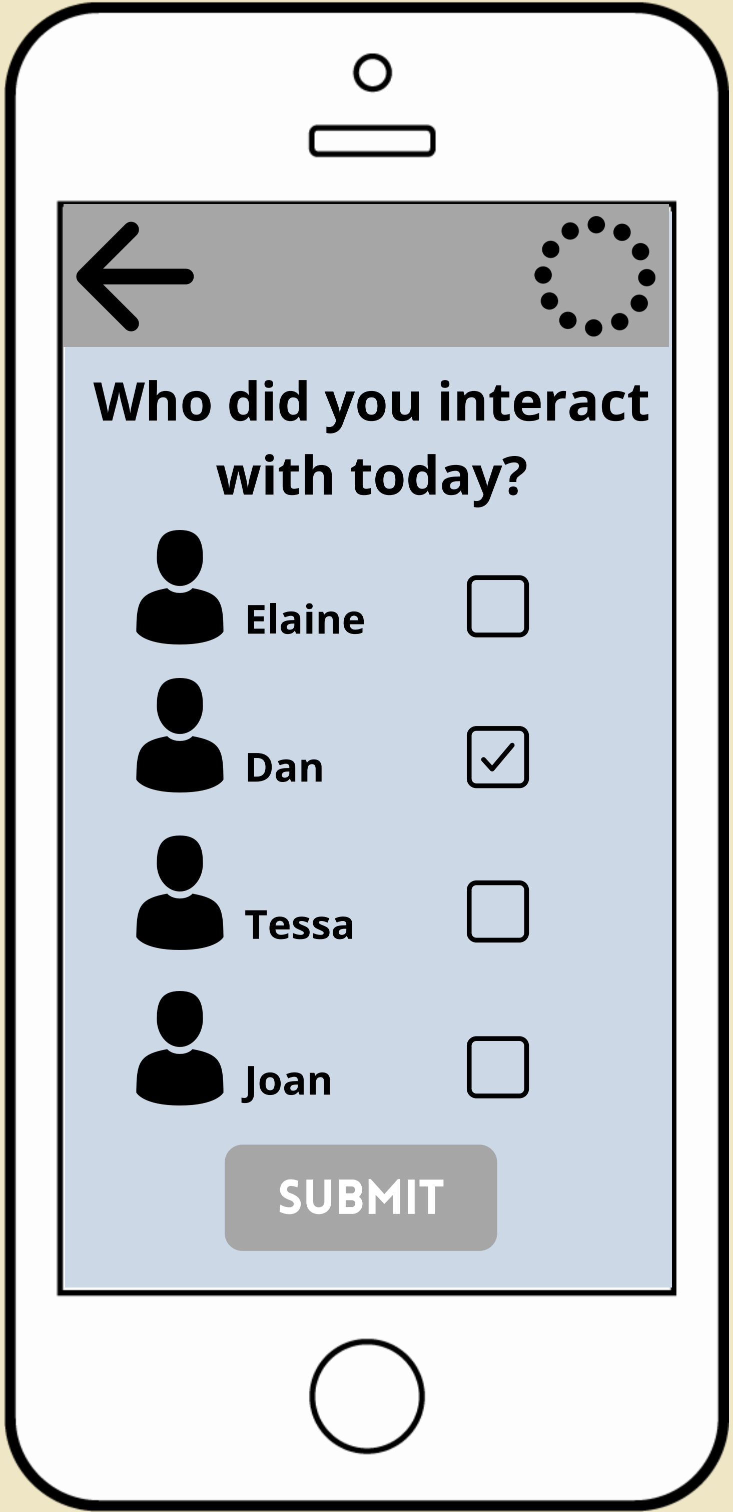
Who did you interact with today?
Elaine
Dan
Tessa
Joan
SUBMIT

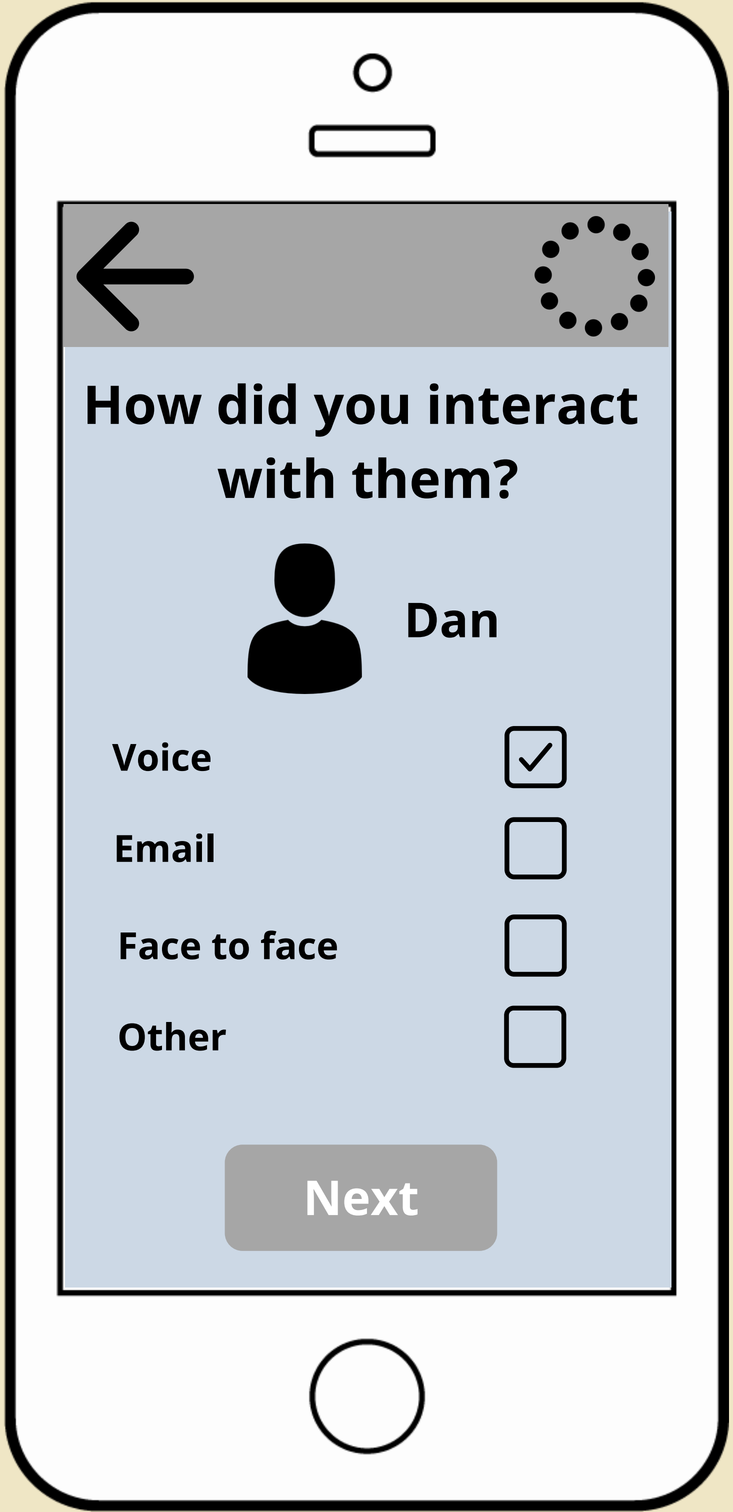
How did you interact with them?
Dan
Voice
Email
Face to face
Other
Next

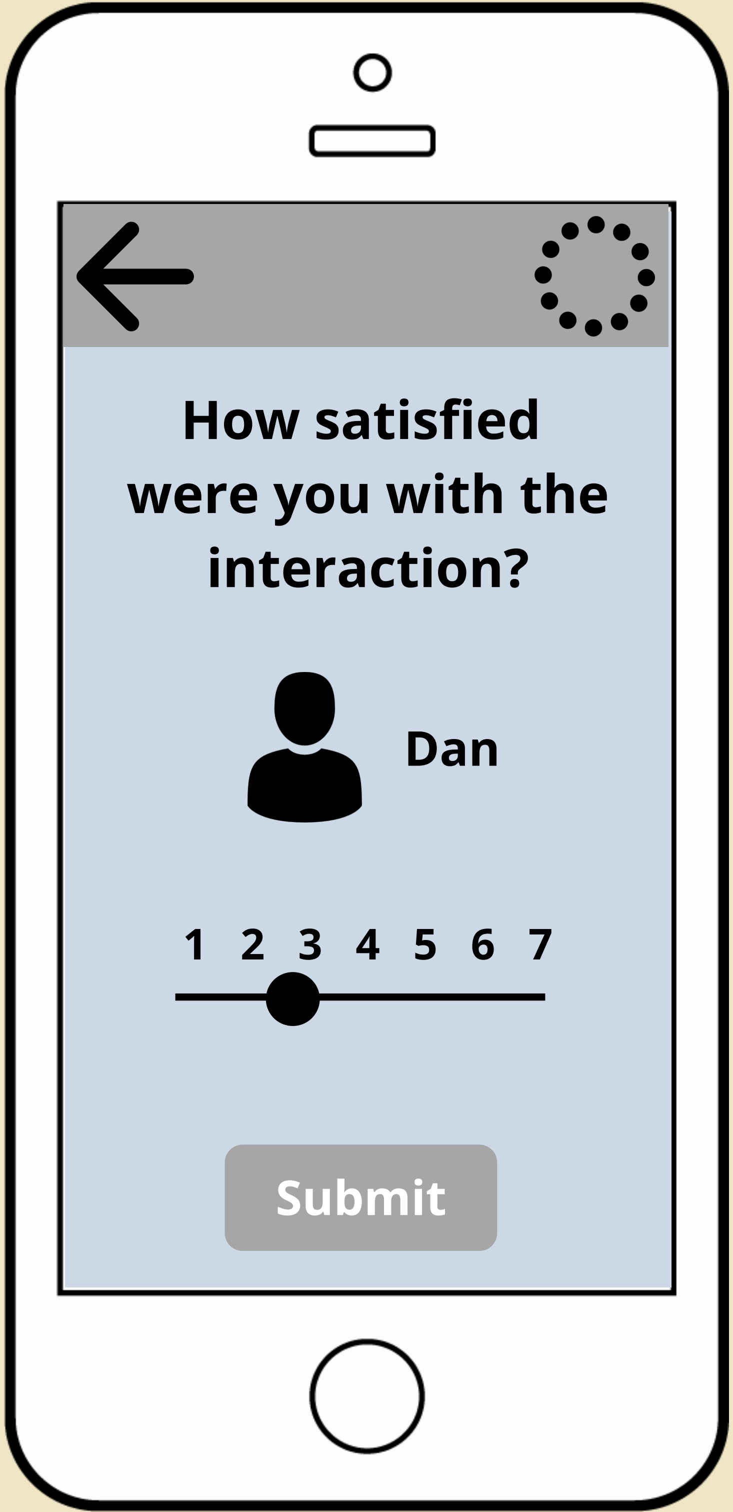
How satisfied were you with the interaction?
Dan
1 2 3 4 5 6 7
Submit

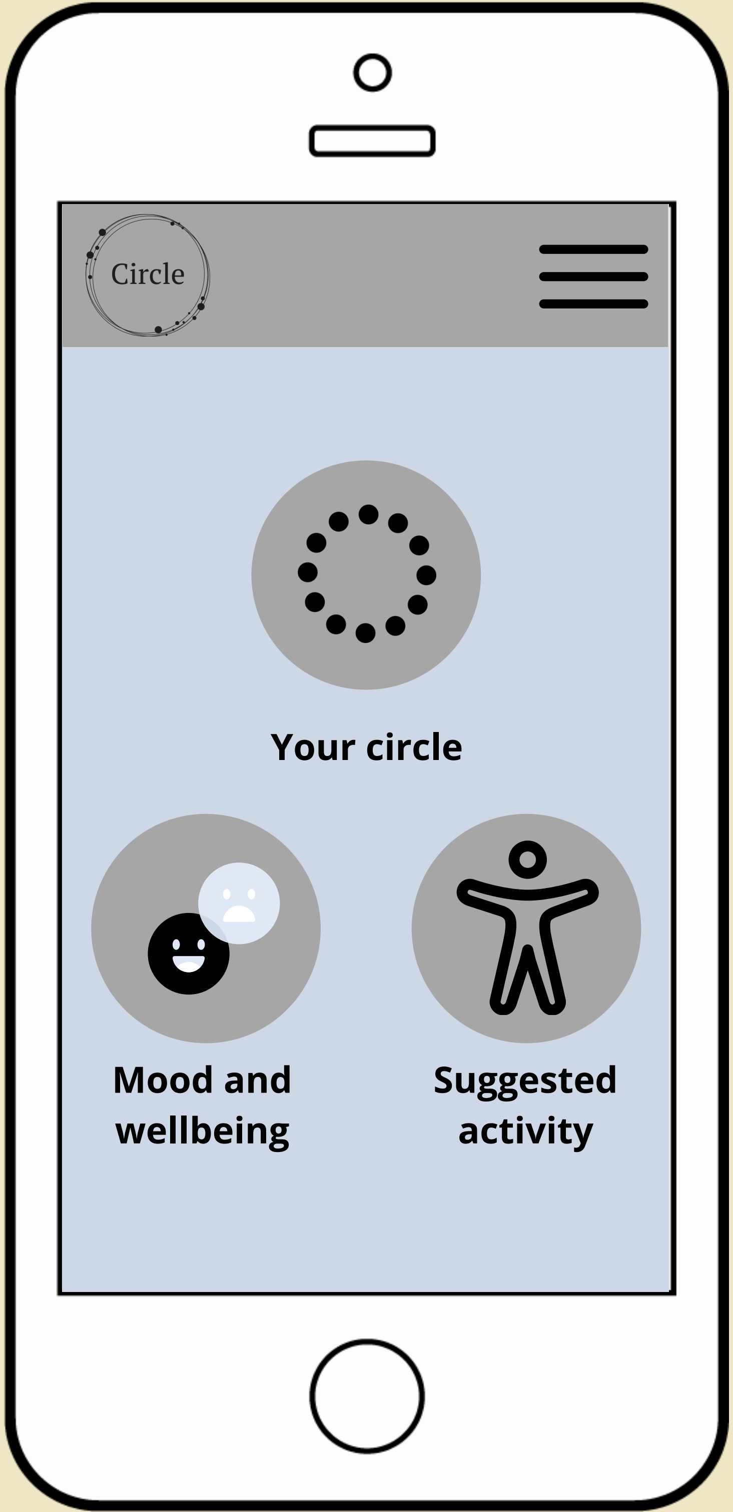
Circle
Your circle
Mood and wellbeing
Suggested activity

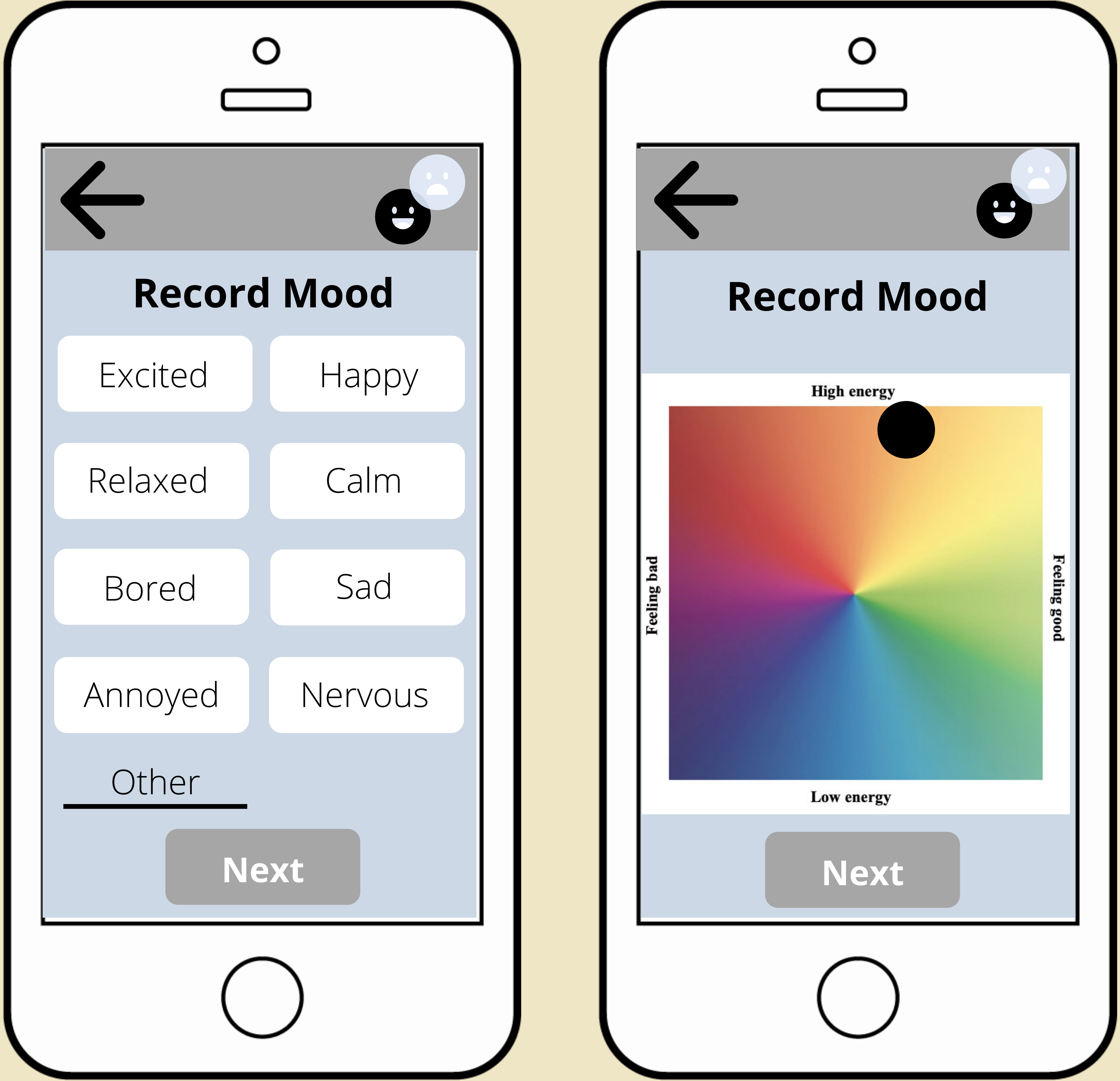
Record Mood
Excited
Happy
Relaxed
Calm
Bored
Sad
Annoyed
Nervous
Other
Next
Record Mood
High energy
Feeling bad
Feeling good
Low energy
Next

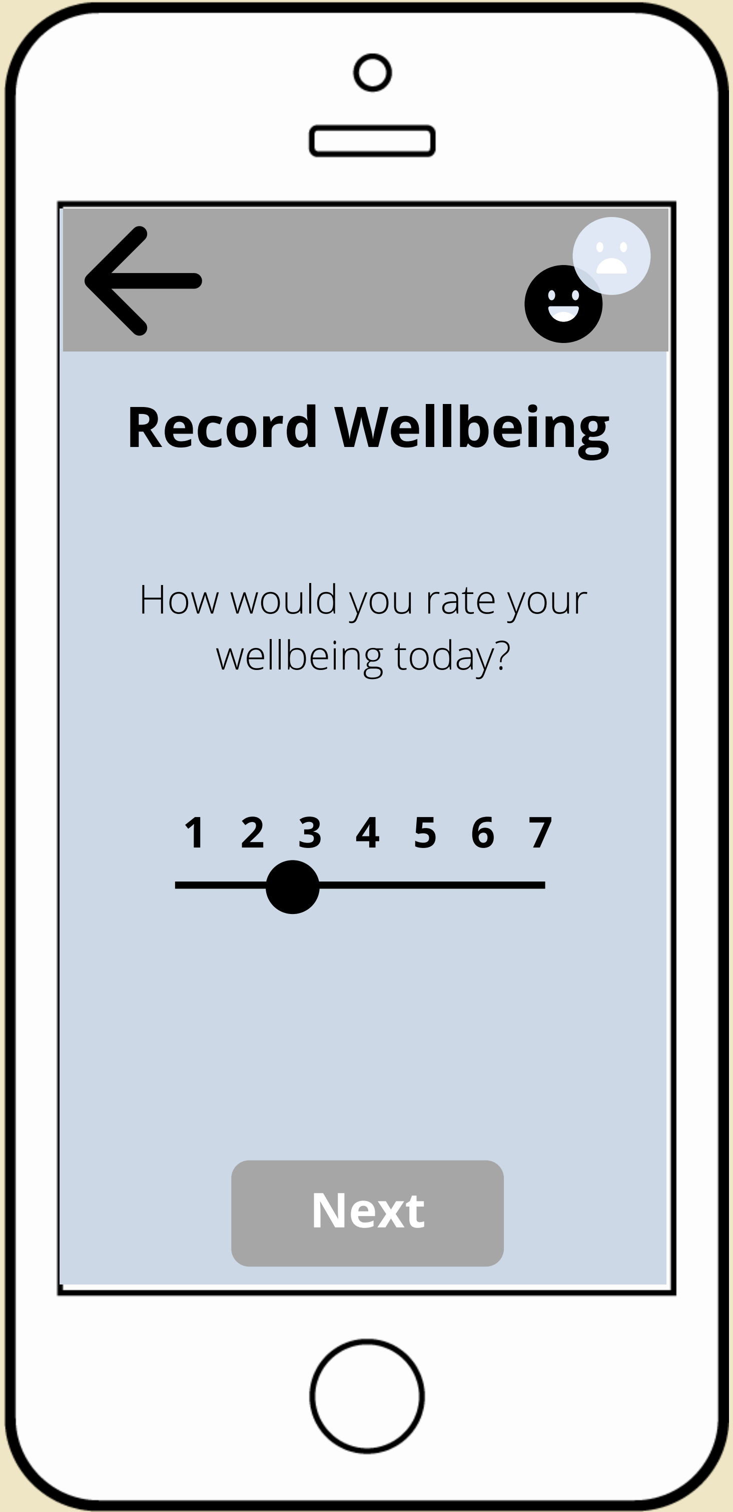
Record Wellbeing
How would you rate your wellbeing today?
1 2 3 4 5 6 7
Next

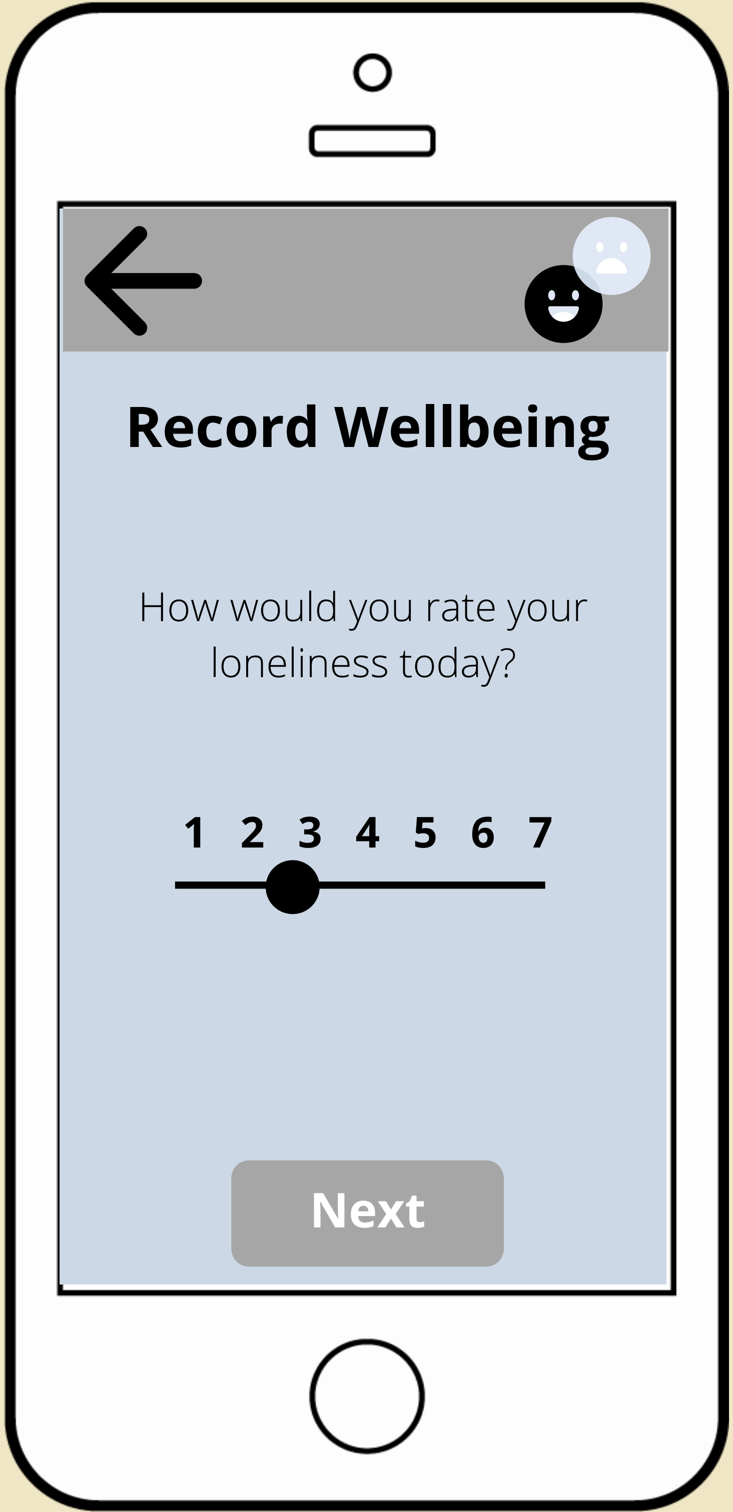
Record Wellbeing
How would you rate your loneliness today?
1 2 3 4 5 6 7
Next

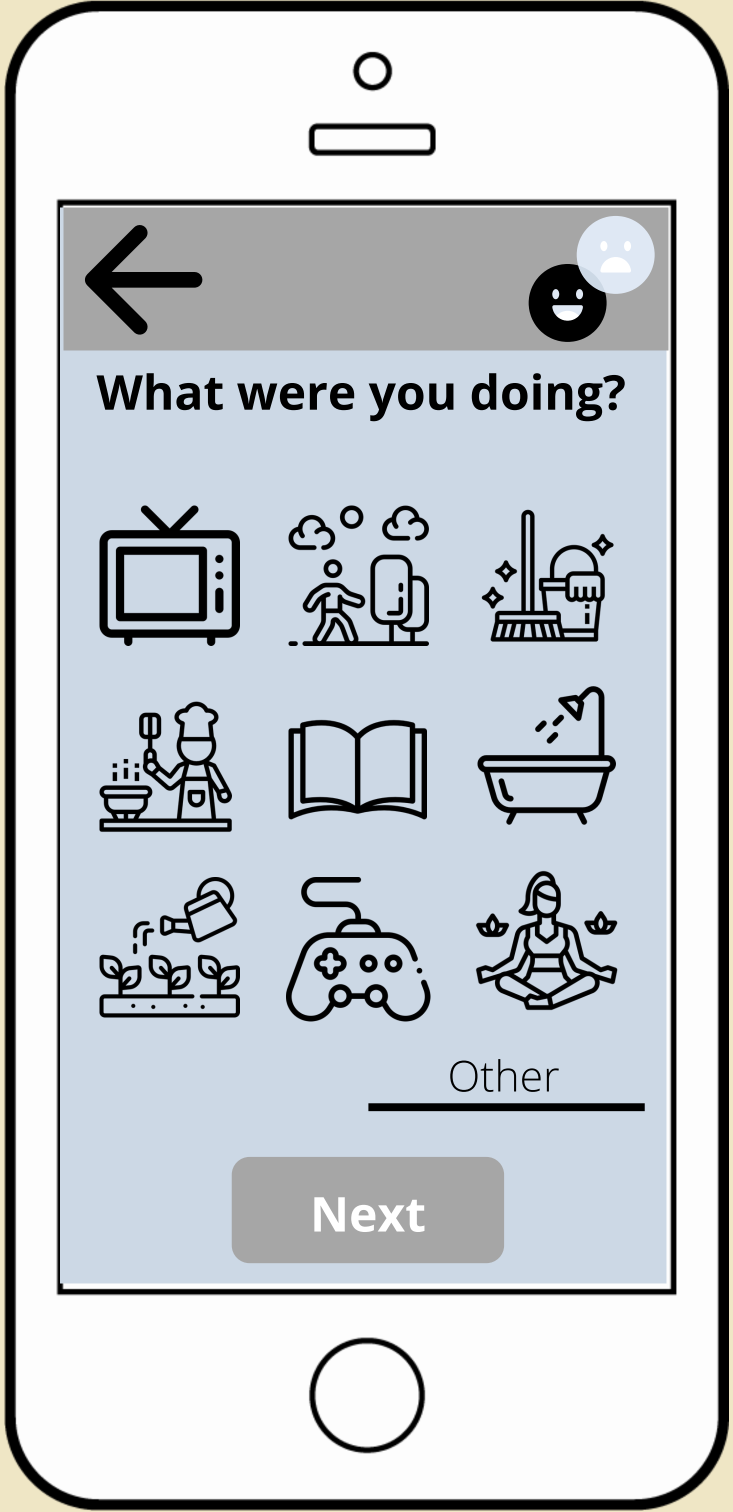
What were you doing?
Other
Next

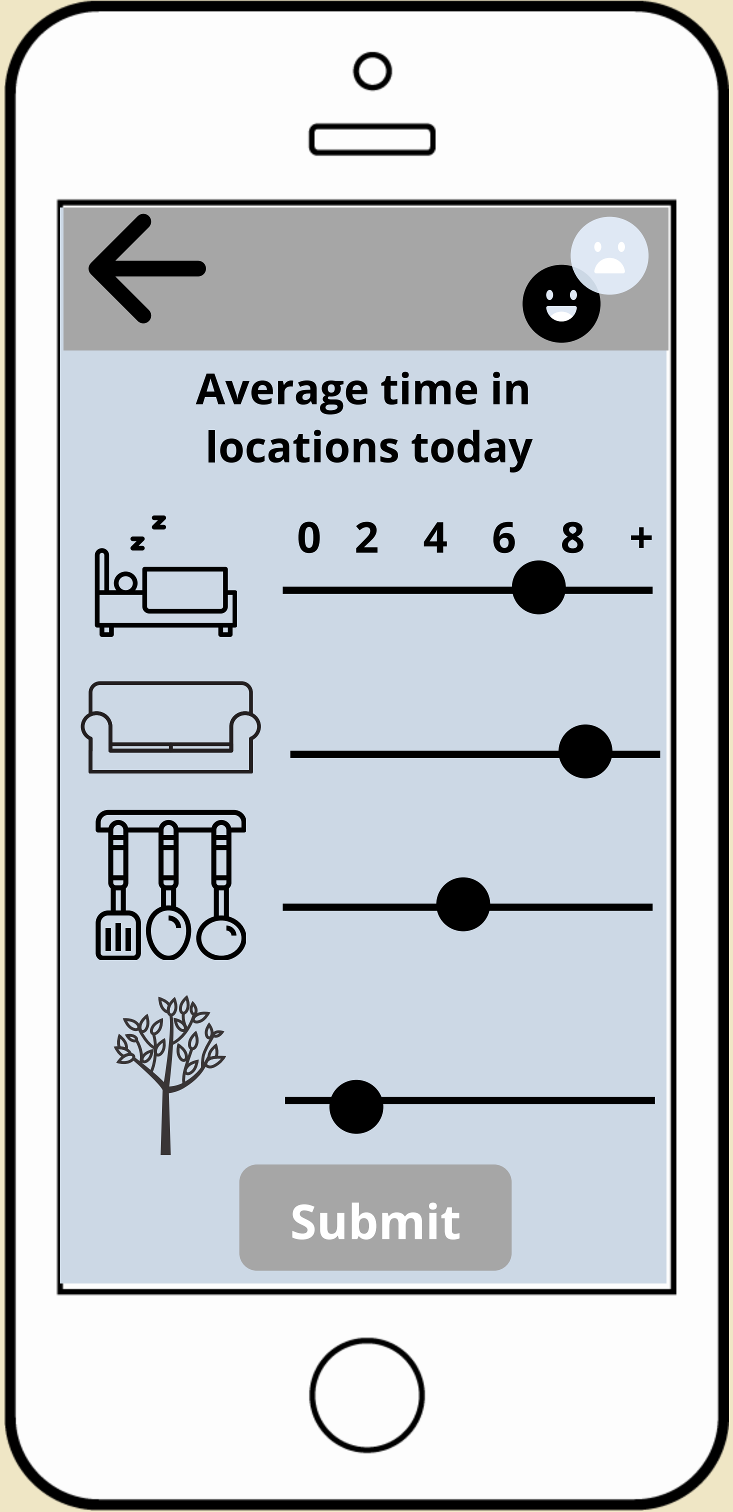
Average time in
locations today
0 2 4 6 8 +
Submit

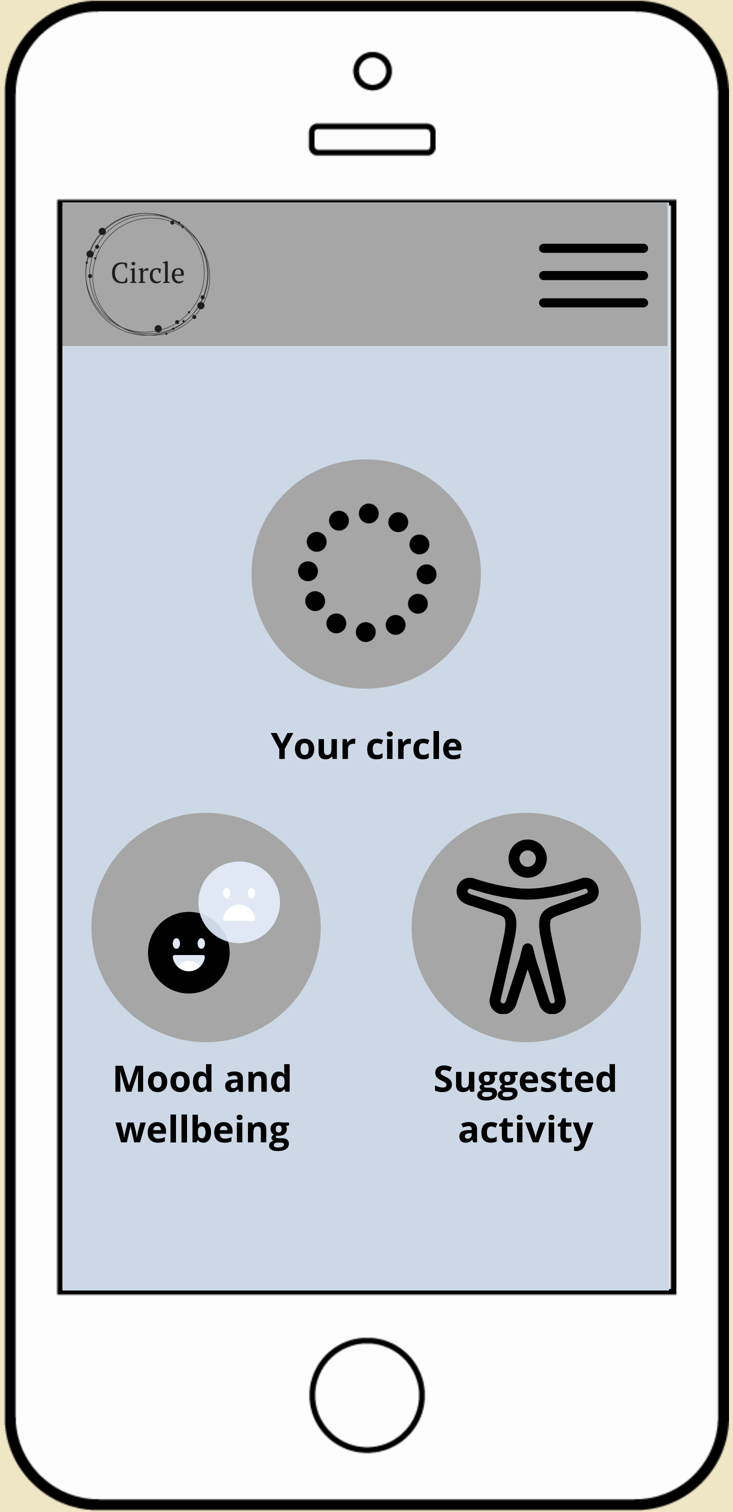
Circle
Your circle
Mood and wellbeing
Suggested activity

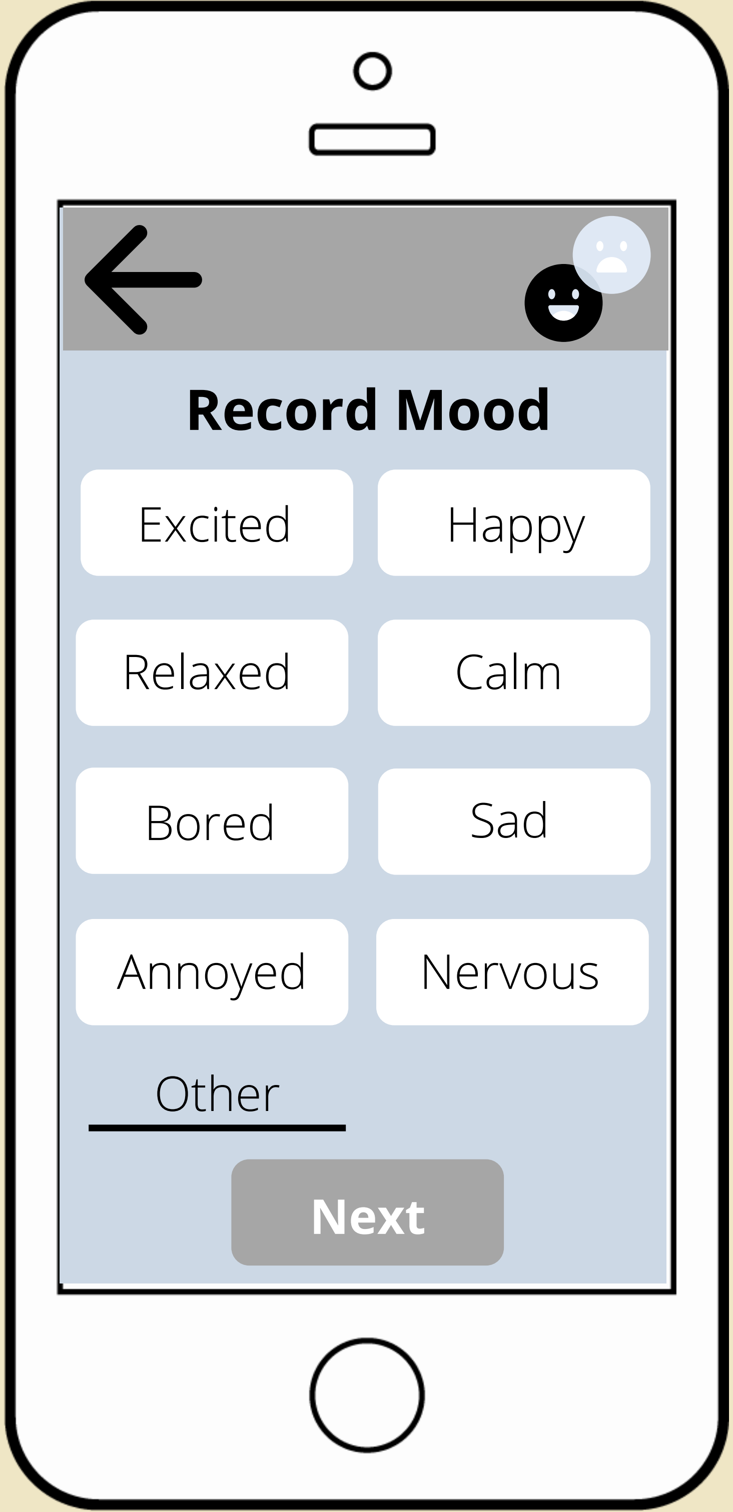
Record Mood
Excited
Happy
Relaxed
Calm
Bored
Sad
Annoyed
Nervous
Other
Next

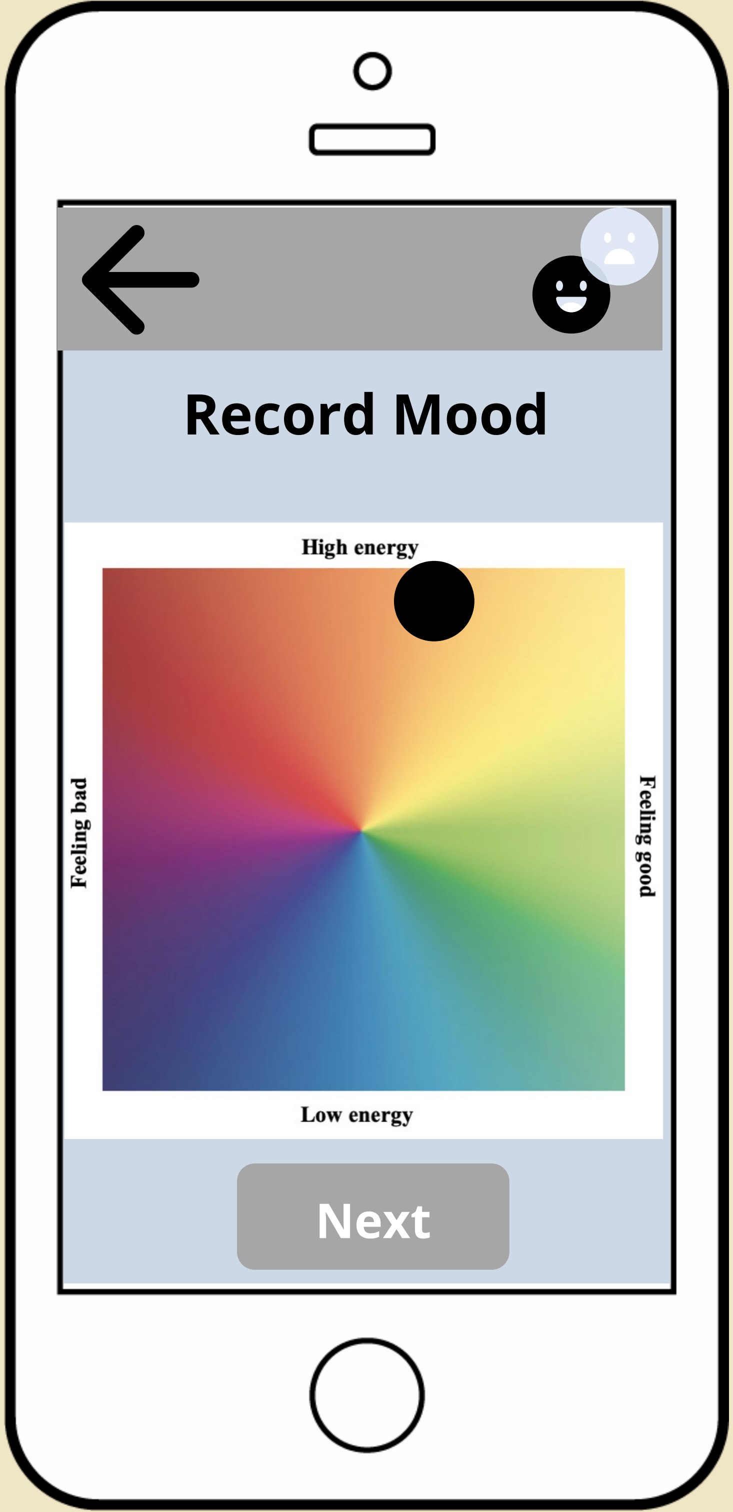
Record Mood
High energy
Feeling bad
Feeling good
Low energy
Next

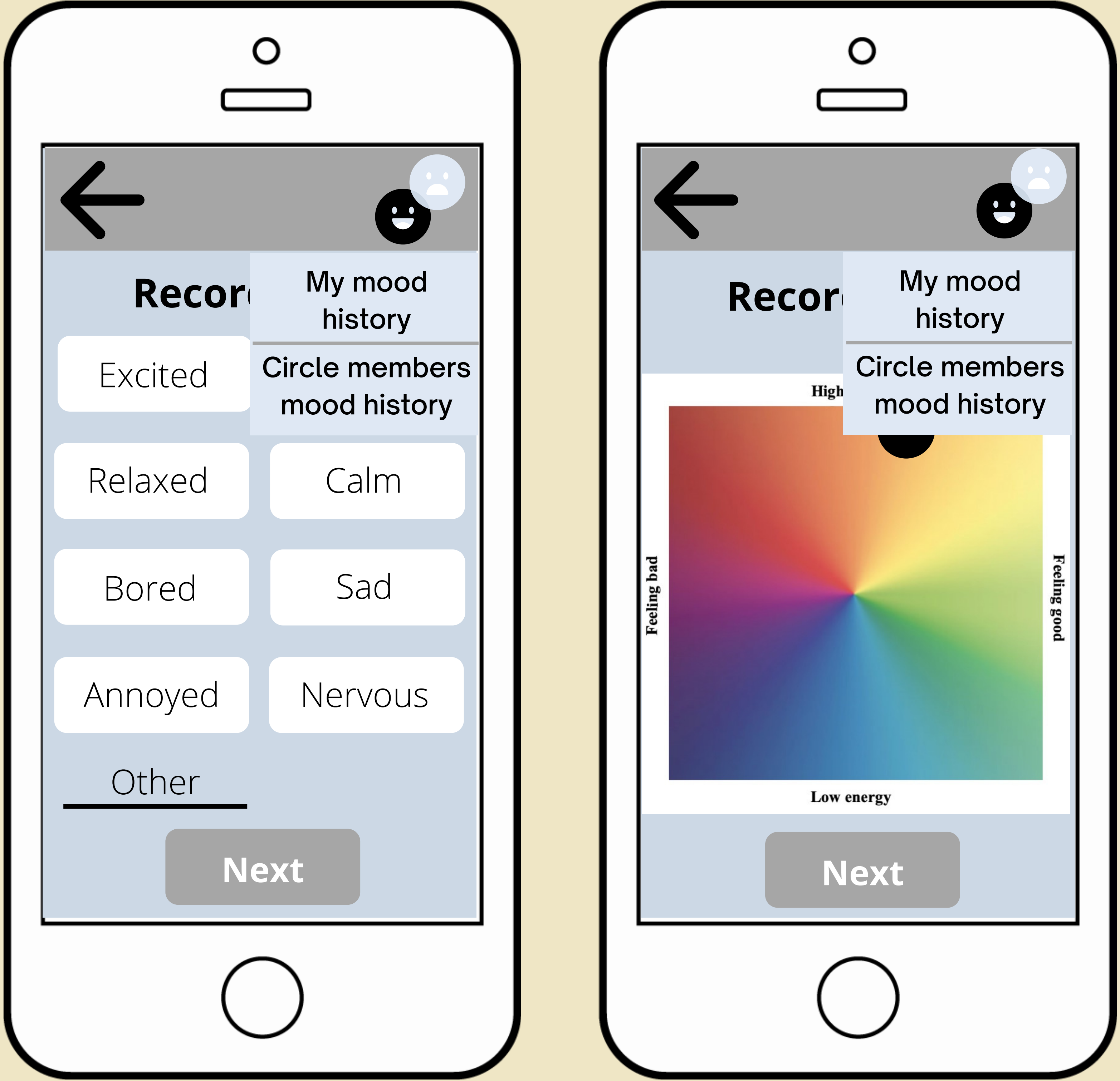
My mood history
Circle members mood history
Excited
Relaxed
Calm
Bored
Sad
Annoyed
Nervous
Other
Next
My mood history
Circle members mood history
High
Feeling bad
Feeling good
Low energy
Next

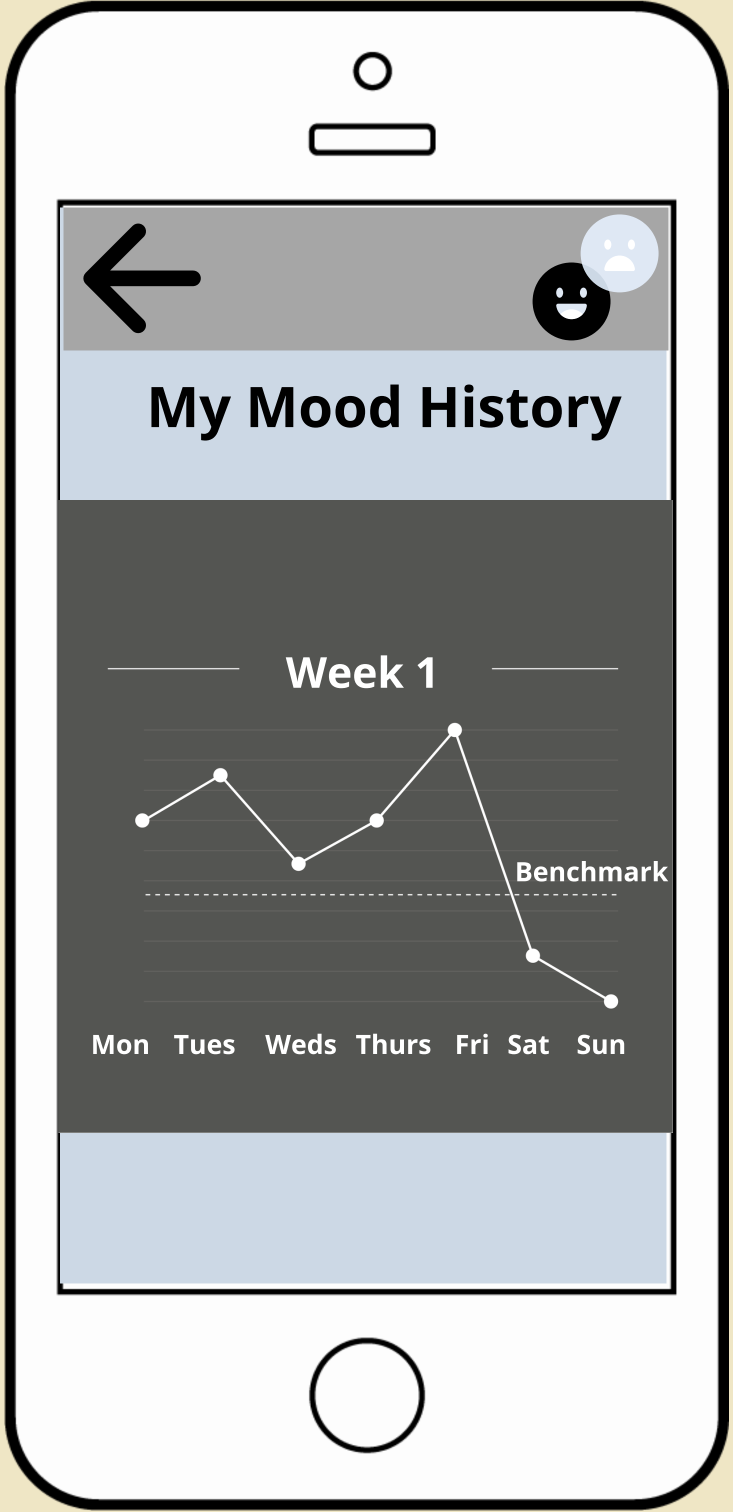
My Mood History
Week 1
Benchmark
Mon Tues Weds Thurs Fri Sat Sun

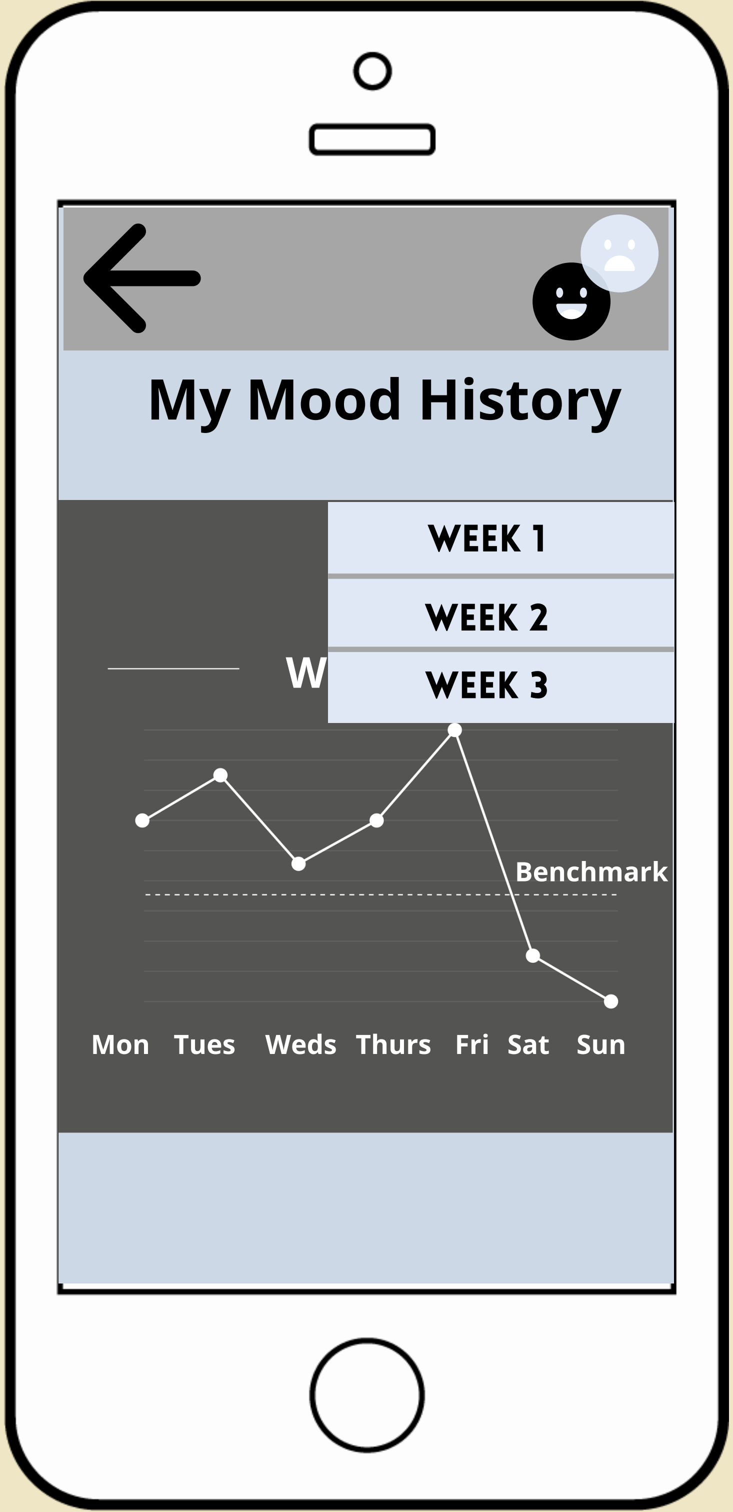

My Mood History
WEEK 1
WEEK 2
WEEK 3
W
Benchmark
Mon
Tues
Weds
Thurs
Fri
Sat
Sun

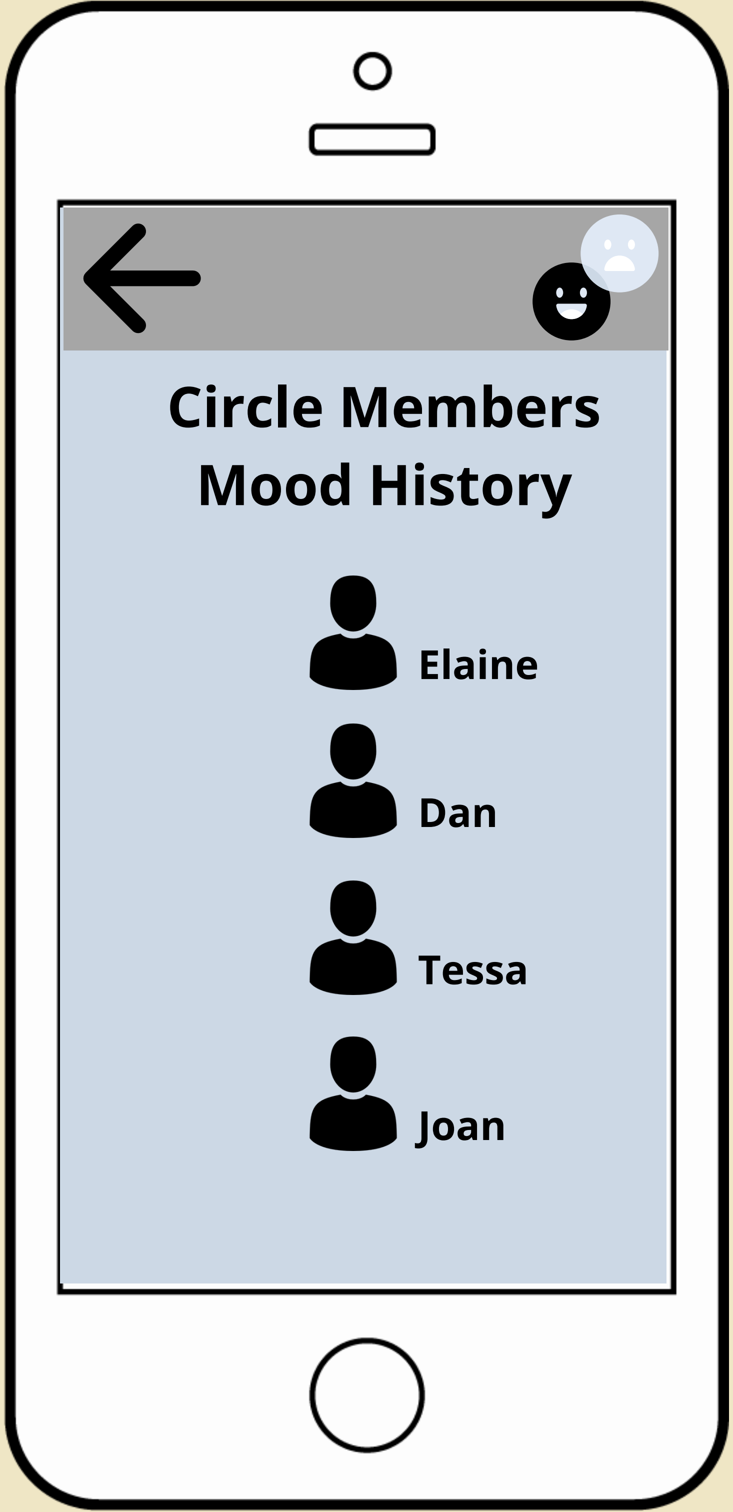
Circle Members
Mood History
Elaine
Dan
Tessa
Joan

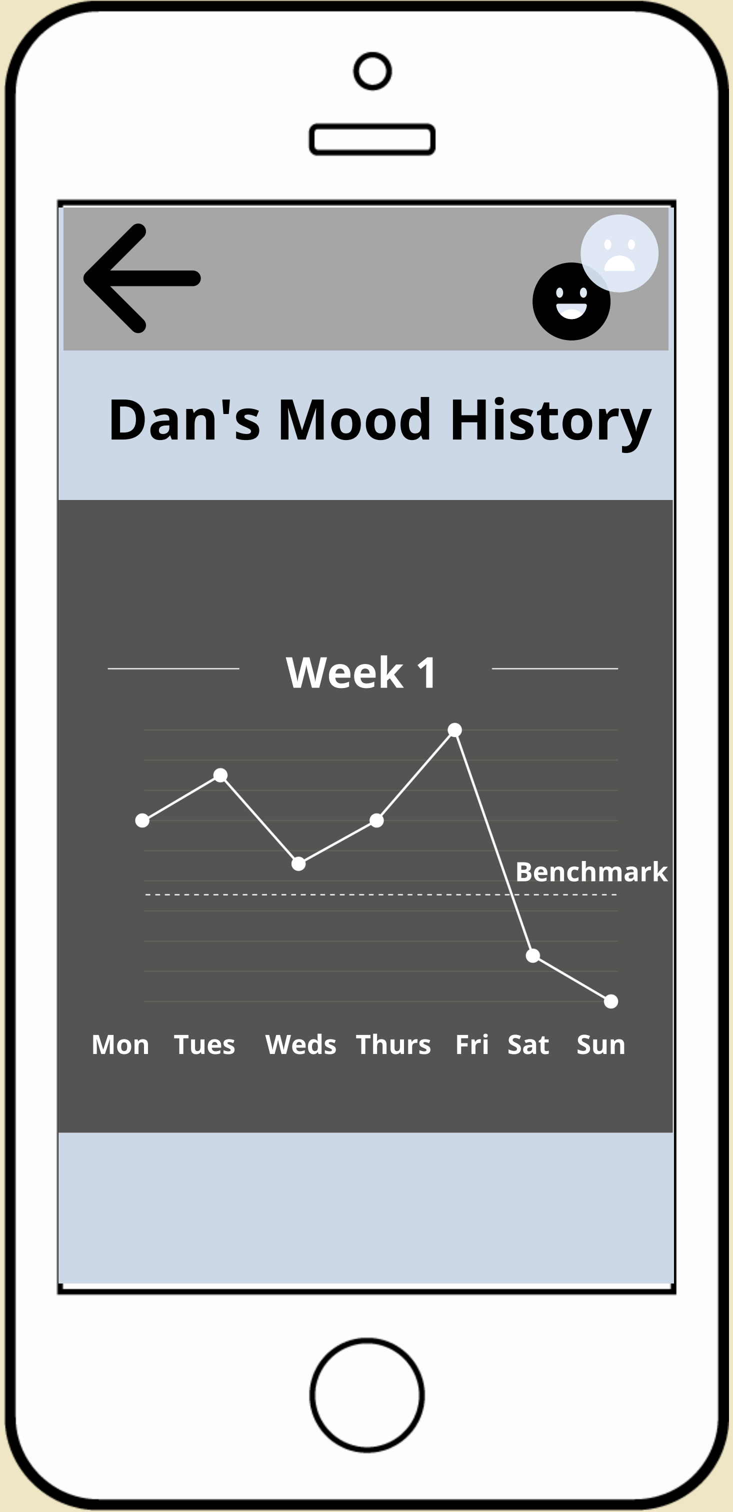
Dan's Mood History
Week 1
Benchmark
Mon
Tues
Weds
Thurs
Fri
Sat
Sun

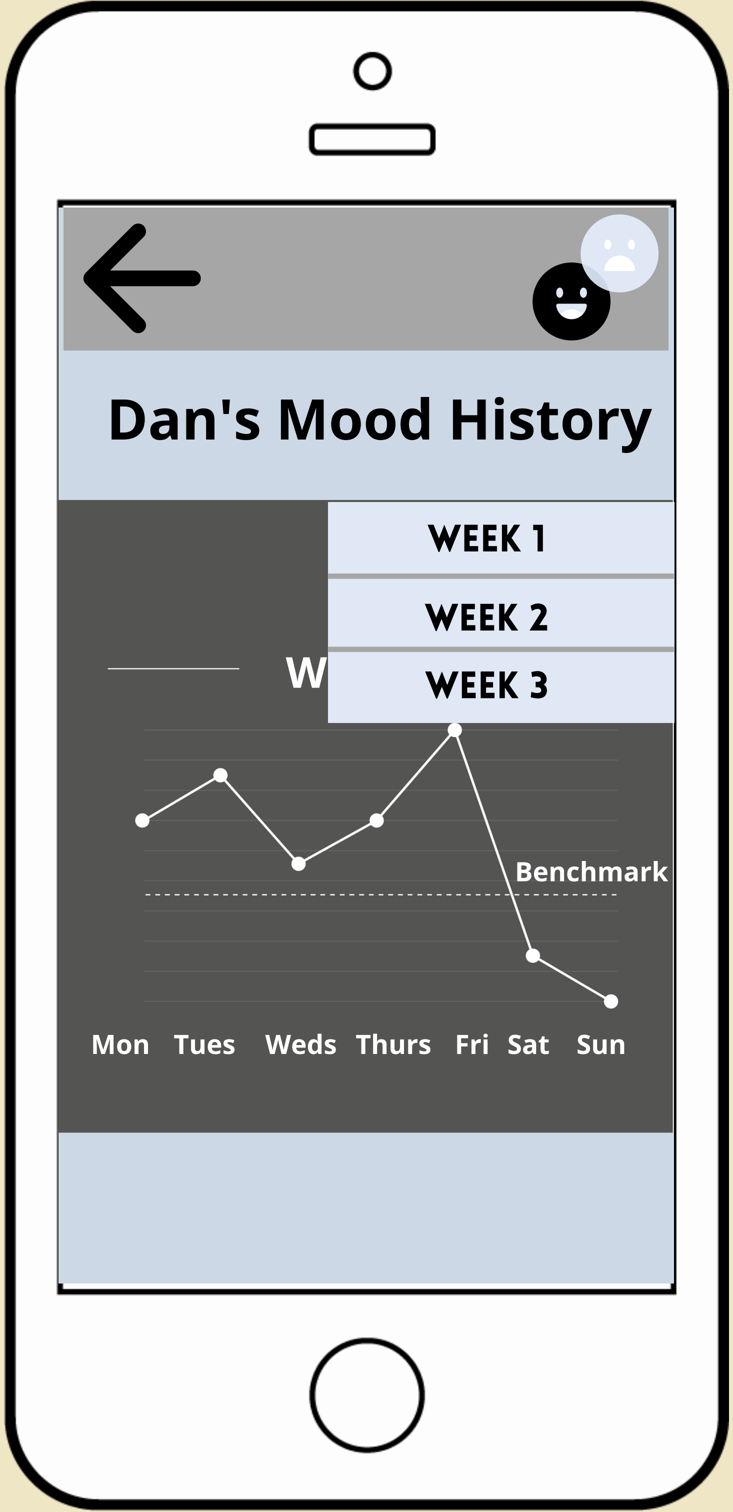
Dan's Mood History
WEEK 1
WEEK 2
WEEK 3
W
Benchmark
Mon
Tues
Weds
Thurs
Fri
Sat
Sun

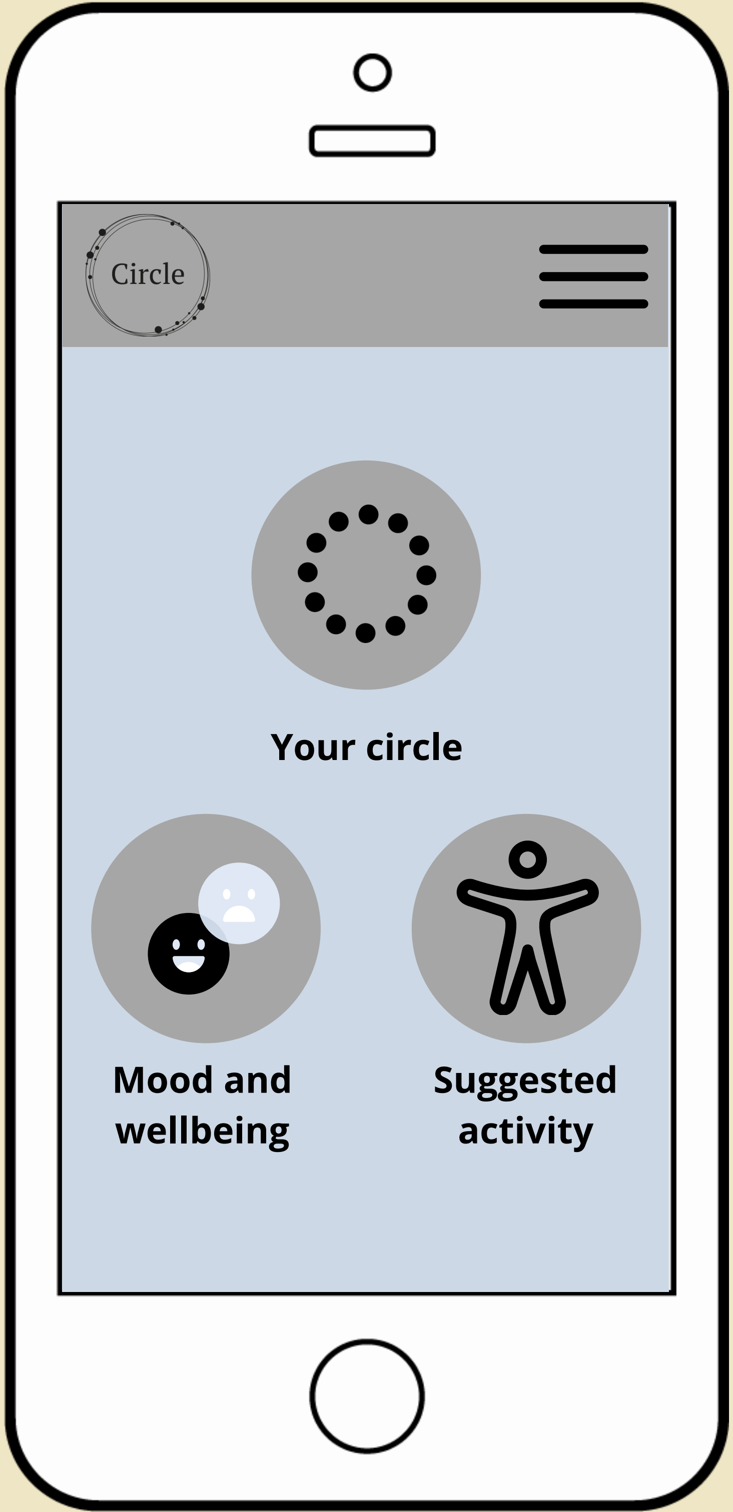
Circle
Your circle
Mood and wellbeing
Suggested activity

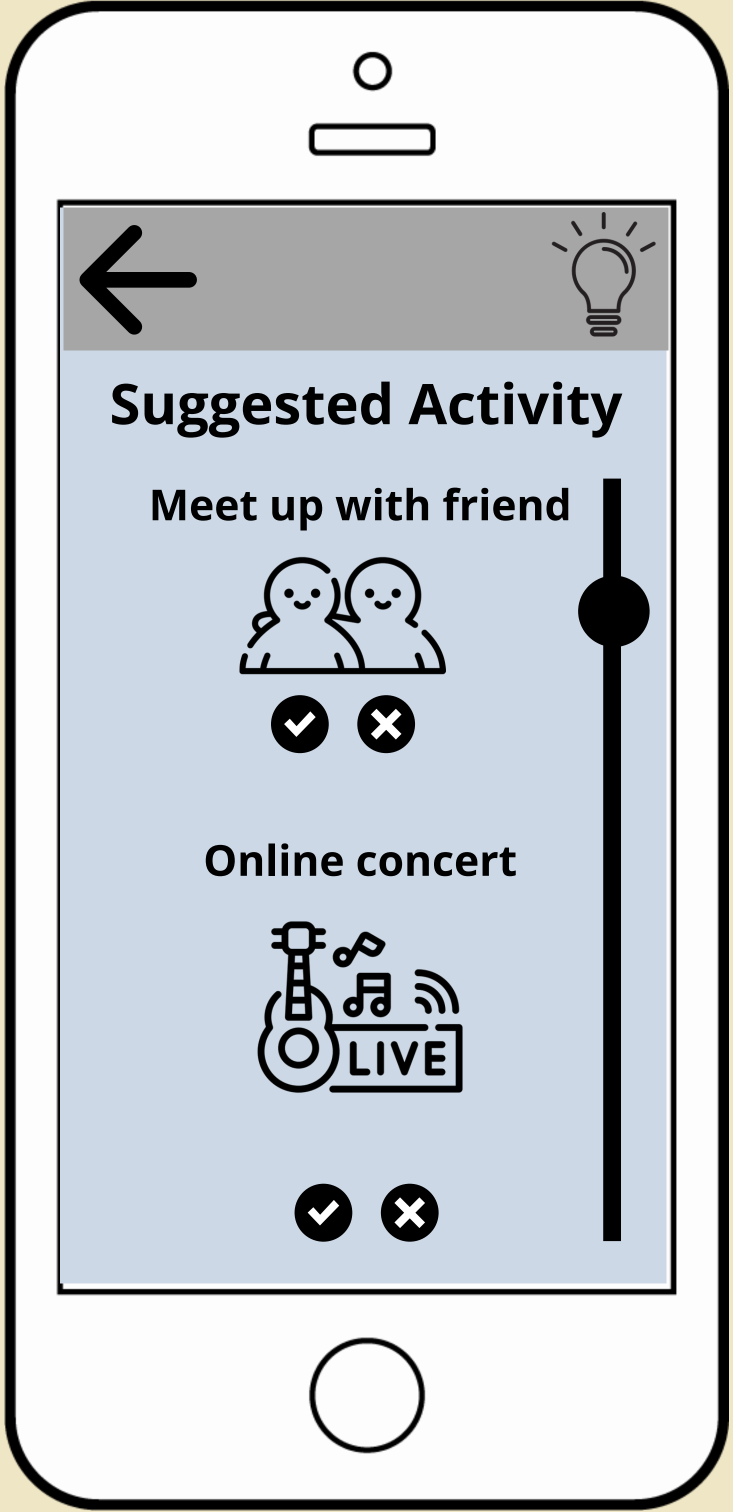
Suggested Activity
Meet up with friend
Online concert
LIVE

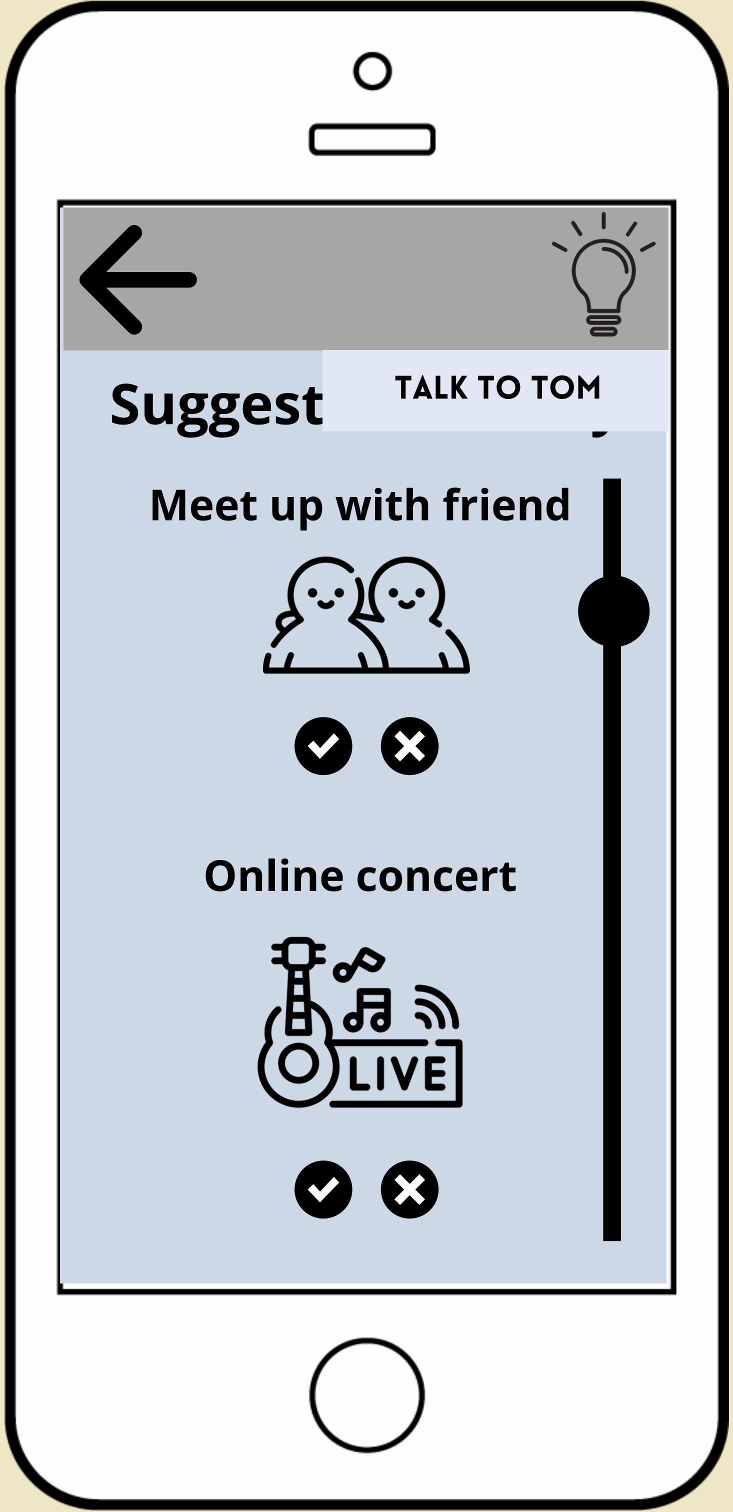
Suggest
TALK TO TOM
Meet up with friend
Online concert
LIVE

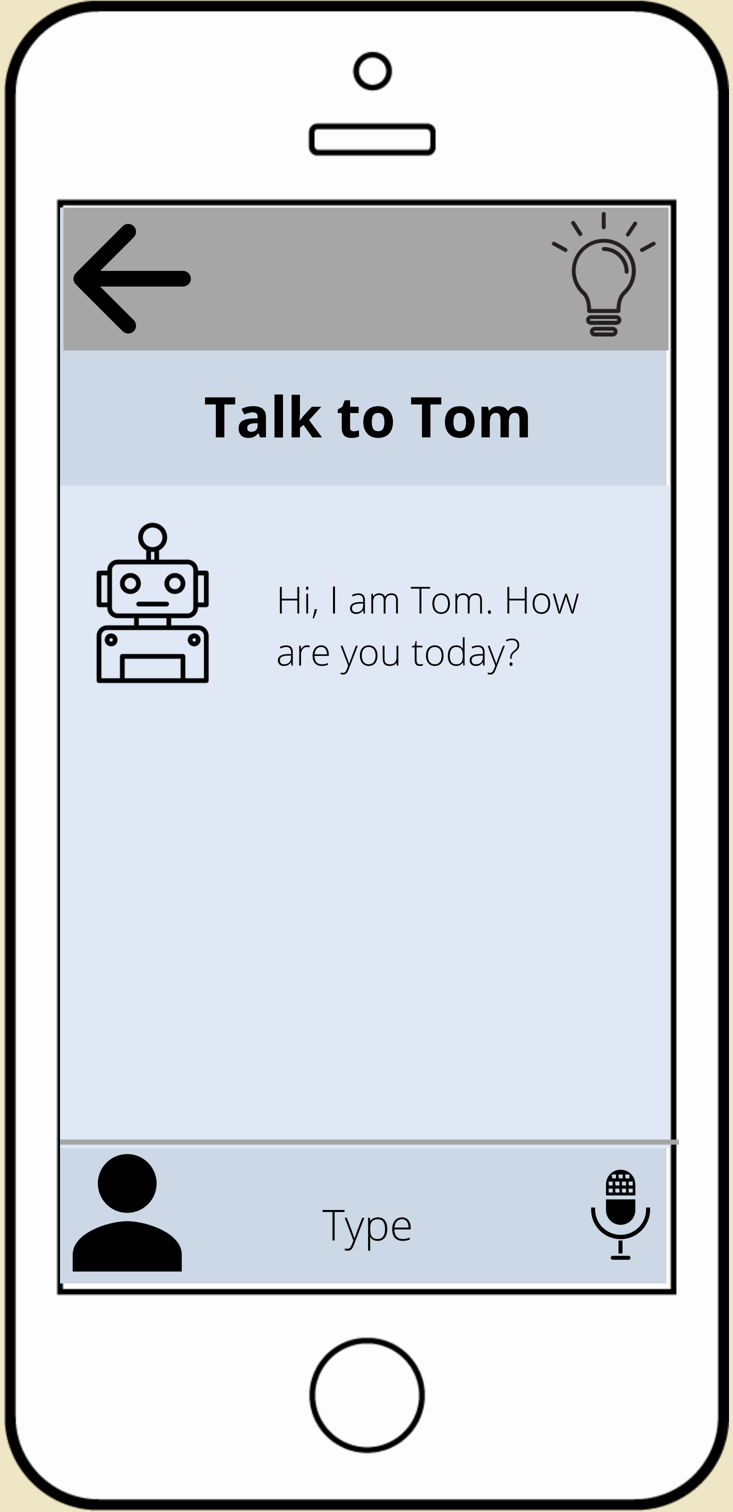
Talk to Tom
Hi, I am Tom. How are you today?
Type

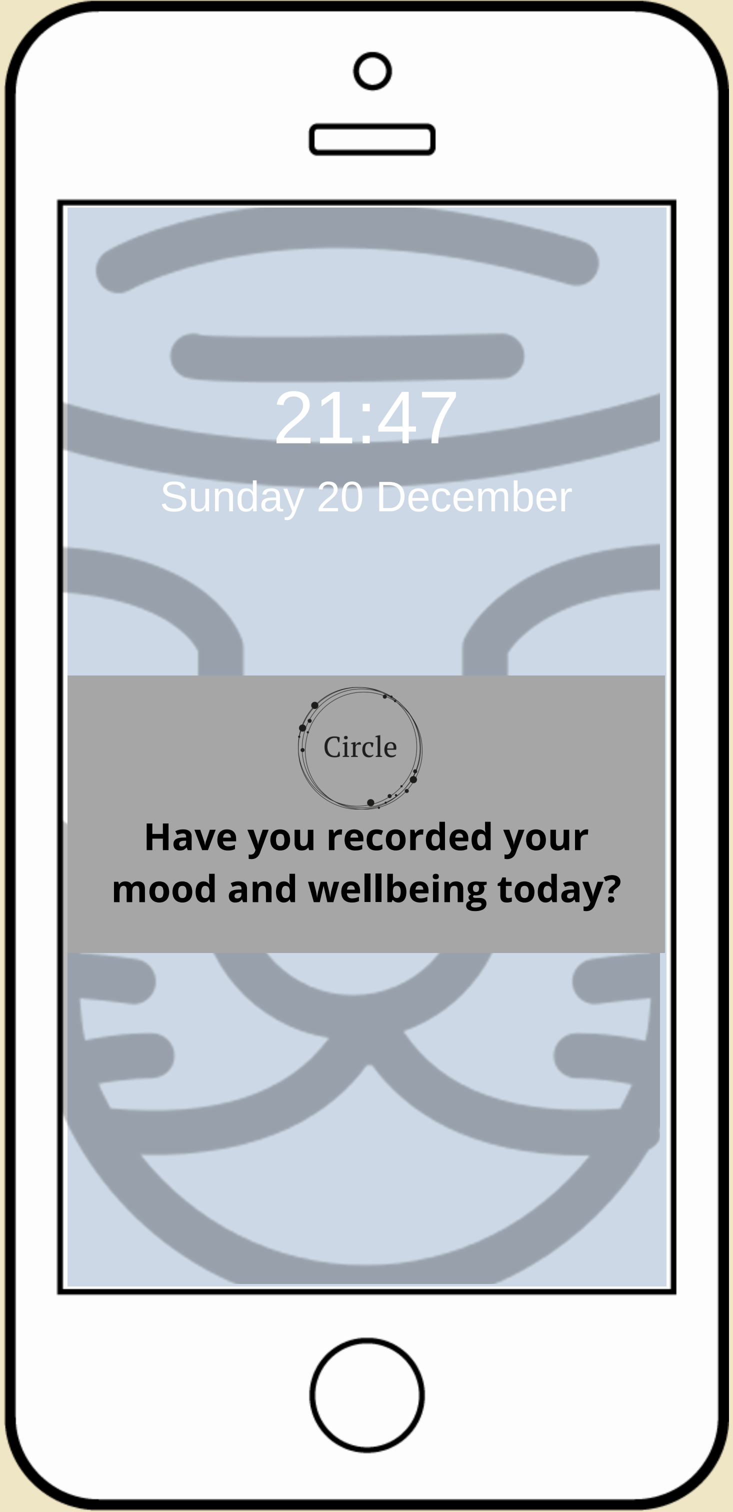
21:47
Sunday 20 December
Circle
Have you recorded your mood and wellbeing today?

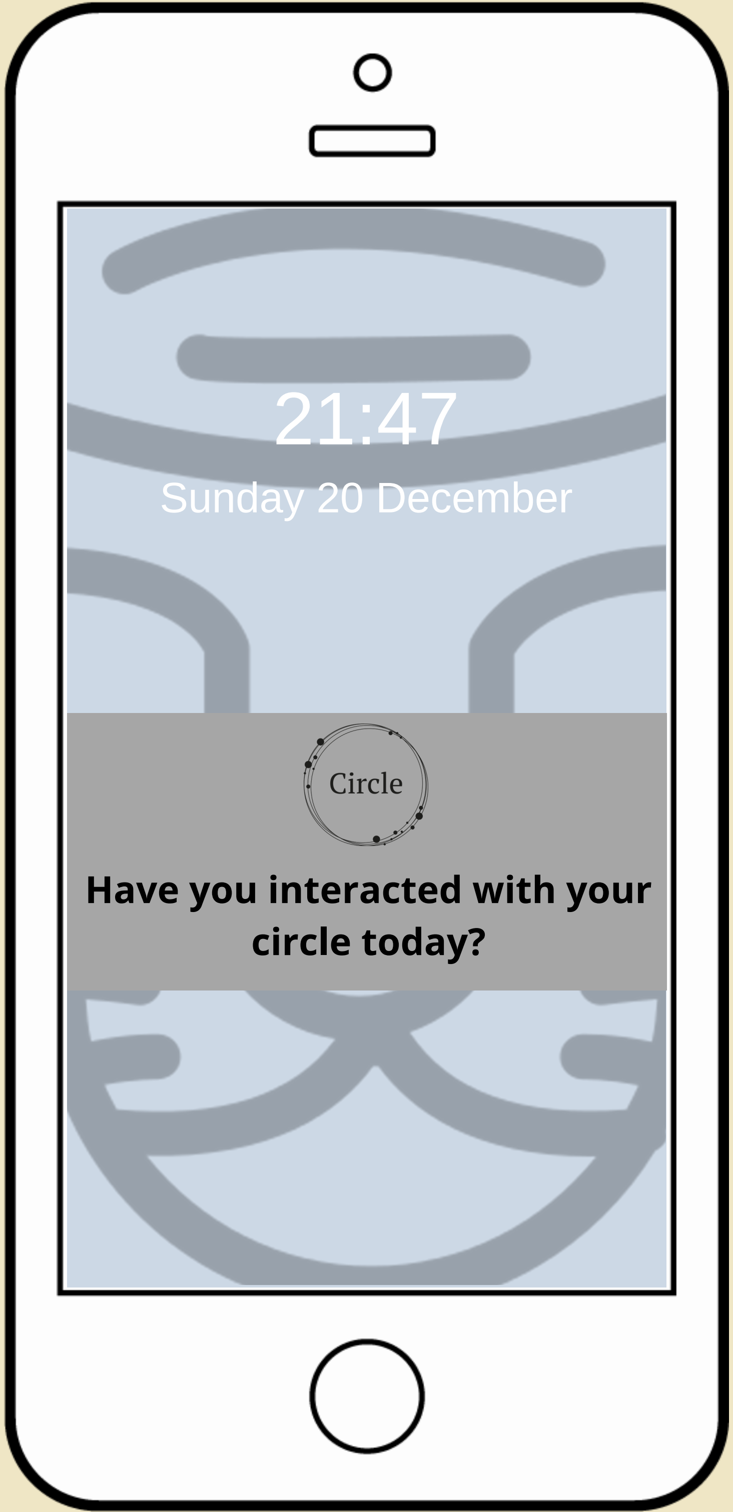
21:47
Sunday 20 December
Circle
Have you interacted with your circle today?

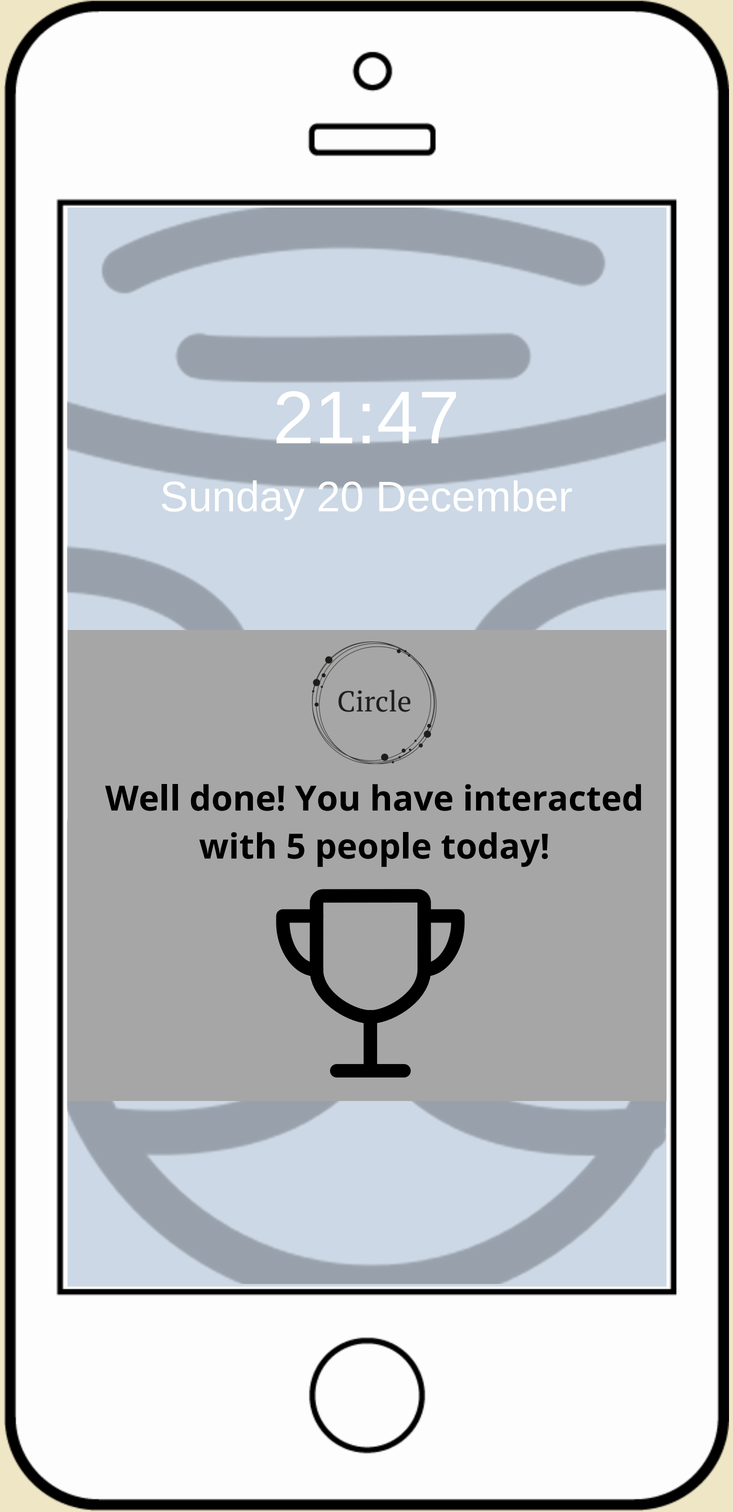
21:47
Sunday 20 December
Circle
Well done! You have interacted with 5 people today!
