# Supplementary material for: Digital Intervention in Loneliness in Older Adults: Qualitative Analysis of User Studies
Source: JMIR Form Res. 2023 Jan 27;7:e42172. doi: 10.2196/42172 (PMC9919429; doi:10.2196/42172)
Supplement: Multimedia Appendix 3 [file formative_v7i1e42172_app3.docx]

**
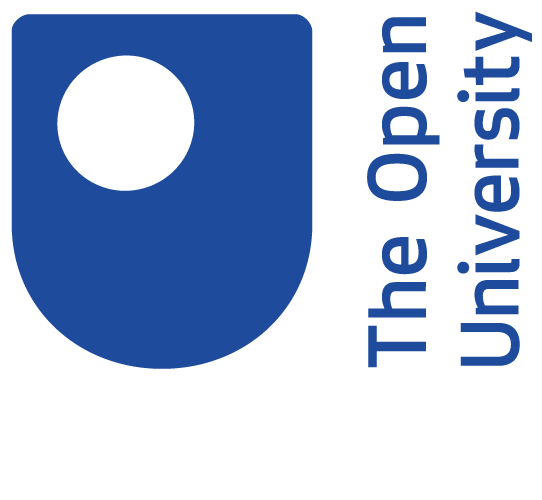
**

**Interview Questions**


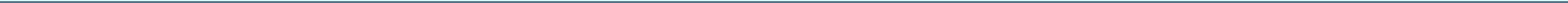


**Participant ID: ______________**

1. What have been your biggest challenges with the Covid-19 pandemic​?
2. Has the pandemic affected your emotional well-being, the activities you normally do, or how you interact with your friends and family?
3. Do you live in a single person household or with other people?
4. How would you describe your current state of mental and physical health?

************** SERVICE_Concept_Presentation_Video ***************

1. How do you feel about the SERVICE app? ​
2. Would you be willing to keep track of your moods and interactions with others, through an app like this? What would motivate you to do so?
3. How frequently would you be willing to record (daily, weekly, monthly)

Detailed breakdown as follow-up, alongside asking how frequently they would be willing to record:

Emotional status

- Loneliness level
- Mood
- General wellbeing

Time spent inside the house and outside the house

- Average time in the bedroom (excluding sleep time)
- Average time in the living room
- Average time in the kitchen
- Average time spent outside

Interactions either with an individual of group

- Including who, how achieved, quality of the interaction

1. What kind of activities would you like the SERVICE app to suggest to you?
2. What concerns do you have about the SERVICE app or what it does?
3. How would you feel about you and your support networking using the SERVICE app? ​
4. How would you change the SERVICE app to make it more useful?

************** SERVICE_Draft_Screen_Designs ***************

We’re now going to show you a number of draft designs for what the app may look like. For each screen, we’re going to ask you what you like about the design, and what you think needs to change.
