# Supplementary material for: Digital Intervention in Loneliness in Older Adults: Qualitative Analysis of User Studies
Source: JMIR Form Res. 2023 Jan 27;7:e42172. doi: 10.2196/42172 (PMC9919429; doi:10.2196/42172)
Supplement: Multimedia Appendix 4 [file formative_v7i1e42172_app4.docx]

**Using CIRCLE App to help older people: Interview guide (Study 3 usability trial)**

1. Introduction & Setting Ground Rules

- The SERVICE project is developing an interactive mobile app for older people with the aim to tackle loneliness and improve wellbeing. I will ask some questions about your experiences and attitudes of using the prototype of the app for the past week.
- There are ‘no right or wrong answers’ for this interview and you will not be judged by your answers. So it doesn’t matter if there were parts of the app you didn’t like or use, we would like to know what you think. Your feedback will help us improve the app in the future.
- Everything you say in this interview is confidential. A pseudonym name will be assigned to you and all the identifying details will be removed. This interview is video-recorded so that I can accurately transcribe and analyse our conversation.
- This interview is expected to last between an hour and an hour fifteen minutes. Are you willing to take part in this interview? Do you have any questions?

2. Icebreaker

- General questions about participants themselves and how they have found the COVID-19 pandemic.

Probes: Living changes; Activity changes; Lifestyle changes during COVID-19

- Can you tell me a little bit about yourself?
- We are now experiencing COVID-19 pandemic (although the measures have been eased on Monday), How has covid-19 impacted your life?
- Use of technology to manage any of these changes?
- Have you used apps for wellbeing in the past? (If so) Tell a bit about how you found using it.

3. Acceptability; Satisfaction with the App

*For the past week, we’ve advised you to use the app every day, …*

- On a scale 1-10, could you please rate how much did you enjoy the use of the app? With 1 = did not enjoy at all, 10 = enjoyed very much? Can you explain your score? What overall did you enjoy about the app? What overall did you not enjoy about the app?
- In general, what do you think about the app?
- How do you feel about logging wellbeing?
- How do you feel about reflecting on your wellbeing?
- Which part of the app did you enjoy the most? What did you like about it?

4. Demand – What are the levels of participants usage of the app?

- How often did you use the app for the past week? How did you find the experience of using the app for a week (this length of time)?

Probe: How often did you open the app?

How often did you log your wellbeing? Either via ‘my mood’ or ‘daily record’

How often did you look at your circle?

- Do you feel this product was designed for you? Why?
- What did you think was the overall purpose to this app?

5. Implementation – To what degree was the app used as it was intended?

- To what degree is this app useful for reflecting on your social circle?
- Can you tell me a little about how you organised people within your social circle

Prompts: Who did you put in your inner circle? Who did you put in the outer circle? Why did you put them there?

- Tell me more about, to what degree is this app useful for reflecting on your wellbeing?
- Did you have any unexpected feelings or reflections from using the app?

6. Practicality – How easy or difficult was the app to use?; Technical issues; Easy to use

- On a scale 1-10, could you please rate how easy the app to be used? (1 = difficult, 10 = easy) Can you explain your score?
- Were there any practical constraints in your use of the app?

Probe: resources, time etc.

- Could you find your way around?

Probe: the daily record, my circle, setting, my mood

- What did you find difficult to use within the app?
- What did you find easy to use in the app?
- What did you think is the most necessary within the app?
- What did you think is the least necessary within the app?
- What would you like to see more of within My Mood?
- What would like to see more of within My Circle?
- What would like to see more of within Daily Record?

7. Adaption/ Improvement; Improvement/Enhancement

- What suggestions do you have to help us improve the app?
  - If you had had a magic wand, what would you change about this product?
  - What modifications to the app would make it easier to use? (What would make it more practical to use?)
  - What would make it more enjoyable or rewarding to use?

8. Integration (into existing health/wellbeing management)

- On a scale 1-10, could you please rate how much this app was fitted in your existing health and well-being routine? Can you explain your score?
- Can you tell me about how you usually reflect on your social circle?
- In general, how did this app make you feel this week?

9. Efficacy – Is there evidence of a positive effect from the use of the app?; Consequences/impacts

- On a scale 1-10, could you please rate how much the use of the app impacted your wellbeing – where 1 = detrimental to wellbeing, 5 = neutral, and 10 = very beneficial to wellbeing? Can you explain your score?
- How did this app make you feel?

Probe: well-being, noticeable impacts

- Would you recommend it to anyone? Who?
  - Could you see it being effective for other groups of people?
  - or in some circumstances?
  - or over a longer time period?
  - Friends? Family members?
- Would your friendship circle use this app together?
- How did this app make you think about your social circle?
- How does this app affect your thoughts about your social connections?

1. Safety; Perceived issues

- On a scale 1-10, could you please rate the safety of using the app? Where 1 = not safe, 10 = very safe; Can you explain your score?
- Were there any negative effects on how you felt or how you thought about your social connections?
- Can you think of ways in which the app might have a negative effect on people?

1. Conclusion/Debrief

*So as we come to the end of the interview, I’d like to check*

- Is there anything that we haven’t touched upon yet that you would like to share?

**Ending – chat with participant:**

That’s the end of the interview:

- How did you find this interview?
- How did you find this trial?
- Any exciting plans after the interview?
- Ethics reminder: As I mentioned at the beginning of the interview, A pseudonym name will be assigned to you and all the identifying details will be removed. That means this interview won’t be linked back to you. If you have any concerns about the information that you shared and would like to withdraw from the study, please contact the research team within two weeks. After two weeks, you can’t withdraw as we will use the pseudonym name to anonymised the information.
- **£20 Amazon Voucher Code** will be sent to your email address. Do you know how to use the Amazon Voucher code on Amazon?
