# Supplementary figures and images for: Digital Intervention in Loneliness in Older Adults: Qualitative Analysis of User Studies
Source: JMIR Form Res. 2023 Jan 27;7:e42172. doi: 10.2196/42172 (PMC9919429; doi:10.2196/42172)

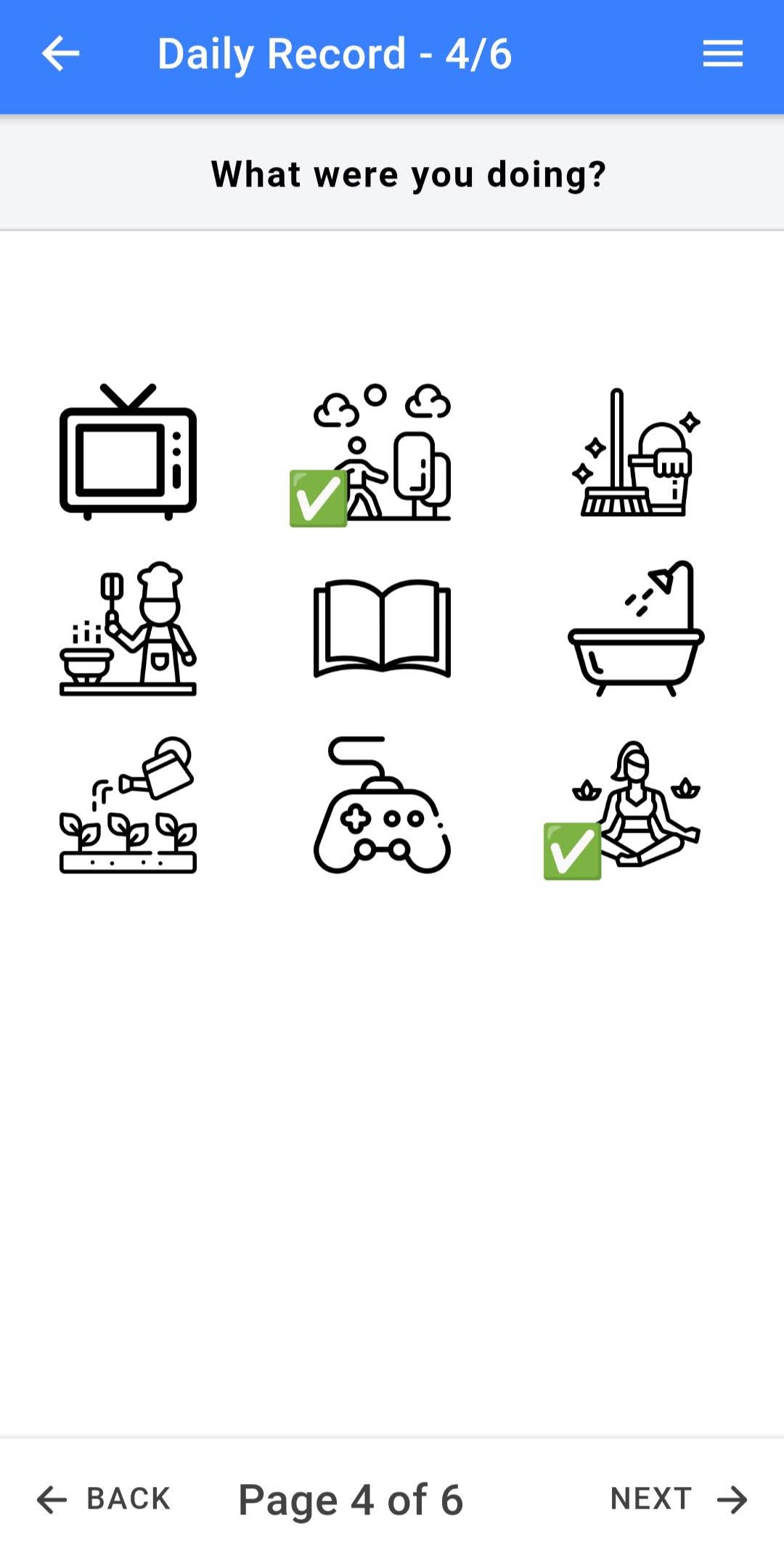

Supplement: Multimedia Appendix 5 [file formative_v7i1e42172_app5.zip › original_004159e1-4a15-46e5-acf6-8585a55efed2_Screenshot_2021-12-03-17-39-51-14_b18fac2f20e3d3dbceaad755ce160a22.jpg]

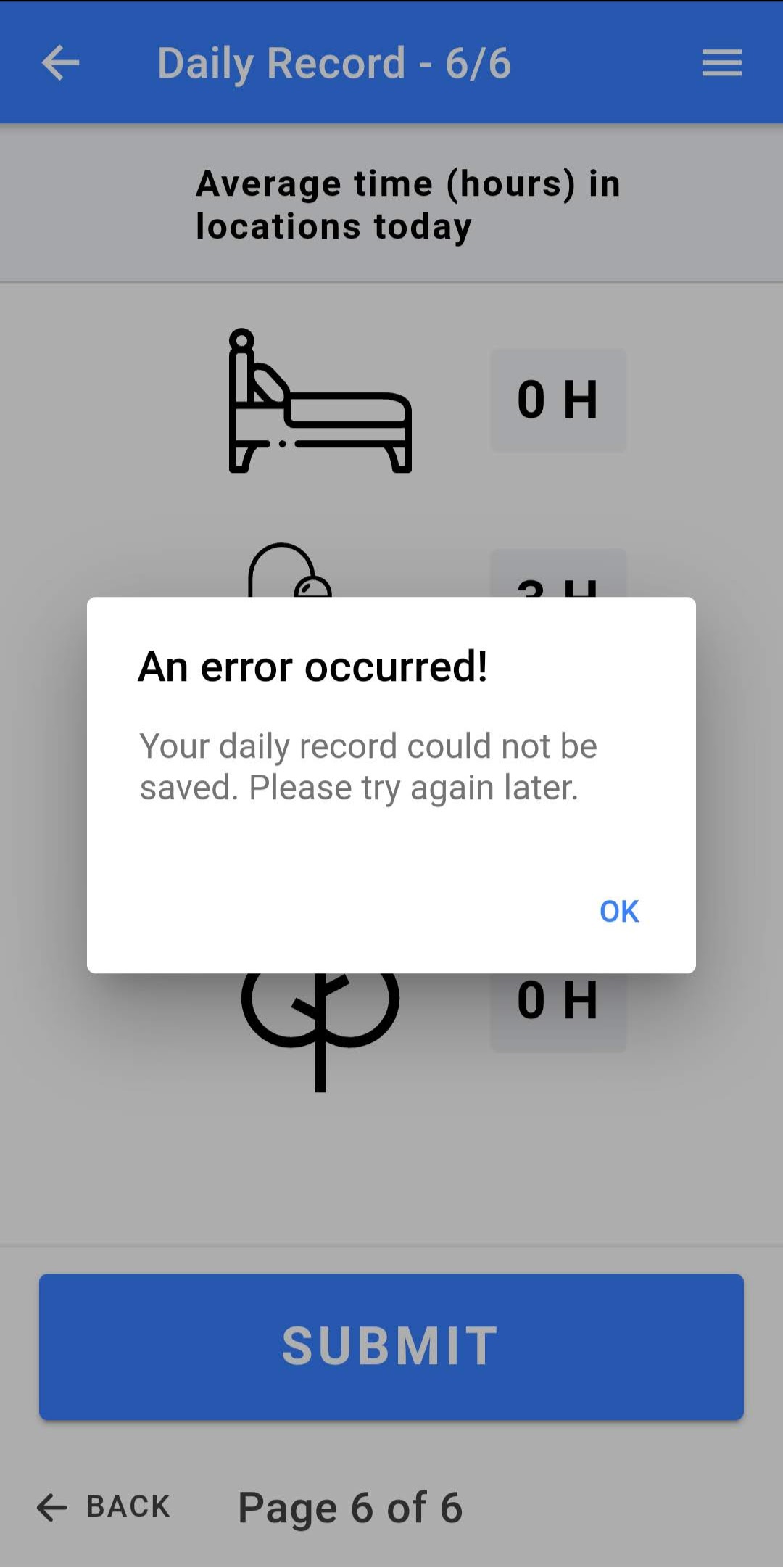

Supplement: Multimedia Appendix 5 [file formative_v7i1e42172_app5.zip › original_2dbed32a-d838-43be-9060-1d2df6ad79fa_Screenshot_2021-12-03-17-40-22-58_b18fac2f20e3d3dbceaad755ce160a22.jpg]

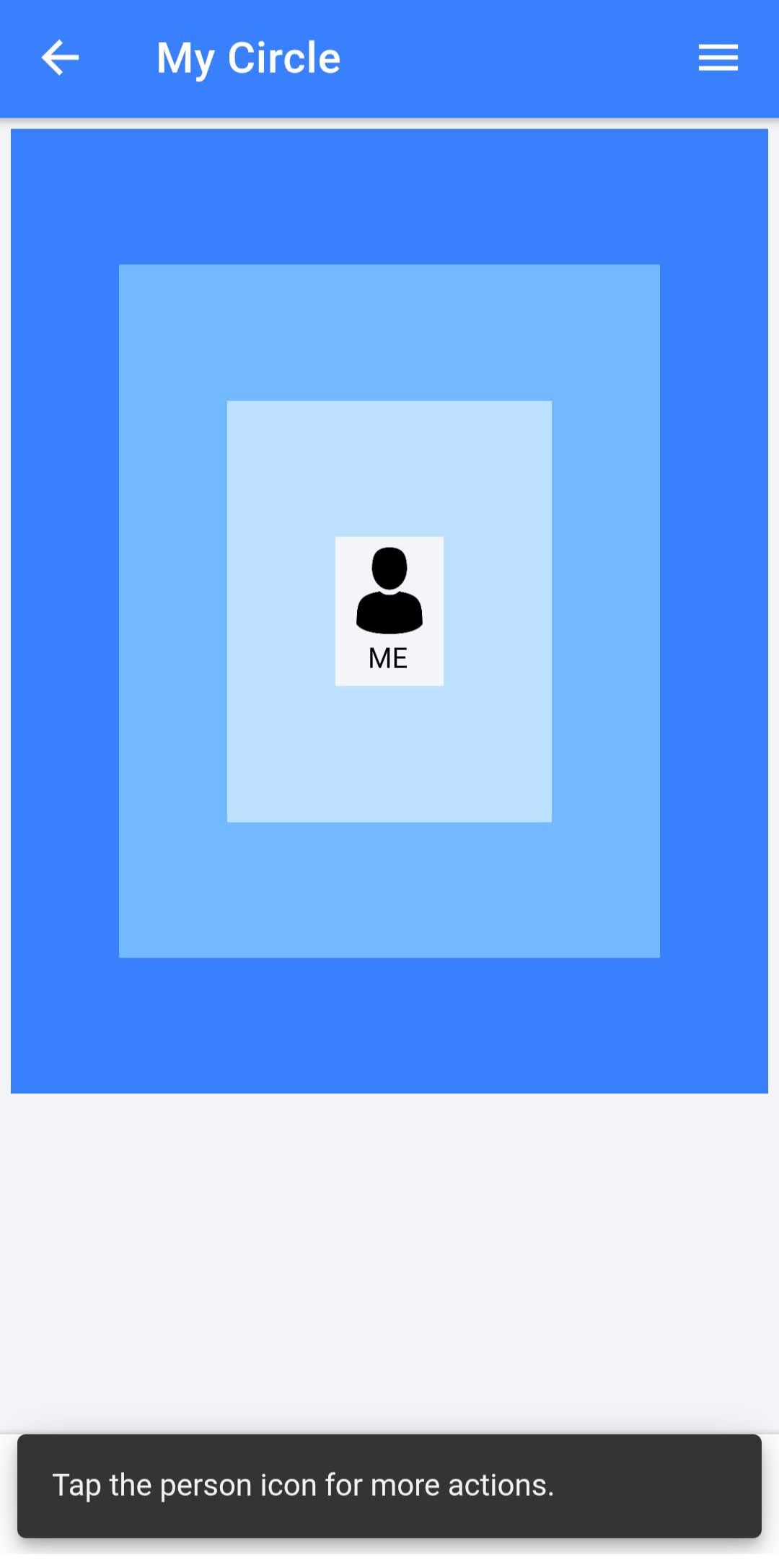

Supplement: Multimedia Appendix 5 [file formative_v7i1e42172_app5.zip › original_49f06f31-0509-47c4-bd35-ca5d6e4e4505_Screenshot_2021-12-03-17-40-33-77_b18fac2f20e3d3dbceaad755ce160a22.jpg]

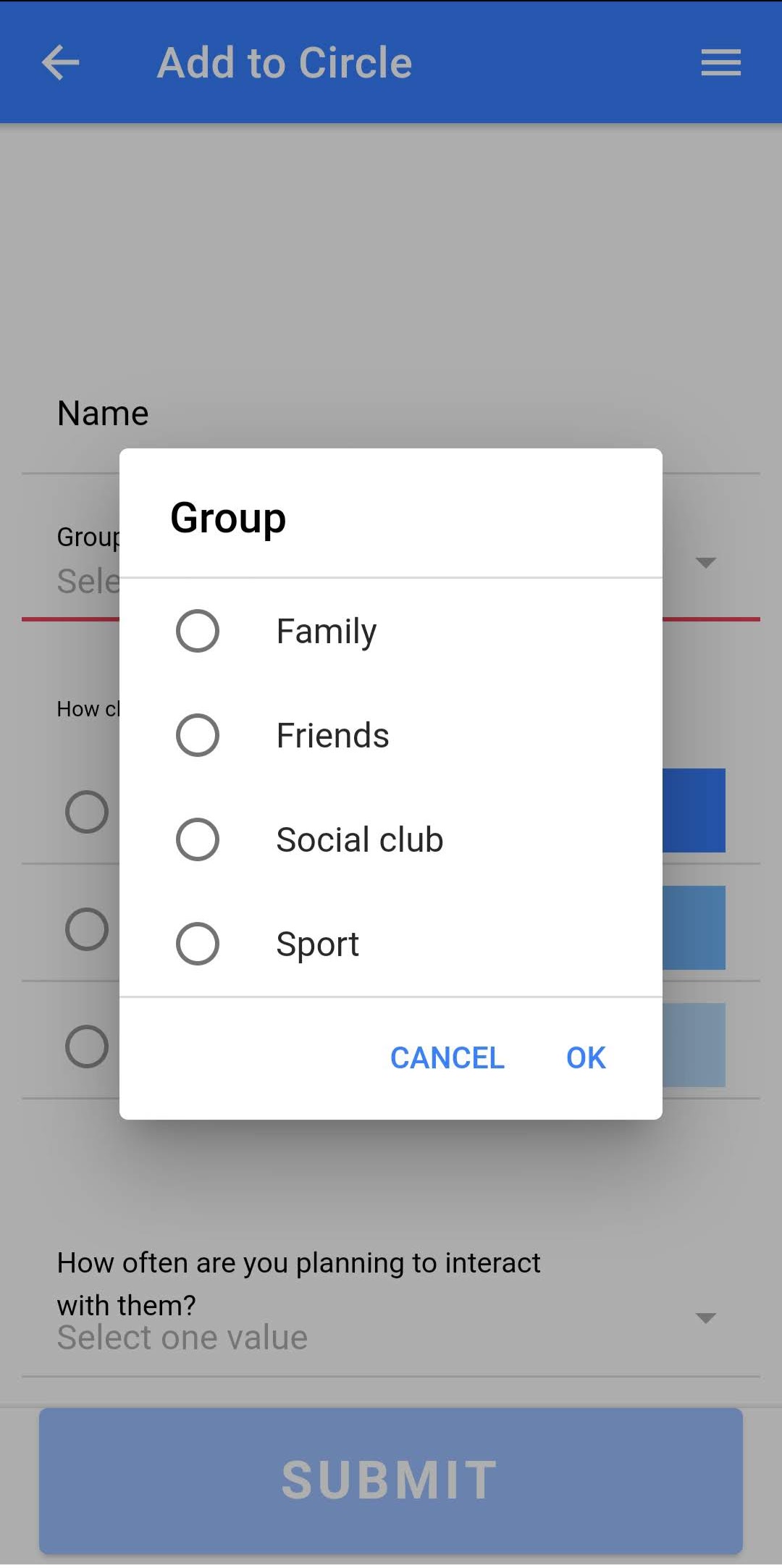

Supplement: Multimedia Appendix 5 [file formative_v7i1e42172_app5.zip › original_66c2160c-e4ab-4e25-827a-6a6381c89da3_Screenshot_2021-12-03-17-40-44-65_b18fac2f20e3d3dbceaad755ce160a22.jpg]

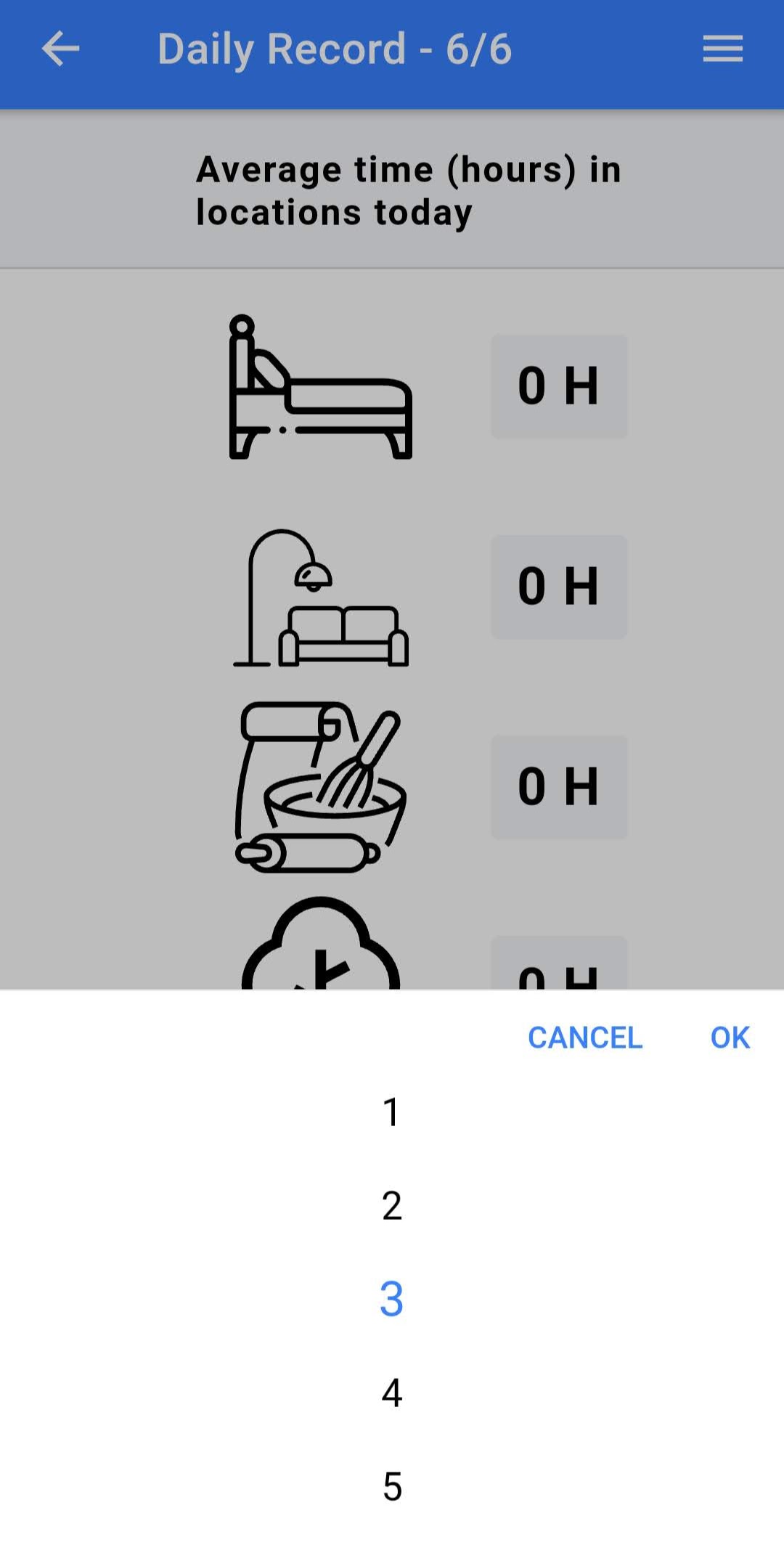

Supplement: Multimedia Appendix 5 [file formative_v7i1e42172_app5.zip › original_6882d293-84df-4026-b21e-e58049ce3986_Screenshot_2021-12-03-17-40-11-73_b18fac2f20e3d3dbceaad755ce160a22.jpg]

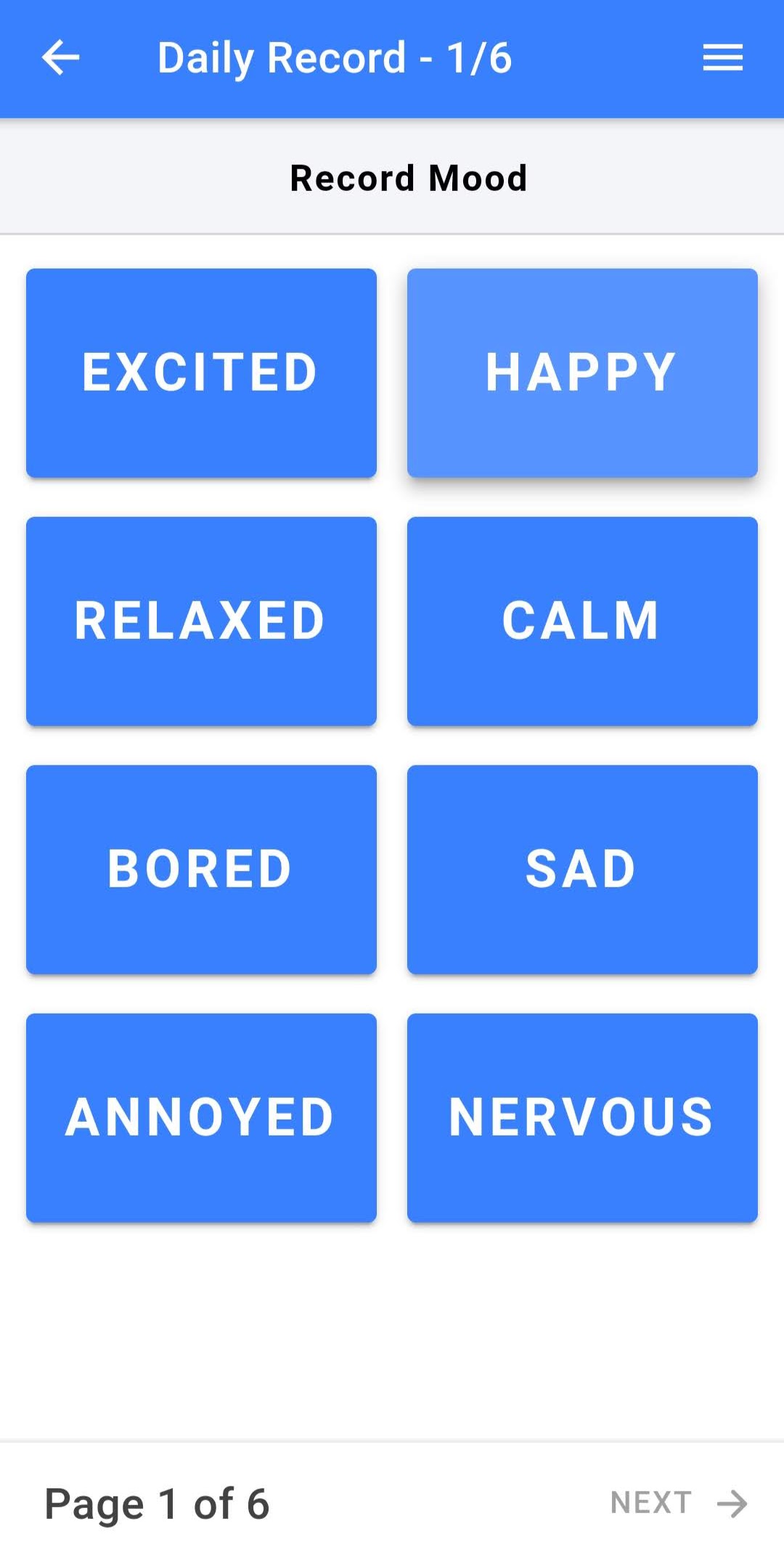

Supplement: Multimedia Appendix 5 [file formative_v7i1e42172_app5.zip › original_6ef8df55-80b1-4b64-aff2-aa8e5b528b5d_Screenshot_2021-12-03-17-39-28-90_b18fac2f20e3d3dbceaad755ce160a22.jpg]

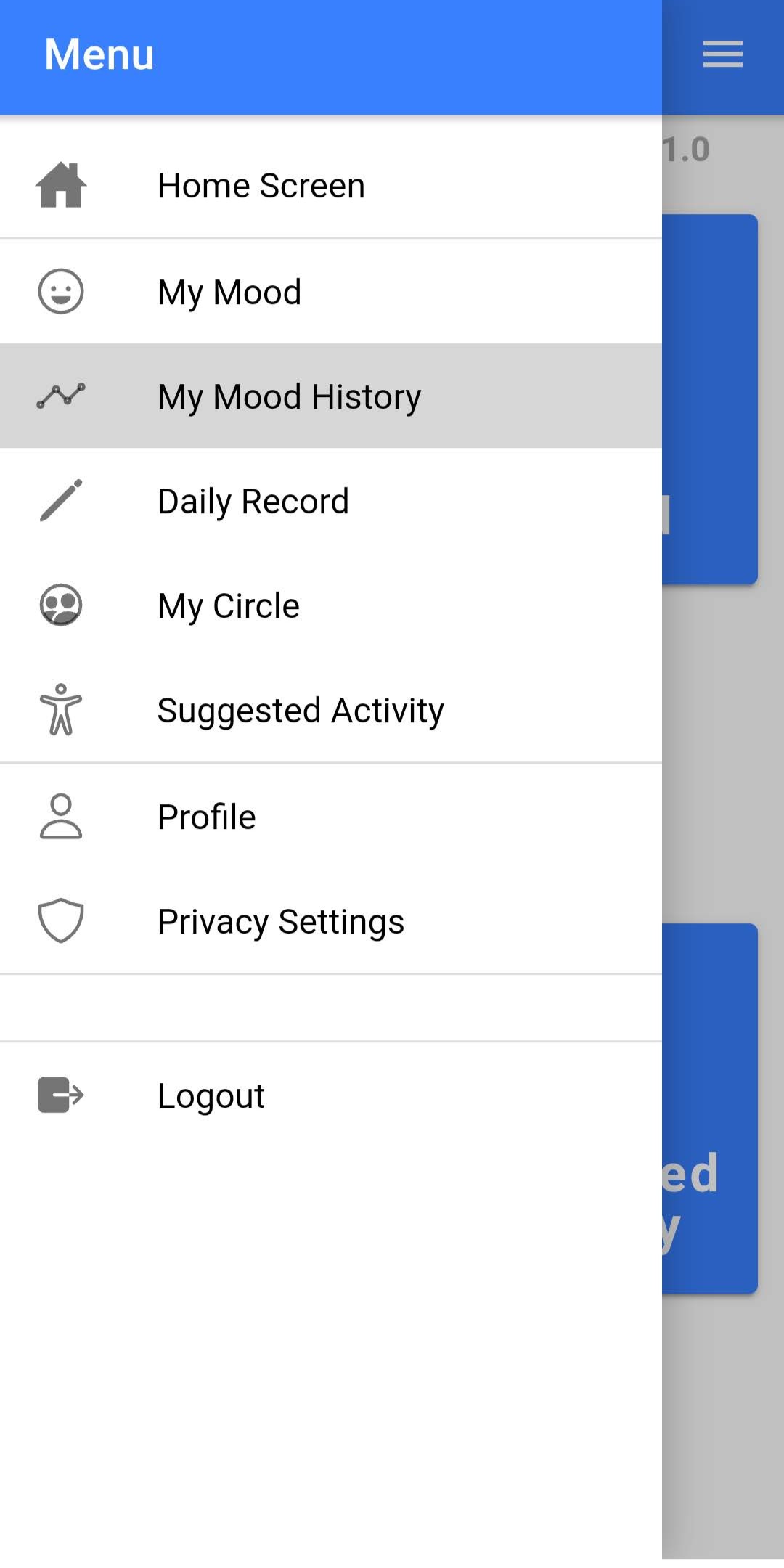

Supplement: Multimedia Appendix 5 [file formative_v7i1e42172_app5.zip › original_8e42d17a-0b81-4cca-988f-23e318eca76e_Screenshot_2021-12-03-17-41-02-44_b18fac2f20e3d3dbceaad755ce160a22.jpg]

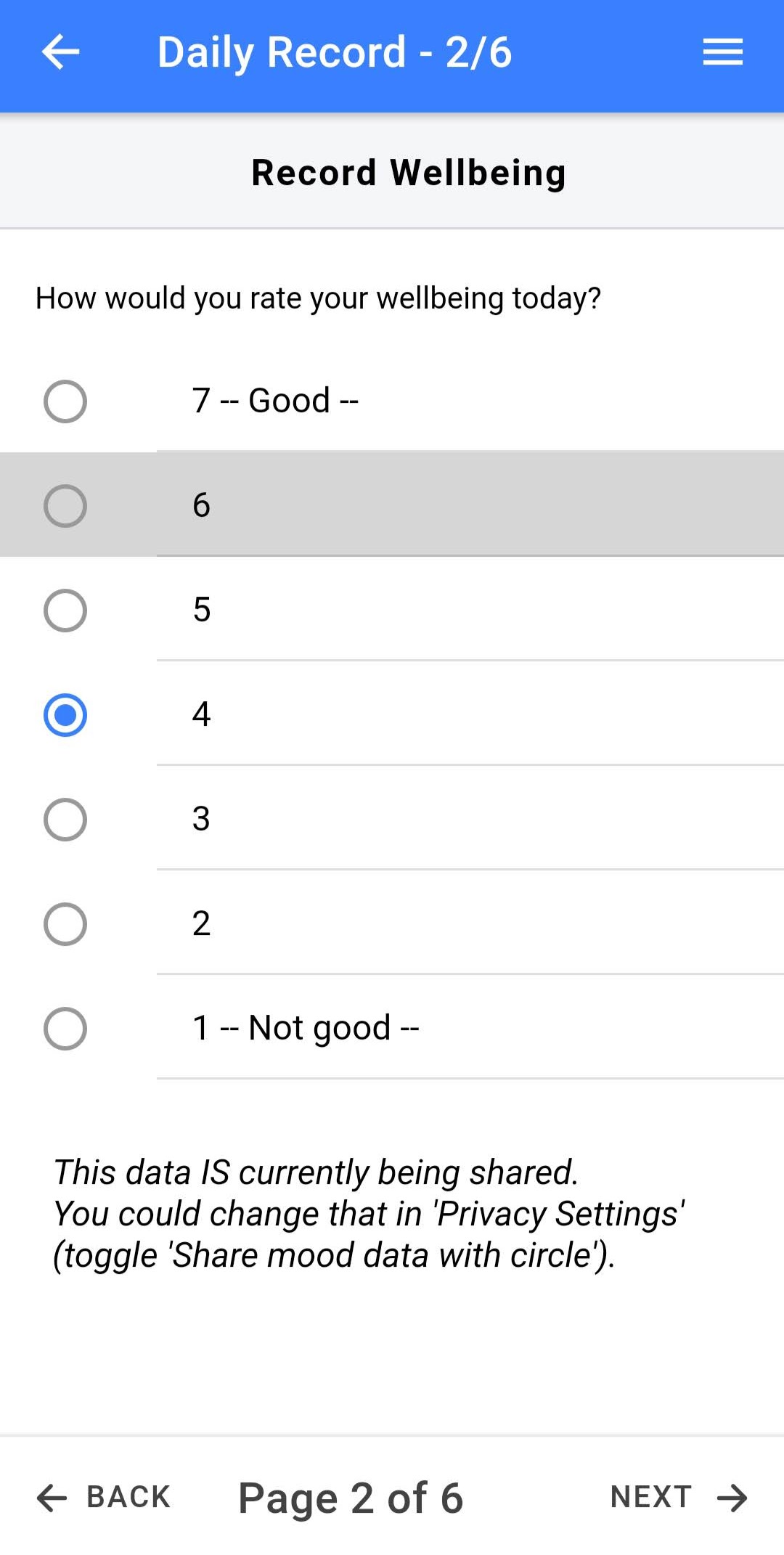

Supplement: Multimedia Appendix 5 [file formative_v7i1e42172_app5.zip › original_8f4ccbdb-f5c6-4400-9e05-5b5d1e844eda_Screenshot_2021-12-03-17-39-38-57_b18fac2f20e3d3dbceaad755ce160a22.jpg]

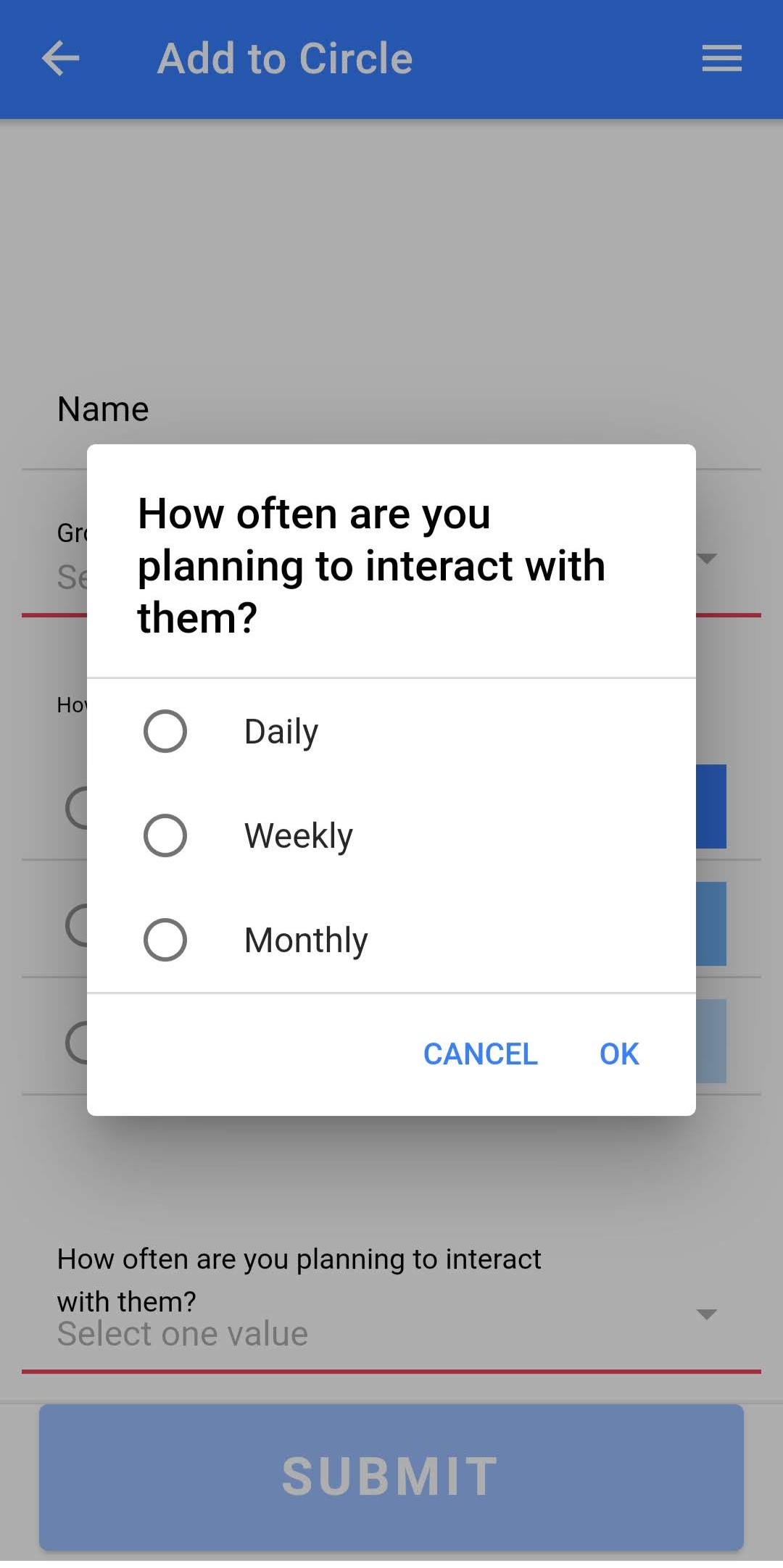

Supplement: Multimedia Appendix 5 [file formative_v7i1e42172_app5.zip › original_9916b2cd-20d5-4cdb-9685-c0deb22f6694_Screenshot_2021-12-03-17-40-49-50_b18fac2f20e3d3dbceaad755ce160a22.jpg]

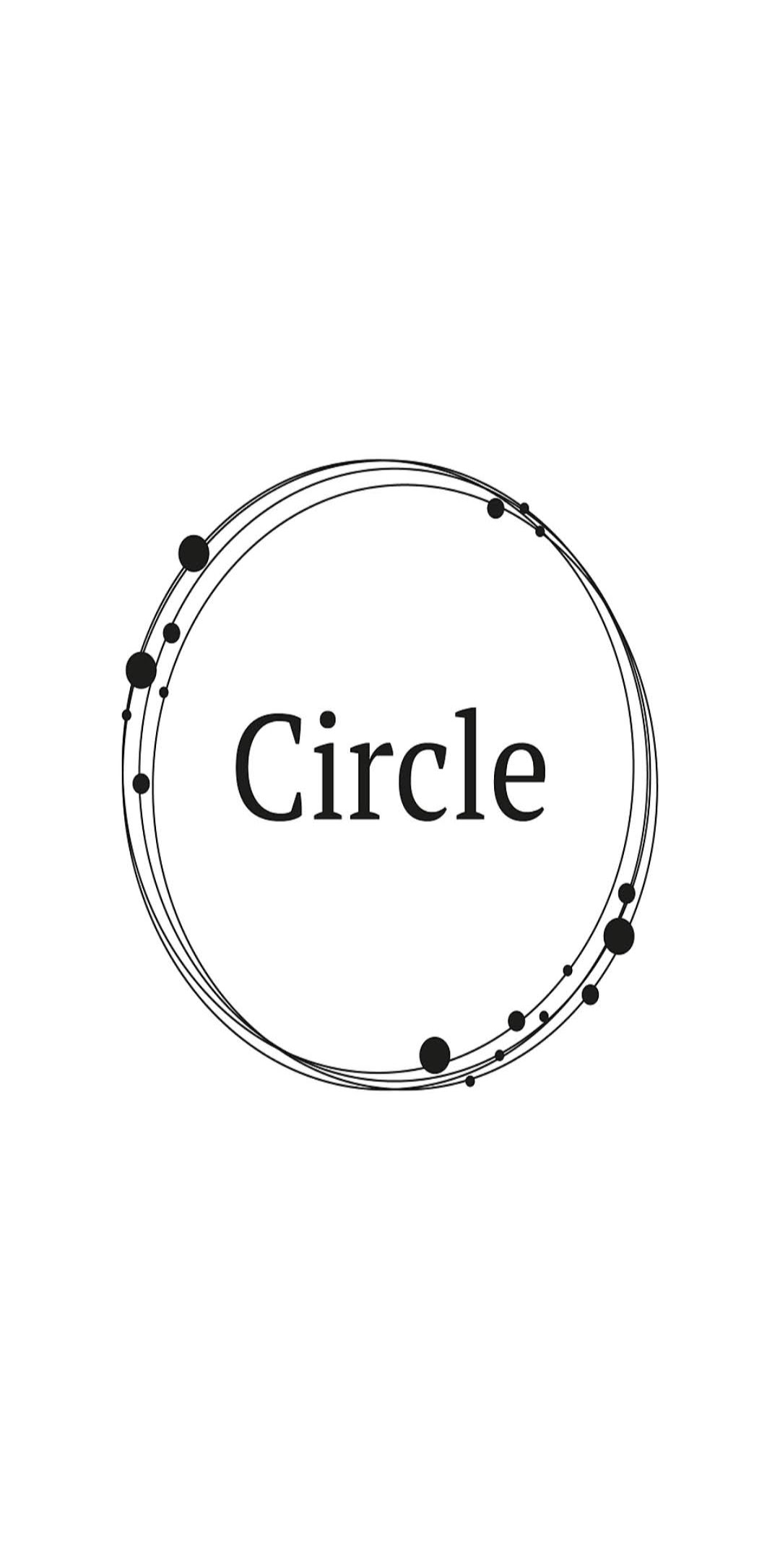

Supplement: Multimedia Appendix 5 [file formative_v7i1e42172_app5.zip › original_a0b41220-6150-4d0e-9c3f-8f7c33707b5a_Screenshot_2021-12-03-17-47-17-37_b18fac2f20e3d3dbceaad755ce160a22.jpg]

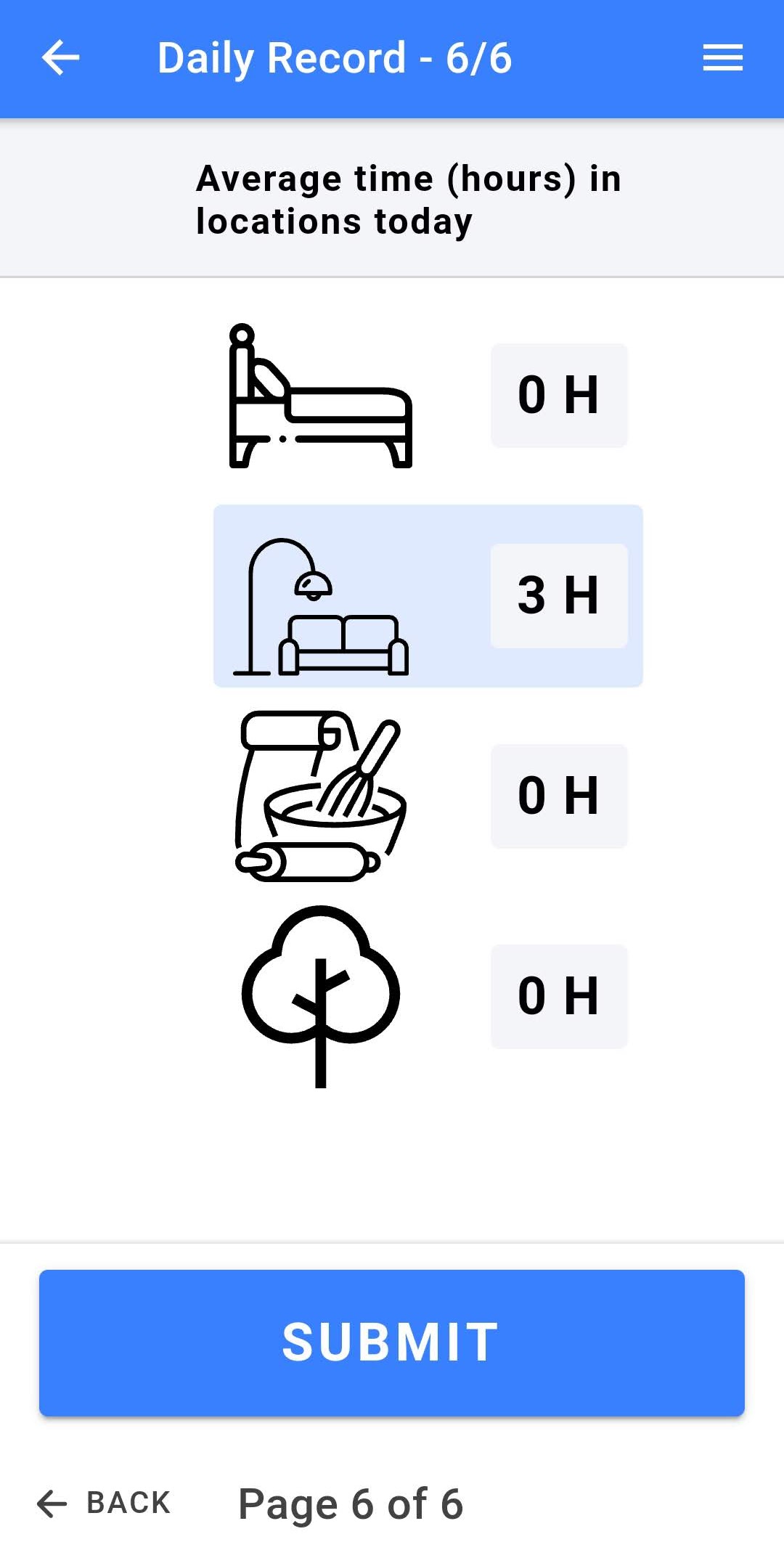

Supplement: Multimedia Appendix 5 [file formative_v7i1e42172_app5.zip › original_ce27c539-62d3-43df-8d7c-4f6b84c5d3fc_Screenshot_2021-12-03-17-40-17-25_b18fac2f20e3d3dbceaad755ce160a22.jpg]

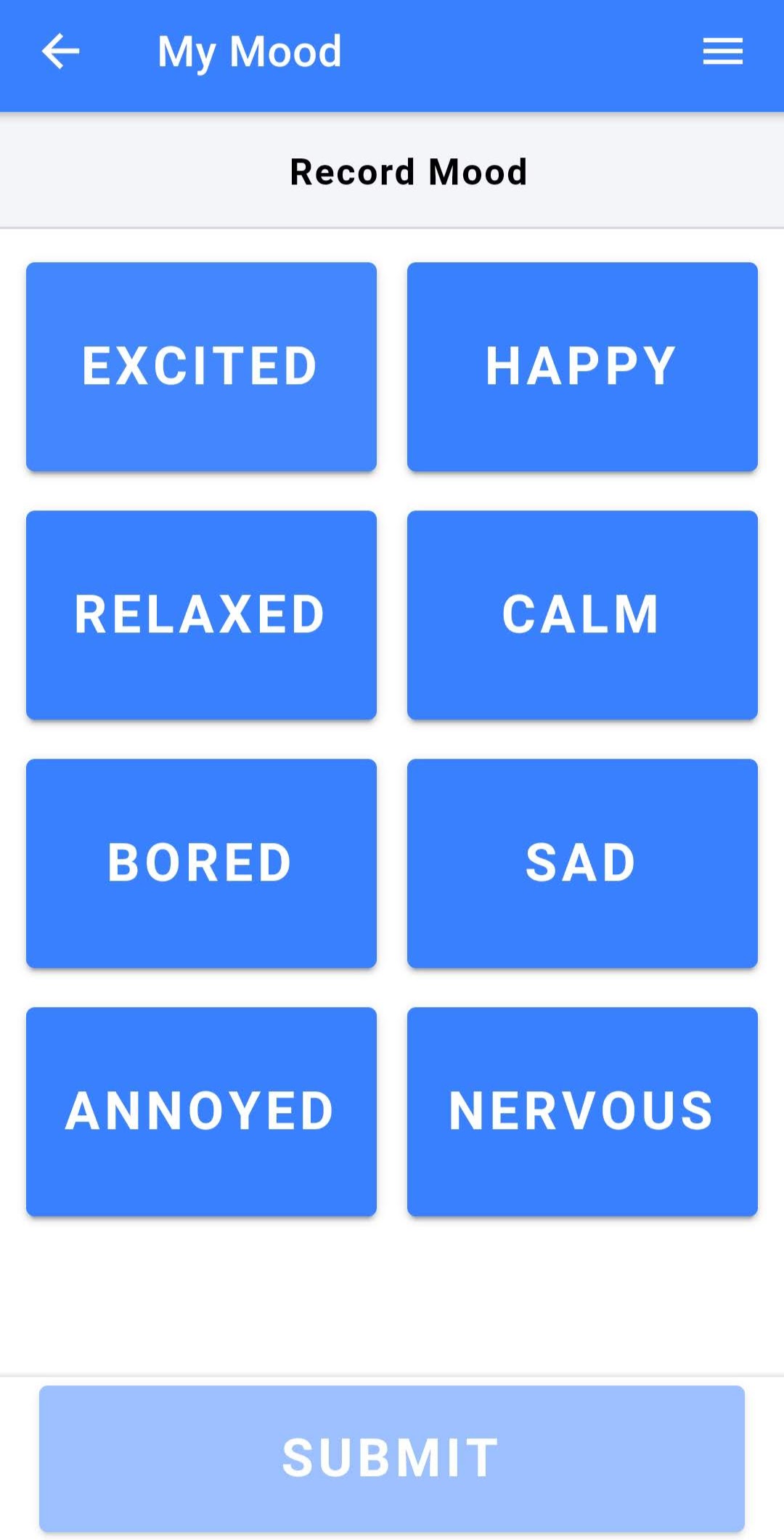

Supplement: Multimedia Appendix 5 [file formative_v7i1e42172_app5.zip › original_d095896c-1533-45e4-839c-4cc581e4e16b_Screenshot_2021-12-03-17-39-22-71_b18fac2f20e3d3dbceaad755ce160a22.jpg]

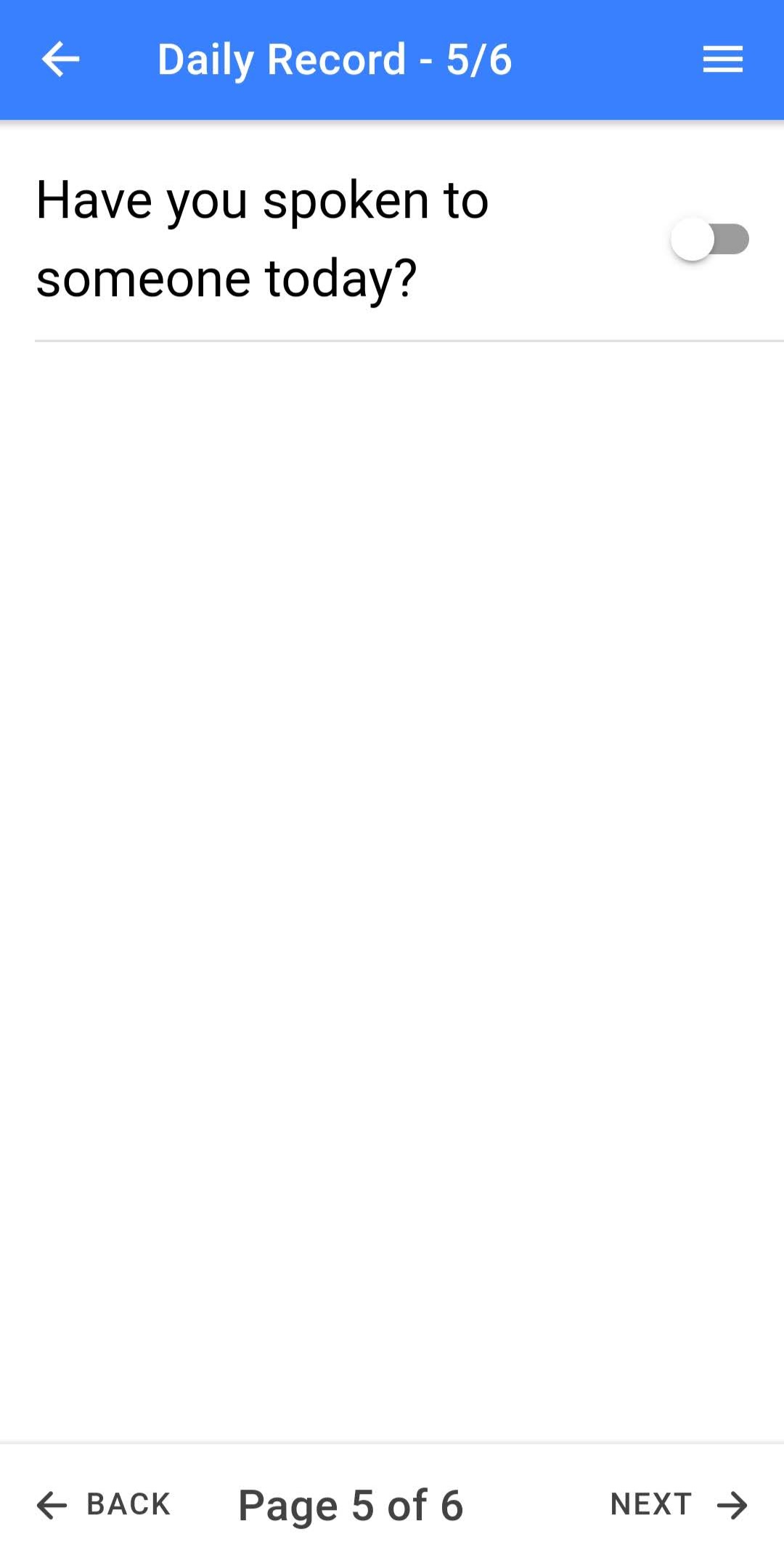

Supplement: Multimedia Appendix 5 [file formative_v7i1e42172_app5.zip › original_d76e1561-8771-4a21-81c6-93f7ef046c78_Screenshot_2021-12-03-17-39-56-04_b18fac2f20e3d3dbceaad755ce160a22.jpg]

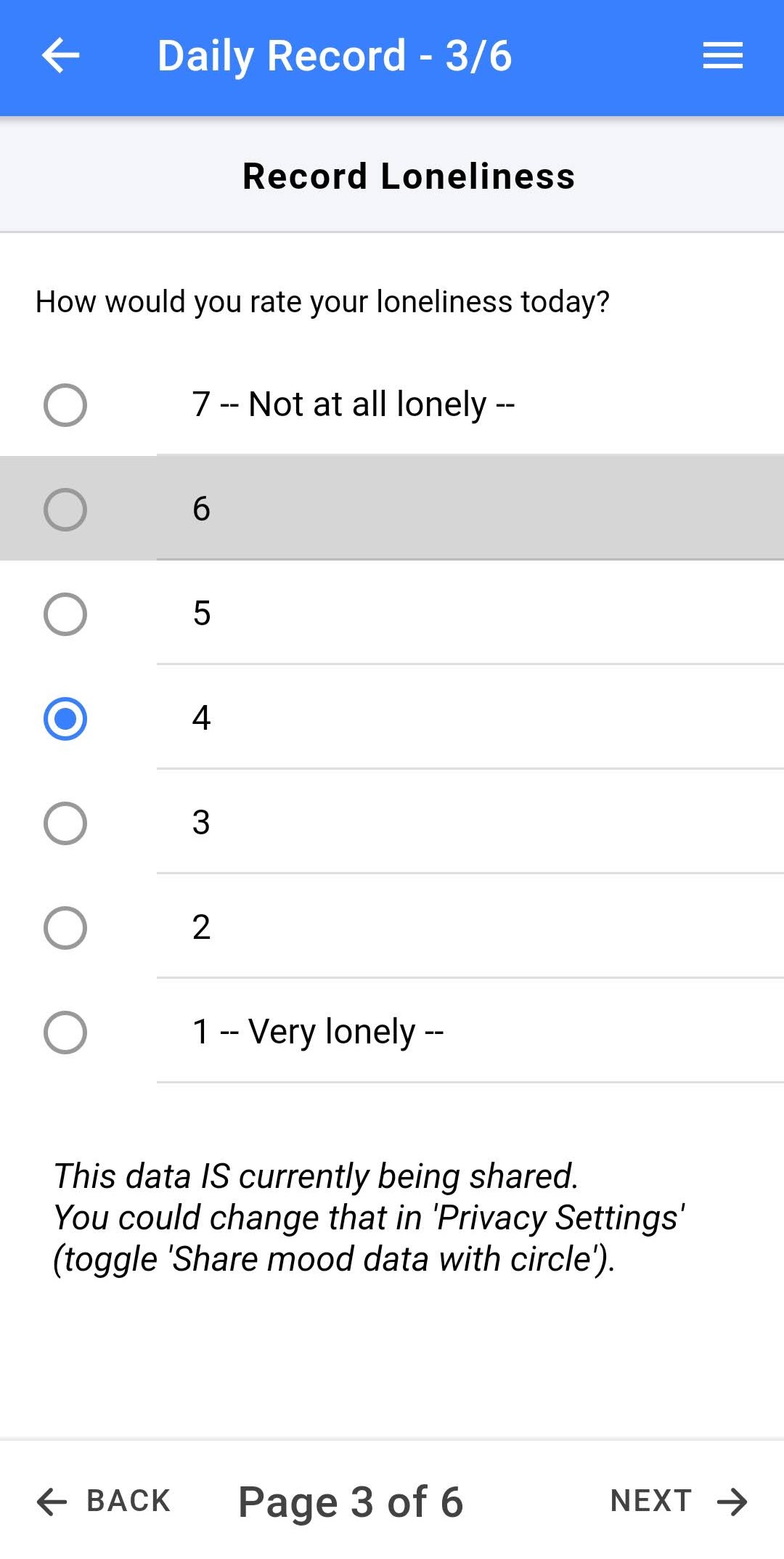

Supplement: Multimedia Appendix 5 [file formative_v7i1e42172_app5.zip › original_dbe219fc-2633-4aa6-a999-487ff5357e19_Screenshot_2021-12-03-17-39-42-63_b18fac2f20e3d3dbceaad755ce160a22.jpg]

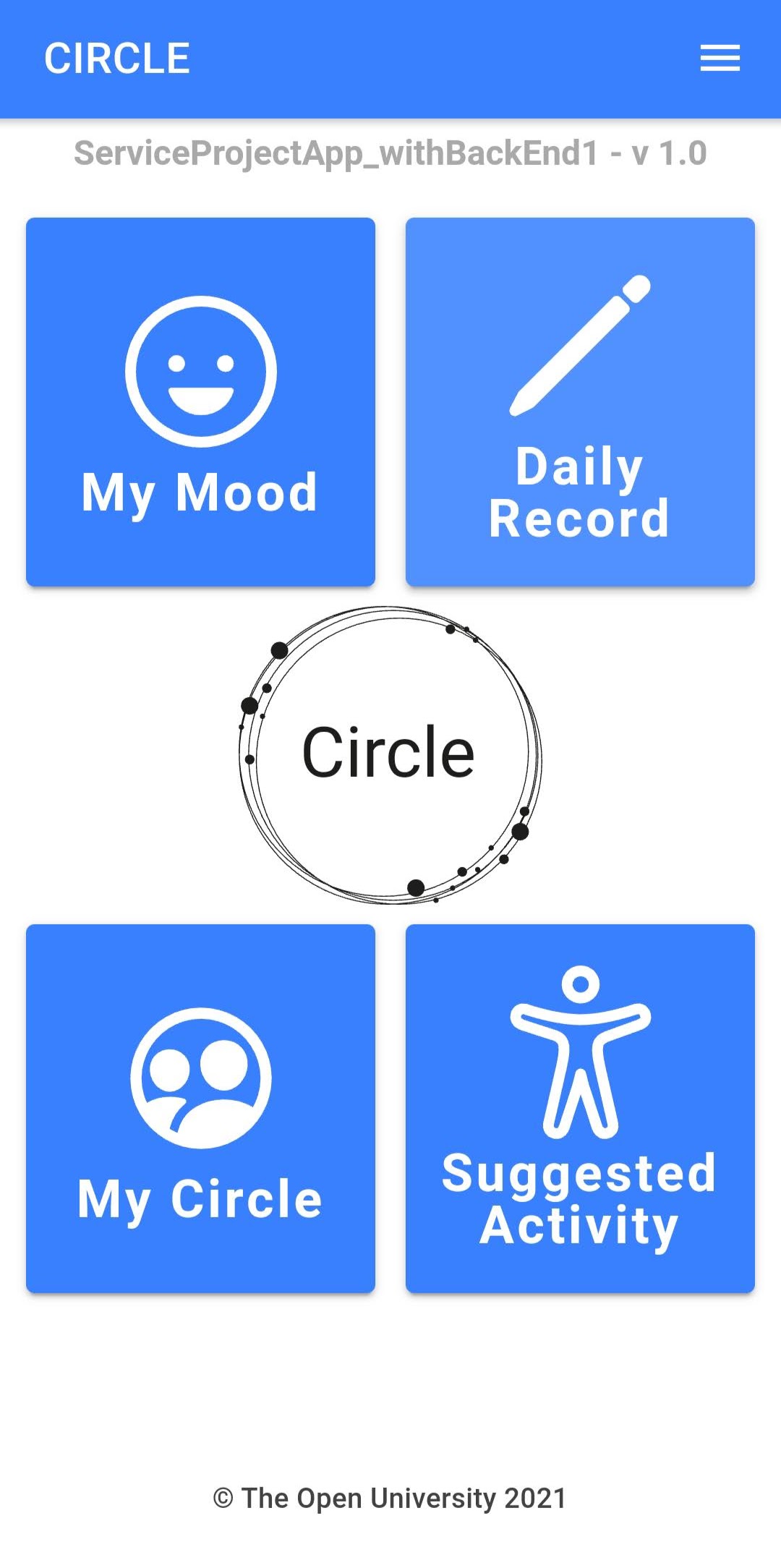

Supplement: Multimedia Appendix 5 [file formative_v7i1e42172_app5.zip › original_e312a3ba-c2d6-4bff-af85-2f53cc70a745_Screenshot_2021-12-03-17-39-18-78_b18fac2f20e3d3dbceaad755ce160a22.jpg]

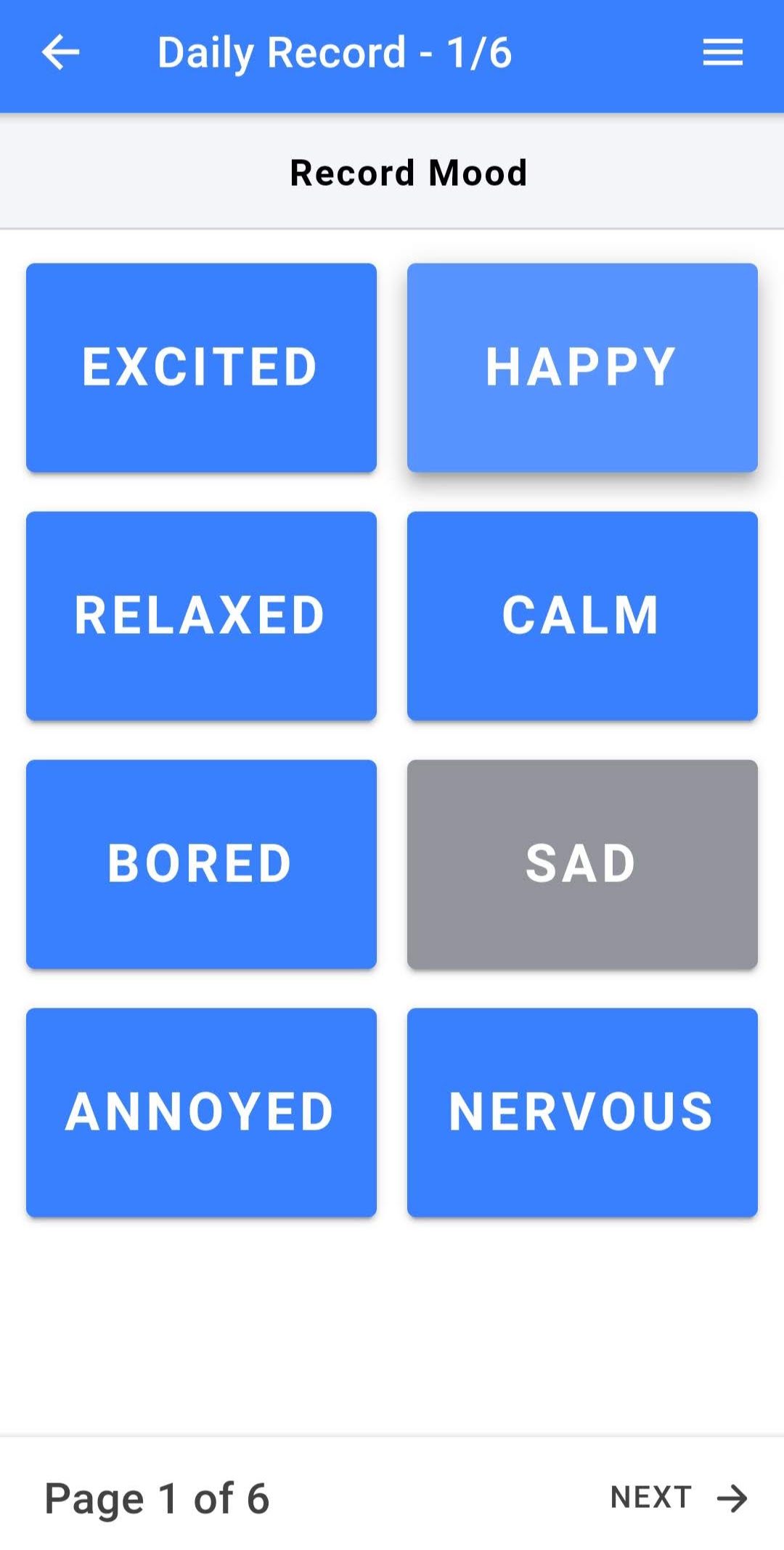

Supplement: Multimedia Appendix 5 [file formative_v7i1e42172_app5.zip › original_ef66bad3-84df-4c16-a44c-3905001f8f35_Screenshot_2021-12-03-17-39-34-13_b18fac2f20e3d3dbceaad755ce160a22.jpg]

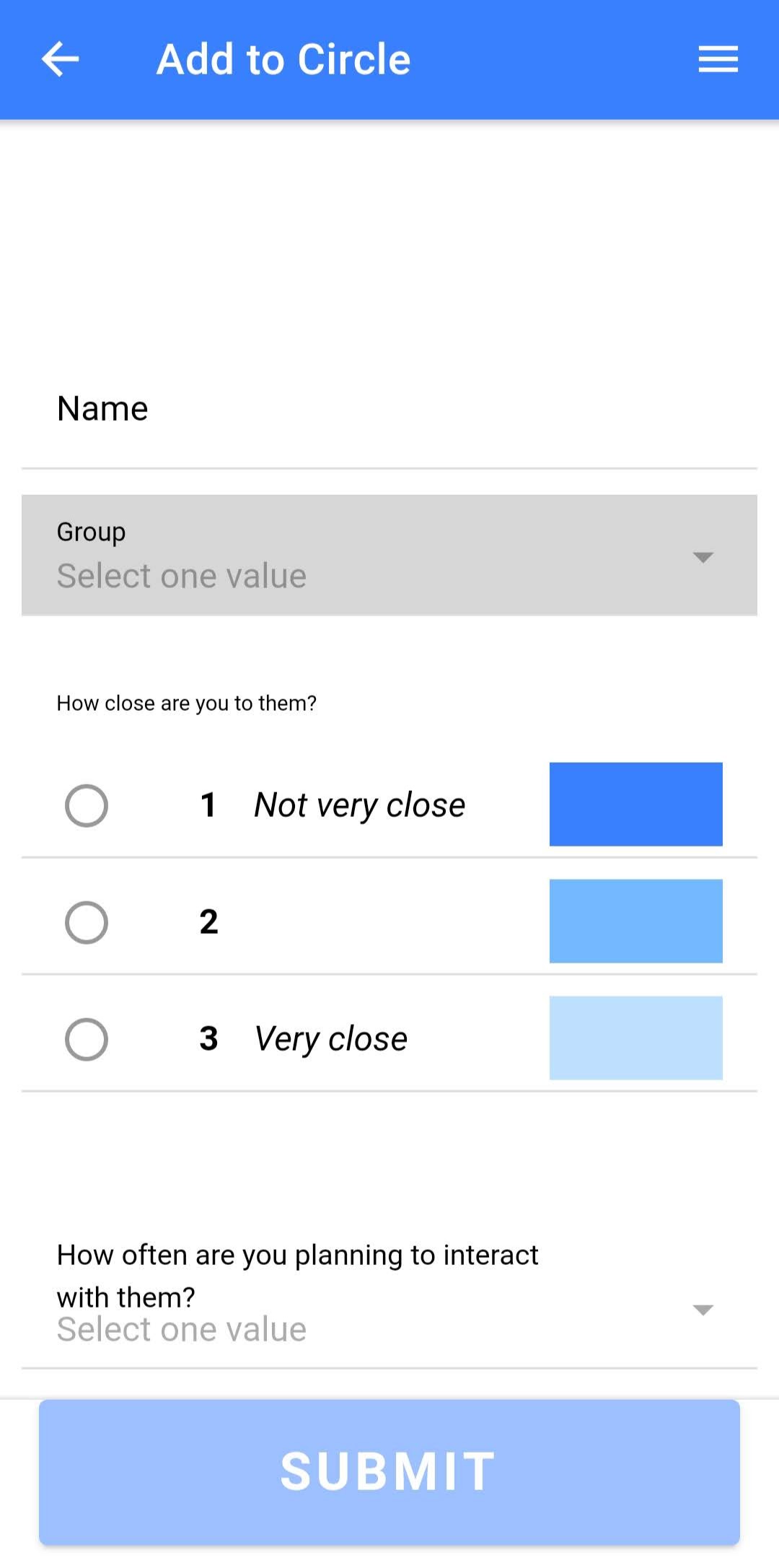

Supplement: Multimedia Appendix 5 [file formative_v7i1e42172_app5.zip › original_f32880a8-9ecf-4911-8cd2-b57760f47662_Screenshot_2021-12-03-17-40-39-87_b18fac2f20e3d3dbceaad755ce160a22.jpg]
